# Application of the 2-deoxyglucose scaffold as a new chiral probe for elucidation of the absolute configuration of secondary alcohols

**DOI:** 10.1038/s41598-022-21174-8

**Published:** 2022-10-07

**Authors:** Alicja Trocka, Katarzyna Szwarc-Karabyka, Sławomir Makowiec, Tomasz Laskowski

**Affiliations:** 1 Department of Organic Chemistry, Faculty of Chemistry, Gdańsk University of Technology, Narutowicza Street 11/12, 80-233 Gdańsk, Poland; 2 Nuclear Magnetic Resonance Laboratory, Faculty of Chemistry, Gdańsk University of Technology, Narutowicza Street 11/12, 80-233 Gdańsk, Poland; 3 Department of Pharmaceutical Technology and Biochemistry and BioTechMed Centre, Faculty of Chemistry, Gdańsk University of Technology, Narutowicza Street 11/12, 80-233 Gdańsk, Poland

**Keywords:** Chemistry, Nuclear chemistry, Organic chemistry, Techniques and instrumentation, NMR spectroscopy

## Abstract

Herein, we present the application of 2-deoxy-d-glucose derivatives as chiral probes for elucidation of the absolute configuration of chiral secondary alcohols. The probes are attached to the studied molecules via glycosylation reaction and the resulting products are examined by a set of standard 2D NMR experiments. The absolute configuration of an oxymethine carbon atom binding the probe is established on a basis of a set of diagnostic dipolar couplings (NOEs/ROEs). These correlations may be considered diagnostic due to a pronounced lack of conformational freedom of the formed glycosidic linkage. While the chance for an observation of the diagnostic signals is the highest when the resulting glycoside in an α-anomer. 2-deoxy-d-glucose was selected as a probe of choice since is it known to strongly prefer the formation of α-glycosides

## Introduction

Secondary hydroxyl group is one of the most common, yet crucial functions found in natural and synthetic organic compounds, which—in most cases—introduce chirality to the molecule. Several decades ago, Mosher and coworkers proposed a method for elucidation of the absolute configuration of secondary alcohols, which was based on the reactions of both enantiomers of MTPA with a studied molecule and careful observation of shielding/deshielding effects in NMR spectra^1^. This method, with dozens of its modifications, was used in studies of secondary alcohols and other classes of chiral molecules with limited success^2–7^. While still considered as a standard, this approach does not guarantee an unambiguous result, hence it is often burdened with dangerous assumptions, which may lead to erroneous conclusions^8,9^.

Some time ago, our group proposed a novel method of assigning the stereochemistry of secondary alcohols, which already echoed in the world literature^10–14^. It has originated from the model studies on amphotericin B; its complete historical background has been presented in our previous . Long story short, this approach is based on an observation that a glycosidic bond, formed between a pyranose and virtually any chiral aglycone, displays almost nonconformational freedom. While this is true for both alpha and beta anomers of resulting glycosides, in alpha anomers the pyranose probe settles itself closer to the aglycone, which enables an observation of diagnostic dipolar couplings in proton NMR spectra.

To this date, our group has proven the applicability of unmodified D-glucose, D-mannose and L-rhamnose for elucidation of the absolute configuration of 2-butanol^15^. In these studies, only the alpha anomers were proven to be useful. Later on, tetra-O-benzyl derivative of D-mannose has yielded a successful stereochemical study on several natural compounds^16^. While in case of ( +)-menthol both alpha and beta anomers displayed diagnostic dipolar couplings, the formation of beta glycosides is generally being considered as a complication, which desimplifies the process of synthesis, isolation and NMR studies.

In a constant search for the best possible sugar probe, our attention was drawn towards to 2-deoxyglucose. While the general chemistry of this hexose is both compelling and not fully understood, it is known that the synthesis of beta glycosides of this monosaccharide is rather challenging^17,18^, hence beta-anomers by any means do not manifest as significant by-products of glycosidation process. Therefore, in this study, we have examined the process of formation of tri-O-benzyl and tri-O-benzoil derivatives of 2-deoxyglucose and their application as chiral probes for elucidation of the absolute configuration of chiral secondary alcohols.

**Figure 1:**
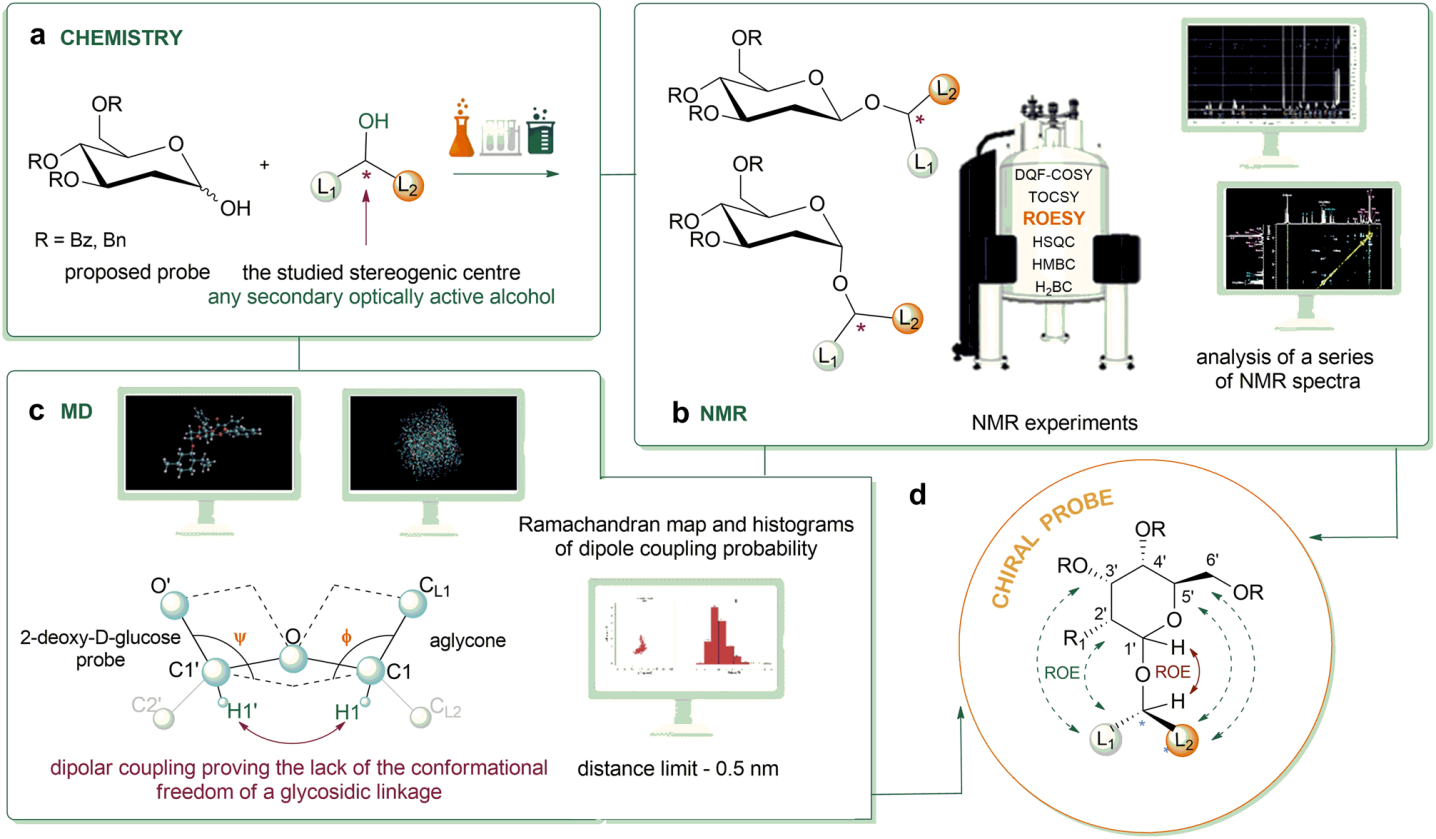
The illustration introduces the general concept of the method: (**a**) synthesis of the proposed probe and its reaction with a selected chiral secondary alcohol; (**b**) NMR experiments and analysis of one- and two-dimensional spectra; (**c**) computer simulation of the probe-aglycon system with selected data related to dipole connections and definition of double-walled angles Φ and Ψ; (**d**) comparison of NMR results with MD.

**Figure 2:**
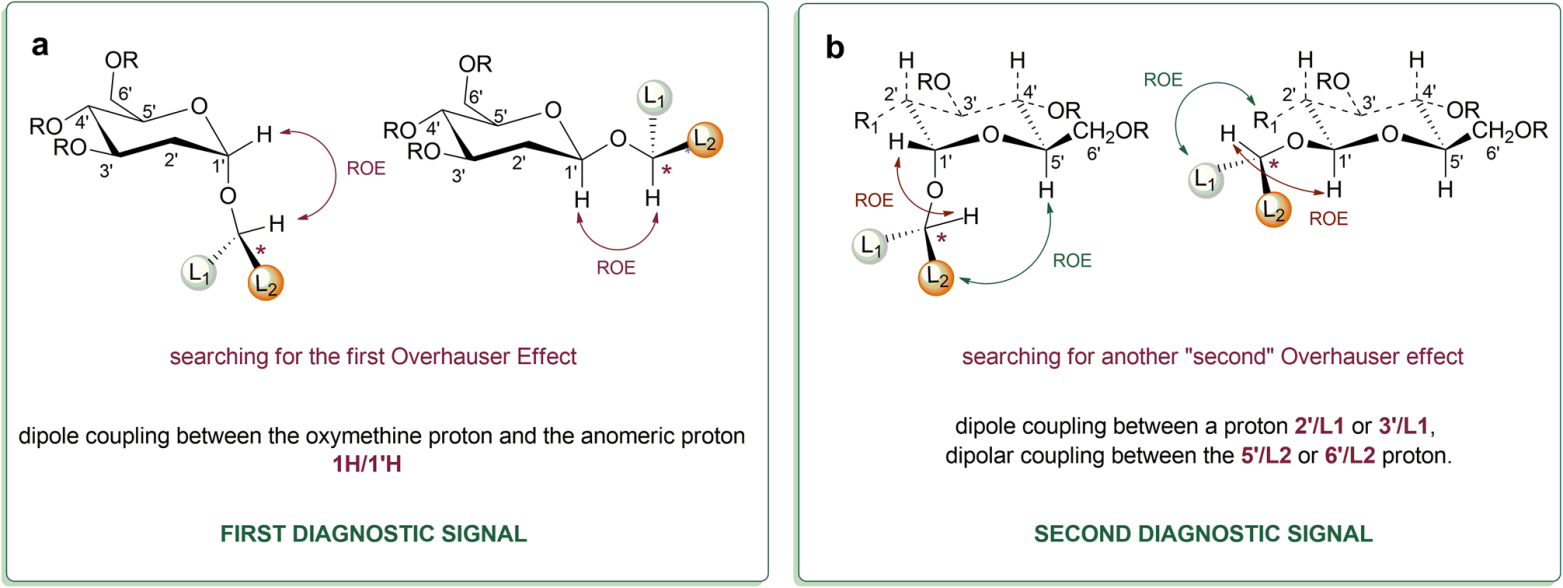
Determination of the absolute configuration of secondary alcohols with the use of dipole couplings: (**a**) first diagnostic signal; (**b**) second diagnostic signal.

In the proposed concept, the sugar motif—in the case of 2-deoxyglucose—play the role of a stereochemically defined molecular probe introduced into a selected, chiral secondary alcohol by creating a bond. One of that bond’s component includes the tested asymmetric carbon. The usefulness of the method can be checked on the proposed models of chiral secondary alcohols such as isomers (1S,2R,5S)-( +)-menthol, (-)-borneol and (S)-2-butanol. The concept of the proposed method assumes a chemical synthesis related to the formation of an O-glycosidic bond between the probe and the aglycone, followed by a series of 2D NMR spectroscopic studies—including the observation of Overhauser effects between the protons of the carbohydrate units, alcohol units and the use of molecular modeling techniques to simulate the interactions of the created systems. Below, we have demonstrated the general concept of the proposed method (Fig. 1):

Earlier studies on polyene macrolides and chiral probes with the use of the D-mannose scaffold allowed for the registration of dipole couplings, which appear most often between monosaccharides and aglycones^15,16^. The general methodology for determining the configuration of secondary alcohols involves the search for a minimum of two diagnostic signals—homonuclear dipolar couplings. The first diagnostic signal that should appear in the ROESY spectrum relates to the interaction in the **1'H**/**1H** proton space. Presence of this link indicates an inhibition of rotational freedom of connections around the glycosidic bond (Fig. 2a). The consequence of observing the above-mentioned dipole coupling is the search for more Overhauser effects. Possible spatial interactions between the protons of **L1** and **L2** ligands and the monosaccharide are presented in (Fig. 1d). On the left side of the tested molecule, protons on **2'H** and **3'H** can couple only with **L1**, while on the right—protons **5'H** and possibly **6'H** are able to interact in a dipole with **L2**. The possible ROE effects presented above will never appear all in ROESY experiments, although one of them is enough to establish mutual orientation. Dipolar couplings of **2'H** and **5'H** protons with ligands of the rest of the alcohol are most often observed (Fig. 2b). As a result of confirmation of a specific glycoside projection, the subsequent interprotonic interrelationships sought are related to determining the location of particular ligands. Later in this article, we present studies in which interprotonic interactions between probe and aglycone were observed using the 2-deoxy-d-glucose scaffold.

## Results and discussion

### Chemistry

**Figure 3:**
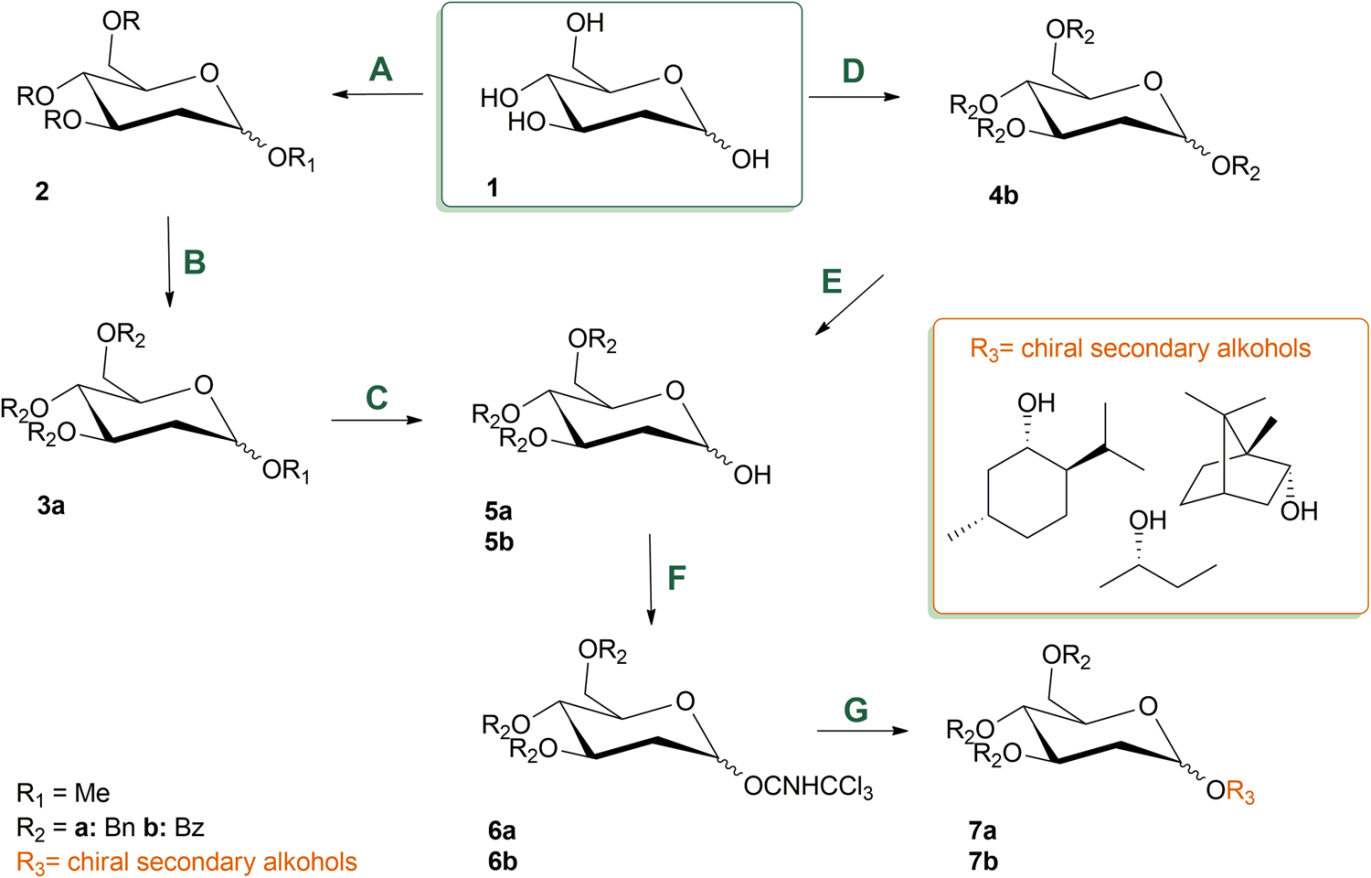
General procedures of synthesis of chiral probes: (path **A**) formation of o-methyl glycoside: MeOH, 1% AcCl / 24 h, RT; (path **B**) benzylation: BnBr, DMF, NaH / atm Ar, 18 h, 0 °C; (path **D**) benzoylation: BzCl, DMAP, Py / atm Ar, 72 h, RT ; (path **C**/**E**) removal of the group from the anomeric carbon: C AcOH, HCl / 1 h, 55 °C; E 1. 45% HBr/NOAc / atm Ar, 2.5 h, RT 2. Ag_2_CO_3_, acetone, H_2_O / 1 h, RT; Schmidt method: (path **F**) trichloroacetonitrile O-activation: Cl_3_CCN / base, (path **G**) O-glycosidication with chiral secondary alcohol R_3_OH / acid.

**Figure 4:**
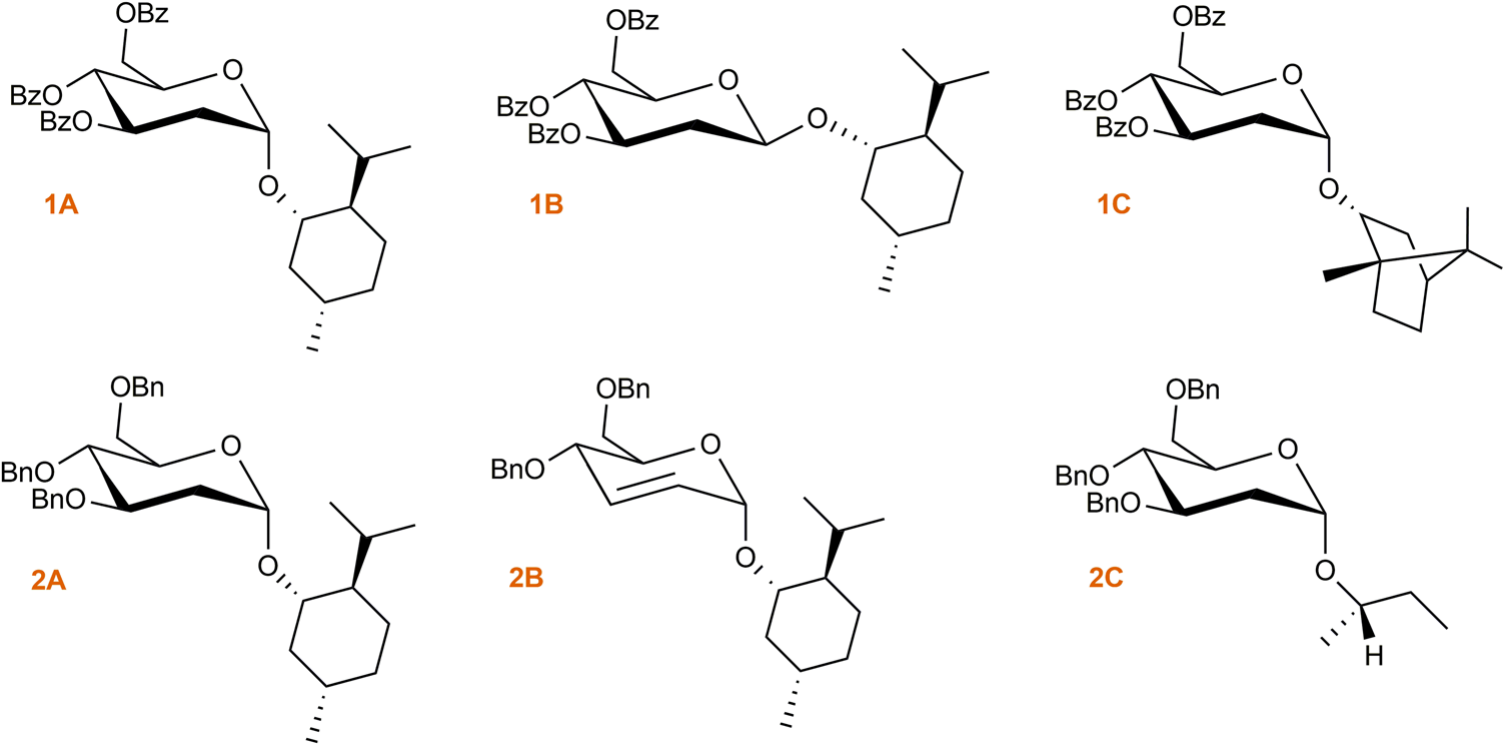
Received O-glycosides **1A**, **1B**, **1C**, **2A**, **2B** and **2C.**

Chiral glycoside probes were prepared in two versions, the first one classic, with benzyl protection of the hydroxyl groups of 2-deoxyglucose and the second one using less typical benzoyl protecting groups (Fig. 3). Probes with benzyl protection were prepared in the usual manner. In the first step methyl 2-deoxyglucoside was prepared, which was then alkylated with benzyl bromide. Anomeric methyl group was removed in mild acidic condition to obtain compound **5a**, ready for the final coupling with chiral secondary alcohol. In the case of benzoylated sugars we applied a two-step approach, with benzoylation of all hydroxyl groups in the first step following by selective deprotection of anomeric position. In this step, we tried to apply the deprotection with benzyl amine or ethanolamine, however this proved to be unsuccessful. This turned out to be the method of choice for two step but one-pot deprotection with transformation to glycosyl bromide and subsequent hydrolysis assisted with silver cations leading to formation of **5b**. In the last step, we applied the Schmidt method. First step was the O-activation of the monosaccharide derivative with trichloroacetonitrile in presence of catalytic amounts of a strong base. The second step of the Schmidt reaction was formation of an O-glycosidic linkage. For this purpose, we used commercially available optically active secondary alcohols such as (1S,2R,5S)-( +)-menthol, (-)-borneol and (S)-2-butanol. The reactions were carried out in the presence of catalytic amounts of acid. Due to significant steric hindrance of used alcohols we experienced low yield of prepared probes. Additionally, in one case we isolated dehydrated glycoside **2B** which also turned out to be useful for spectroscopic studies. As a result of chemical syntheses, **1A**, **1B**, **1C**, **2A**, **2B** and **2C** were obtained (Fig. 4).

### Nuclear magnetic resonance

Compound **1A**, **1B**, **1C**, **2A**, **2B** and **2C** were subjected to NMR studies in acetone-d_6_ or benzene-d_6_. Using standard DQF-COSY, TOCSY, HSQC, nd-HSQC, HMBC, and ROESY experiments, all of the connections in each isolated spin system of the resulting glycosides were traced in an uncomplicated manner The detailed procedure of the absolute configuration assignment by NMR will be presented on the instance of the molecule **1A**.

#### Discussion of NMR results for 1A and 1B

**Figure 5:**
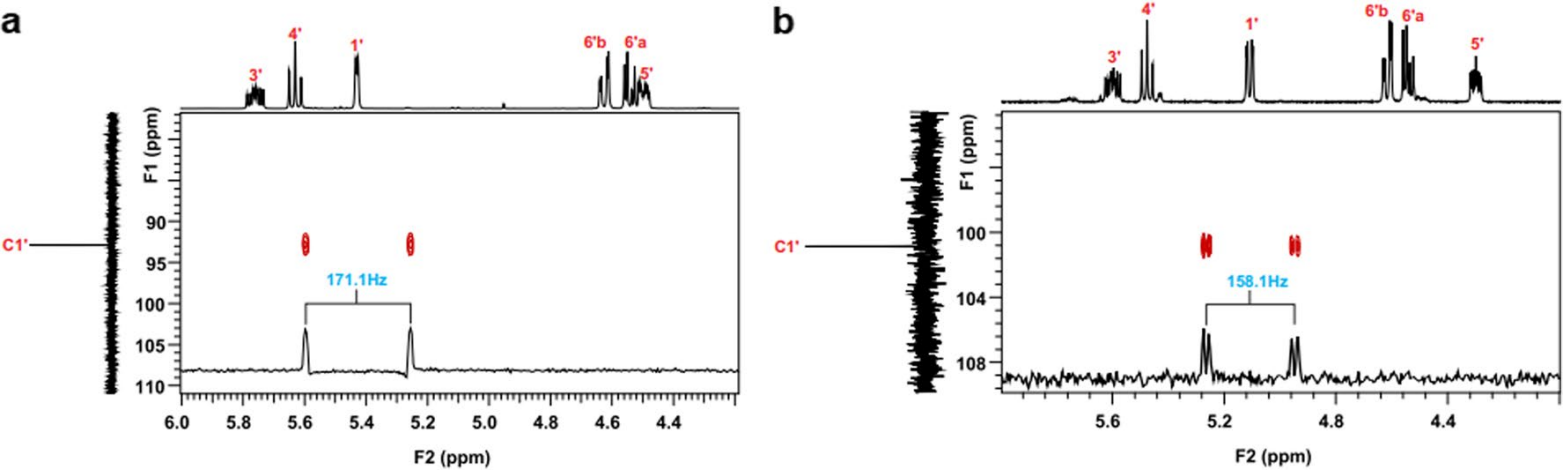
nd**-**HSQC spectra with the value of the ^1^J C–H coupling constant for: (**a**) molecule **1A**—alpha anomer; (**b**) **1B** molecule—beta anomer.

In the nd-HSQC spectrum the coupling constant ^1^J C-H of the **1A** molecule is 171.1 Hz (Fig. 5a and Fig. S6), which is synonymous with obtaining the alpha anomer. For the beta anomer **1B**, a ^1^ J C-H value of 158.1 Hz is observed (Fig. 5b and Fig. S15)^19^. An additional factor which confirms the glycoside conformation is the high chemical shift of the C1' (Table 1) signal in one-dimensional spectra. The **1'H** proton shift (Table 2) is due to the effect of shielding the ester group -COPh.

**Table 1:** Chemical carbon shifts for molecule **1A.**

^13^C NMR Data for **1A**		
Position		^13^C δ (ppm)
**Aglycone Unit**		
**1**	CH	75.0
**2**	CH	48.1
**3**	CH_2_	22.7
**4**	CH_2_	34.3
**5**	CH	31.2
**6**	CH_2_	39.4
**7**	CH	25.3
**8**	CH_3_	15.2
**9**	CH_3_	20.8
**10**	CH_3_	21.8
**Probe Moiety**		
**1'**	Anomeric carbon	92.9
**2'**		35.8
**3’**		70.2
**4'**		70.6
**5'**		68.9
**6'**		63.3
**O-Bz**	18 aromatic carbons	129.0–133.4
**C = O**	3 carbonyl carbons	165.2
		165.3
		165.6

**Table 2:** Chemical proton shifts and coupling constants for molecule 1A second.

^1^H NMR Data for **1A**		
Position	^1^H δ (ppm)	J_H,H_ (Hz)
**Aglycone Unit**		
**1**	3.61	10.7 (**2**), 10.7 (**6ax**), 4.1 (**6 eq**)
**2**	1.39	10.7 (**1**), 11.1 (**3ax**), (**3 eq**)*, 2.6 (**7**)
**3ax**	1.06	11.1 (**2**), 13,1 (**3 eq**), (**4ax**)*, (**4 eq**)*
**3 eq**	1.72	(**2**)*, 13.1 (**3ax**), (**4ax**)*, (**4 eq**)*
**4ax**	0.92	(**3ax**)*, (**3 eq**)*, 12.3 (**4 eq**), 12.1 (**5**)
**4 eq**	1.71	(**3ax**)*, (**3 eq**)*, 12.3 (**4ax**), 5.7 (**5**)
**5**	1.39	12.1 (**4ax**), 5.7 (**4 eq**), (**6ax**)*, (**6 eq**)*, 7.0 (**10**)
**6ax**	0.89	10.7 (**1**), (**5**)*, 12.4 (**6 eq**)
**6 eq**	2.25	4.1 (**1**), (**5**)*, 12.4 (**6ax**)
**7**	2.49	2.6 (**2**), 7.0 (**8**), 7.0 (**9**)
**8**	0.89	7.0 (**7**)
**9**	1.01	7.0 (**7**)
**10**	0.95	7.0 (**5**)
**Probe Moiety**		
**1’**	5.43	0.8 (**2’eq**), 4.2 (**2’ax**)
**2’ax**	2.18	4.2 (**1’**), 12.9 (**2’eq**), 12.0 (**3’**)
**2’eq**	2.46	0.8 (**1’**), 12.9 (**2’ax**), 5.6 (**3’**)
**3’**	5.76	12.0 (**2’ax**), 5.6 (**2’eq**), 9.9 (**4’**)
**4’**	5.63	9.9 (**3’**), 9.9 (**5’**)
**5’**	4.49	9.9 (**4’**), 4.6 (**6’a**), 2.4 (**6’b**)
**6’a**	4.54	4.6 (**5’**), 12.1 (**6’b**)
**6’b**	4.62	2.4 (**5’**), 12.1 (**6’a**)
**O-Bz (15H)**	7.43–8.09	–

*These coupling constants could not be measured. Signal pattern remains partially unclear due to serve signal overlap and higher order effects.

The analysis of the **1A** molecule confirmed that the substituents of the cyclohexane ring (aglycone) are in equatorial positions. Interpretation of vicinal constants of the coupling and dipolar couplings allowed to define (C1-(S*), C3-(R*), C5-(S*)) absolute configuration.

#### ROE diagnostic signals

**Figure 6:**
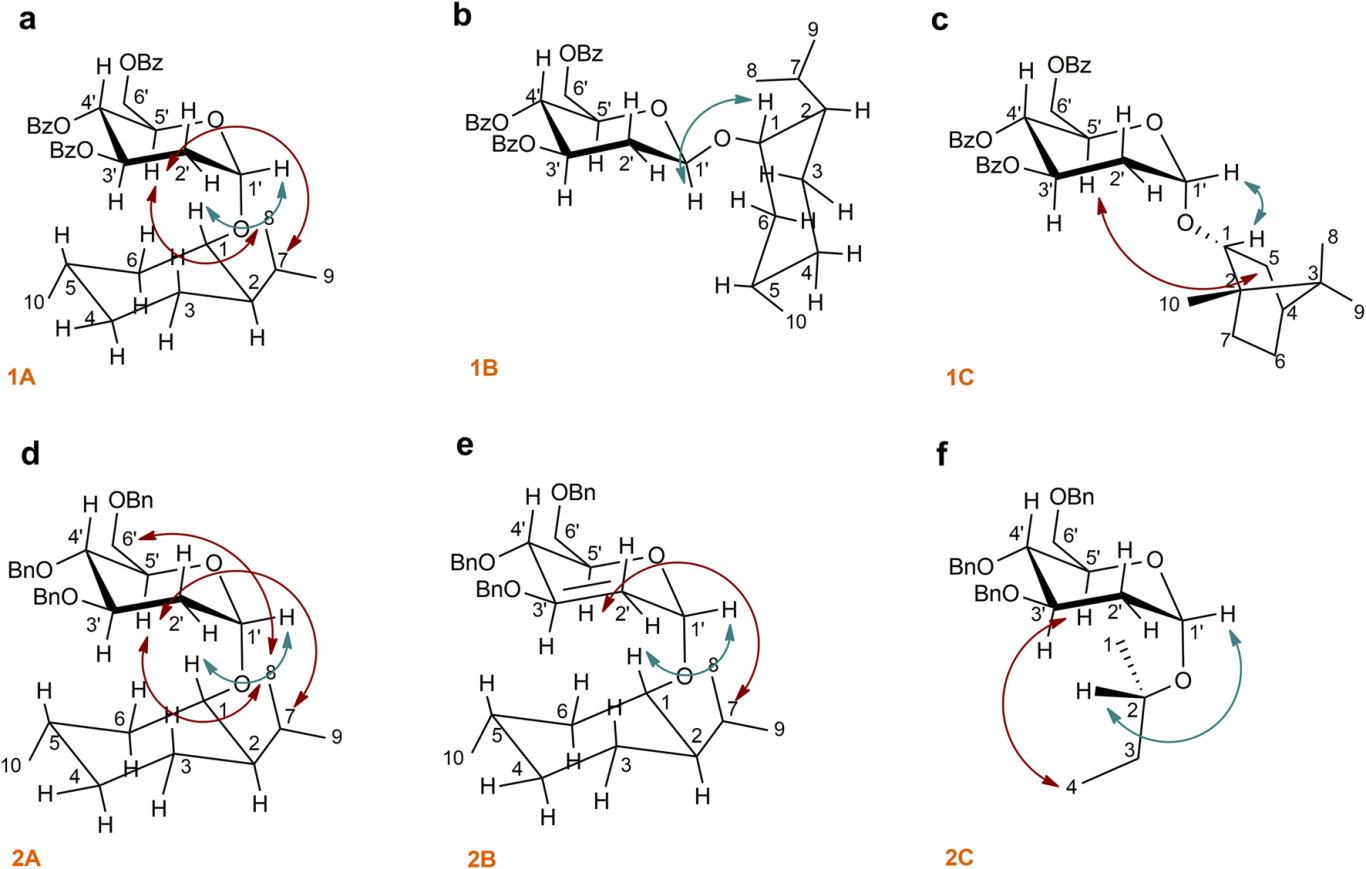
Determination of S*/R* the absolute configuration of **1A**, **1B**, **1C**, **2A**, **2B** and **2C**. The diagnostic ROEs are depicted as bidirectional arrows (blue—first diagnostic signal **1’H**/**1H** of **1A—2C**; red—second diagnostic signal for **1A 5’H**/**7H**, **5’H**/**8H**; **1C 5’H**/**5ex**; **2A 5’H**/**8H**, **5’H**/**7H**, **6’aH**/**8H**; **2B 5’H**/**7H** and **2C 5’H**/**4H**).

**Figure 7:**
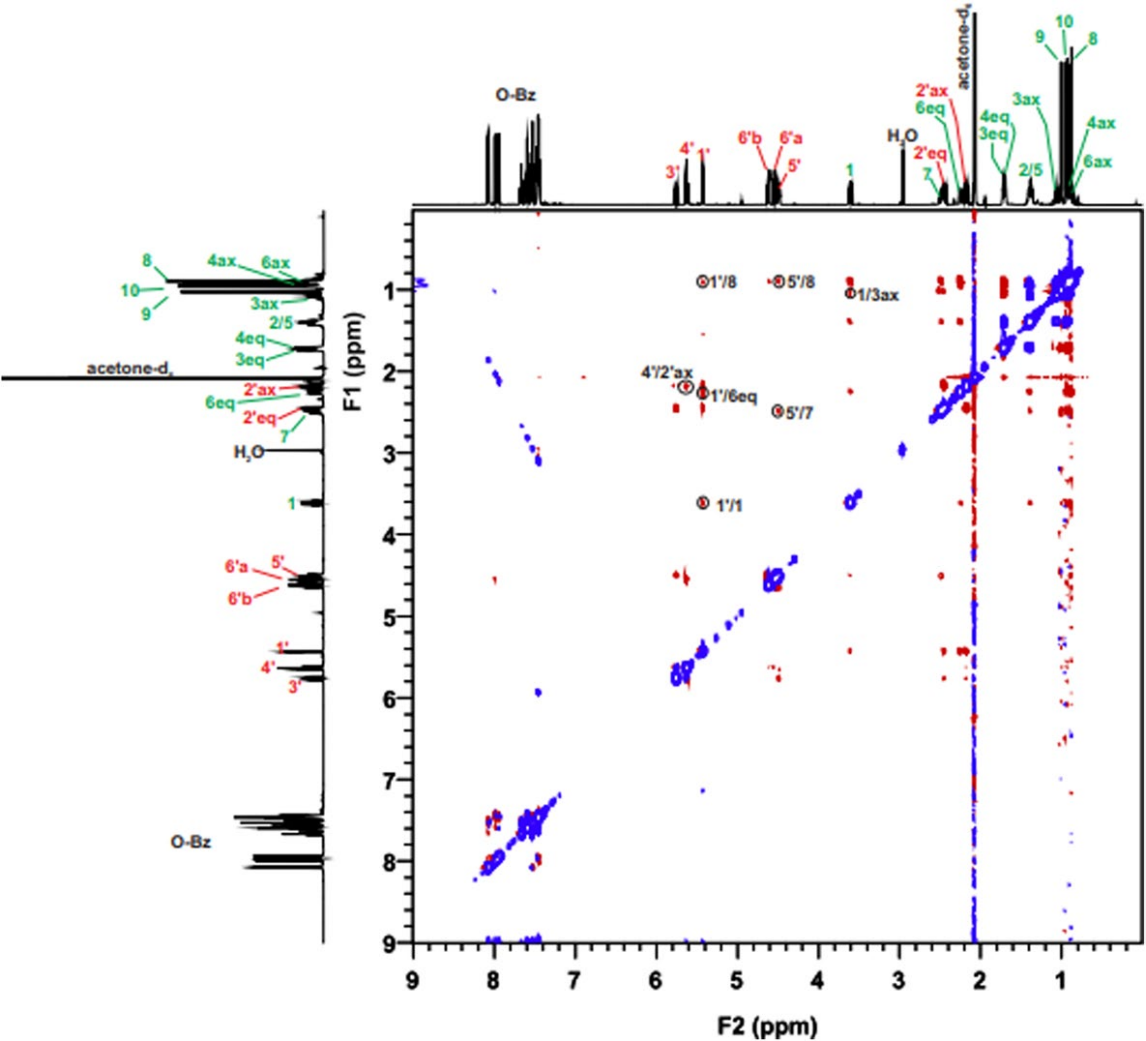
ROESY spectrum of molecule 1**A** with all dipole couplings (diagnostic signals are marked in a circle).

The probe—aglycone ROEs proved the first diagnostic signal—**H’1**/**H1**. This dipolar coupling between the anomeric proton and the proton of oximetine must occur because it indicates the partial inhibition around the O-glycosidic bond. In addition, for **1A**, two more ROEs between the protons of the aglycone unit and the protons of the chiral probe were observed, i.e., **5’H**/**7H** and **5’H**/**8H**—diagnostic signal (Table 3). ROESY NMR experiment confirmed the observed correlations in space simulated with the use of MD techniques (Fig. S49). These mentioned ROEs enabled the determination of the absolute configuration of **1A** (Fig. 6a). All of the dipolar couplings for the **1A** molecule can be observed in the ROESY spectrum (Fig. 7).

**Table 3:** Diagnostic ROE to proton for 1A compared to 1B.

2D ROESY NMR Data for **1A**		2D ROESY NMR Data for **1B**	
Position	Diagnostic ROE to proton	Position	Diagnostic ROE to proton
**Probe Moiety**			
**1’**	1, 6 eq, 8	**1’**	1, 8, 3’, 5’, 6 eq, 7
**3’**	2’eq	**3’**	2’eq, 5’, 1’
**4’**	2’ax	**4’**	2’ax
**5’**	7, 8	**5’**	1’, 3’

**Figure 8:**
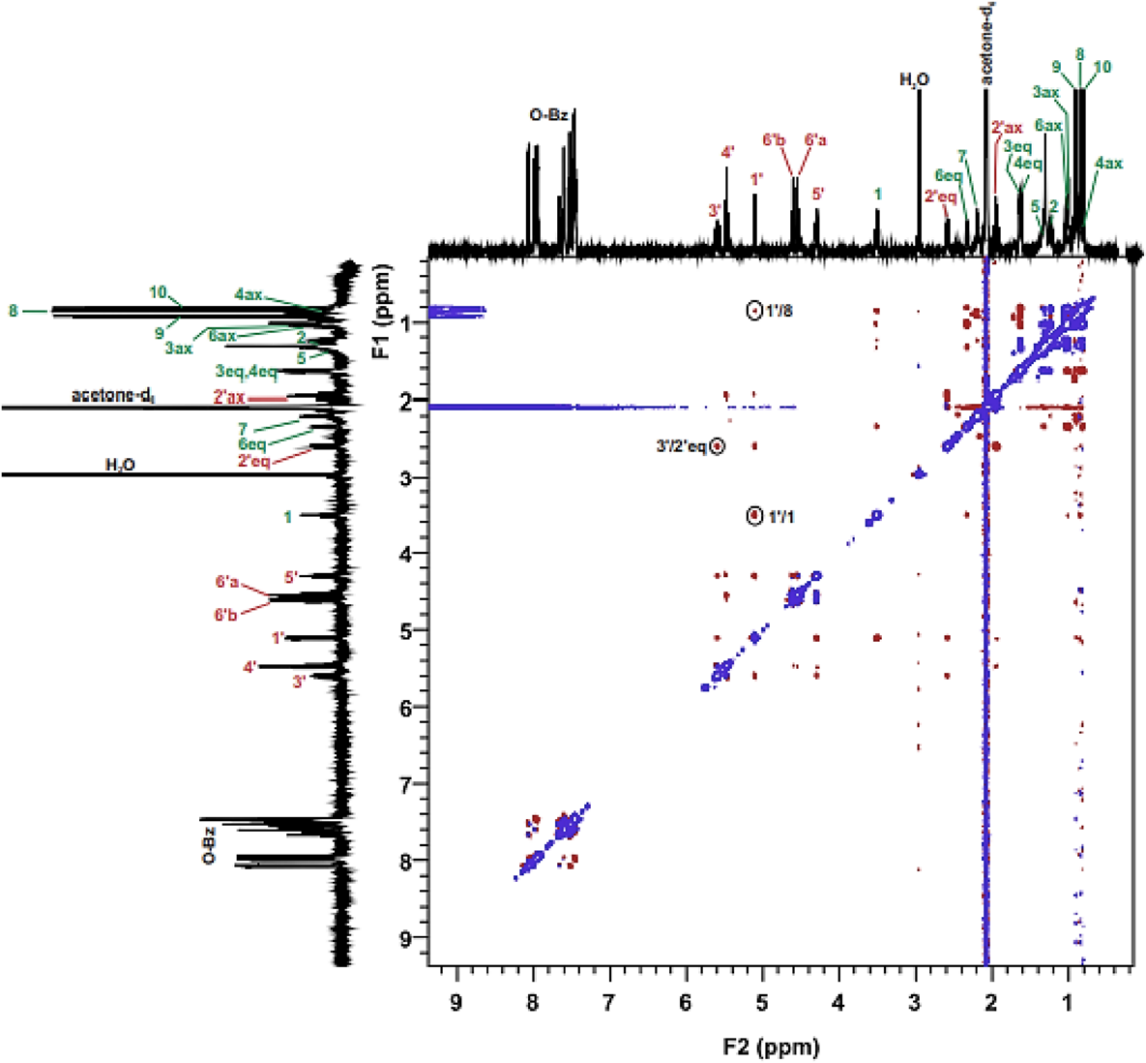
ROESY spectrum of molecule **1B** with all dipole couplings (diagnostic signals are marked in a circle).

In the case of beta **1B** anomer, the ROESY spectrum revealed that a **1'H**/**1H** signal indicative of partial inhibition of O-glycosidic bonding is present (Table S6). However, the beta anomer is not suitable as a chiral probe for determining the absolute configuration for molecule **1B** because other diagnostic correlations between probe and aglycone are unobservable (Fig. 6b and Fig. S17). All of the dipolar couplings for the **1B** molecule can be observed in the ROESY spectrum (Fig. 8). For molecule **1B**, computer simulation results were also confirmed by spectroscopic studies (Fig. S51).

#### Discussion of NMR results for 2C

**Figure 9:**
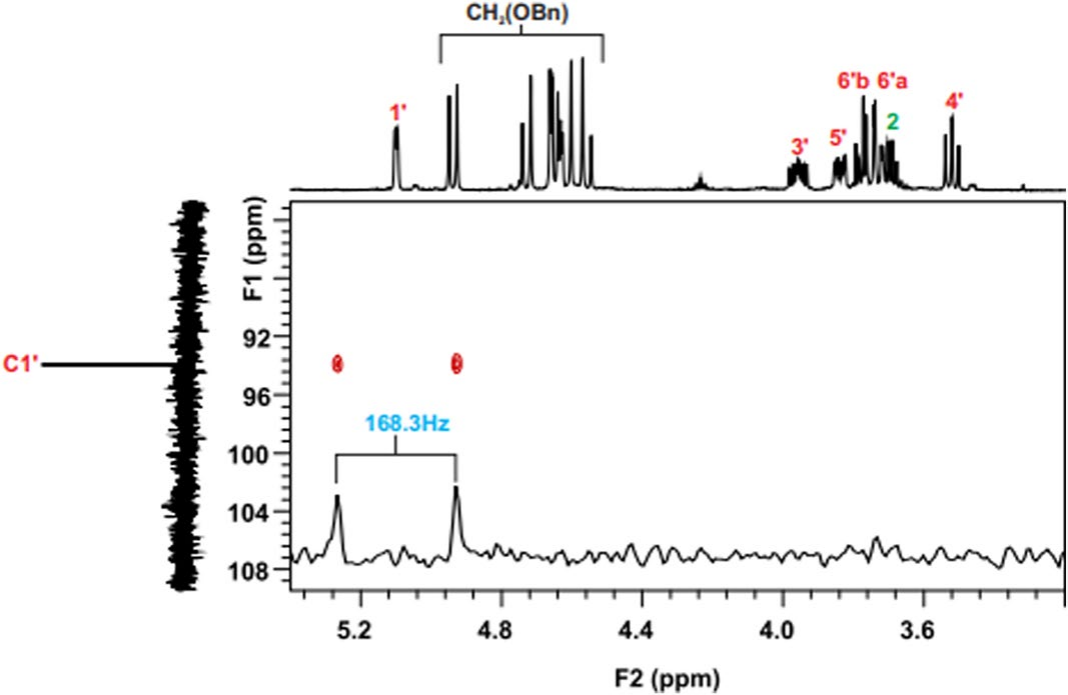
HSQC-nd spectra with the value of the ^1^J C–H coupling constant for molecule **2C**—alpha anomer.

According to nd-HSQC spectrum, the obtained compound **2C** is alpha anomer. The value of coupling constant ^1^J C-H is 168.3 Hz (Fig. 9 and Fig. S47). Aglycone signals—in this case—simple, small 2-butanol molecule are concentrated in a separate area of the chemical shift scale then protons and carbon signals derived from the benzyl derivative of 2-deoxy-d-glucose (Fig. S42) (Fig. S43). **C2** and **2H** chemical shift of the aglycone is the highest because it is a component of the O-glycosidic linkage (Tables 4, 5).

**Table 4:** Chemical proton shifts and coupling constants for molecule 2C second.

^1^H NMR Data for **2C**		
Position	^1^H δ (ppm)	J_H,H_ (Hz)
**Aglycone Unit**		
**1**	1.11	6.1 (**2**)
**2**	3.70	6.1 (**1**), 6.1 (**3a**), 6.1 (**3b**)
**3a**	1.48	6.1 (**2**), 14.0 (**3b**), 7.5 (**4**)
**3b**	1.55	6.1 (**2**), 14.0 (**3a**), 7.5 (**4**)
**4**	0.91	7.5 (**3a**), 7.5 (**3b**)
**Probe Moiety**		
**1'**	5.10	3.6 (**2’ax**), ~ 0.8 (**2’eq**)
**2’ax**	1.64	3.6 (**1’**), 12.3 (**2’eq**), 12.3 (**3’**)
**2’eq**	2.27	~ 0.8 (**1’**), 12.3 (**2’ax**), 4.4 (**3’**)
**3’**	3.96	12.3 (**2’ax**), 4.4 (**2’eq**), 9.1 (**4’**)
**4'**	3.52	9.1 (**3’**), 9.1 (**5’**)
**5'**	3.84	9.1 (**4’**), (**6’a**)*, 4.5 (**6’b**)
**6'a**	3.37	(**5’**)*, 10.6 (**6’b**)
**6’b**	3.38	4.5 (**5’**), 10.6 (**6’a**)
**O-Bn-CH**_**2**_** (6H)**	4.54–4.96	–
**O-Bn-ar (15H)**	7.26–7.42	–-

*These coupling constants could not be measured. Signal pattern remains partially unclear due to serve signal overlap and higher order effects.

**Table 5:** Chemical carbon shifts for molecule 2C.

^13^C NMR Data for 2C		
Position		^13^C δ (ppm)
**Aglycone Unit**		
**1**	CH_3_	17.7
**2**	CH	72.0
**3**	CH_2_	29.9
**4**	CH_3_	9.7
**Probe Moiety**		
**1'**	Anomeric carbon	93.9
**2'**		35.7
**3’**		77.6
**4'**		78.6
**5'**		71.3
**6'**		69.6
**O-Bn-CH**_**2**_		70.9 72.8 74.5
**O-Bn-ar**	18 aromatic carbons	127.2–128.2

#### ROE diagnostic signals

**Figure 10:**
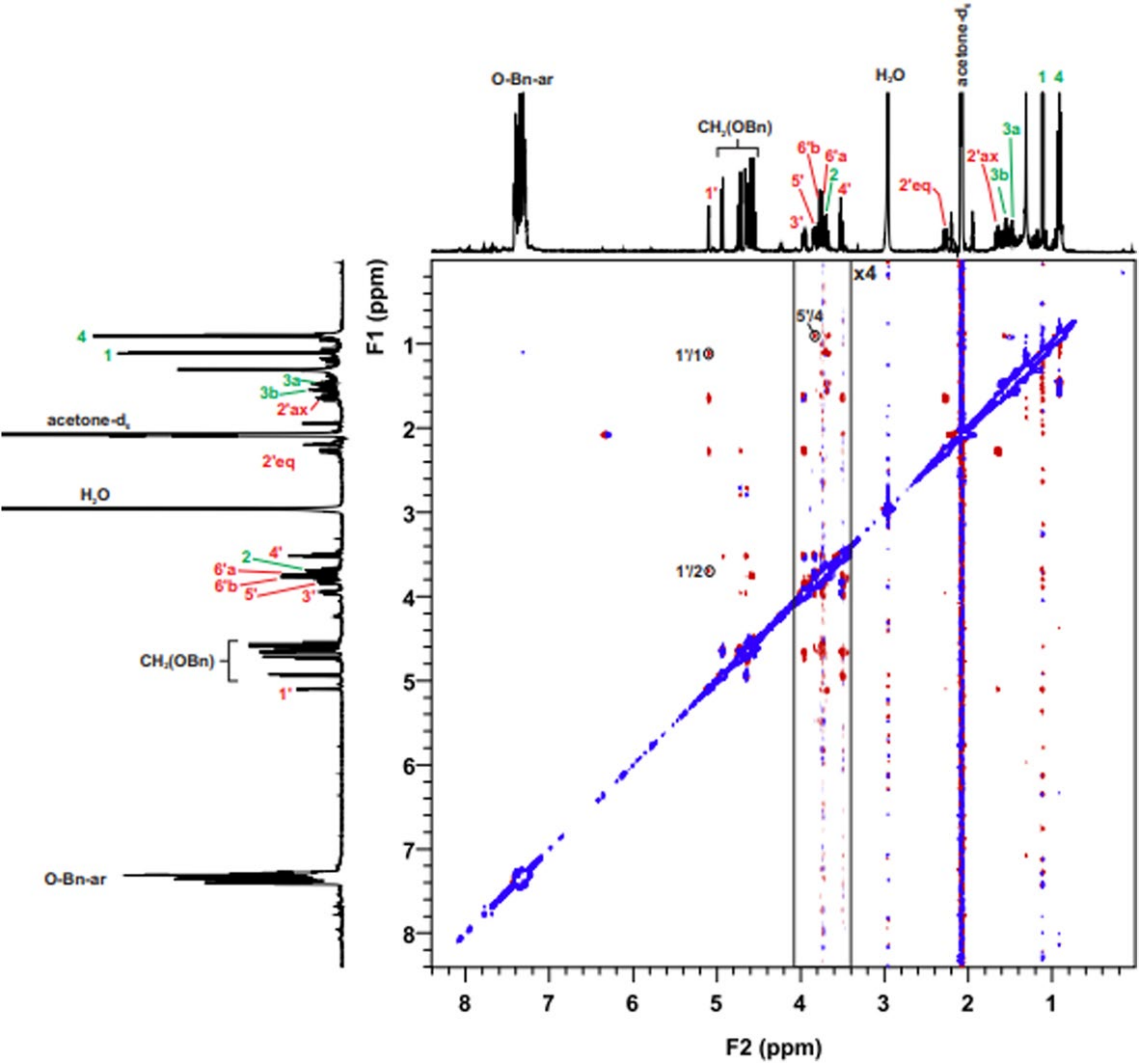
ROESY spectrum of molecule **2C** with all dipole couplings (diagnostic signals are marked in a circle).

In case of the **2C** molecule, the first diagnostic signal was observed—**1'H**/**1H** (Table 6). Other dipole couplings were also noted, including a second diagnostic signal. In this instance, the second relatively weak diagnostic signal ROE—**5'H**/**4H** was also recorded and confirmed the absolute configuration of 2-(S)-butanol (Fig. 6f). From the point of view of these considerations, observation of Overhauser effects for the **2C** molecule was very important for the idea of the proposed method. Presence of the dipole couplings shown indicates the inhibition of rotation around the O-glycosidic bond for such a small aglycone. 2-butanol is one of the smallest optically active secondary alcohol. The results and analysis of NMR experiments confirm previous computer simulations. All of the dipolar couplings for the **2C** molecule can be observed in the ROESY spectrum (Fig. 10 and Fig. S48).

**Table 6:** Diagnostic ROE to proton for **2C.**

2D ROESY NMR Data for **2C**	
Position	Diagnostic ROE to proton
**Probe Moiety**	
**1’**	1, 2
**5’**	4

#### Discussion of NMR results for 1C, 2A and 2B molecules

According to the nd-HSQC spectrum, the obtained compounds **1C**, **2A** and **2B** are alpha anomers. The values of coupling constants ^1^J C-H are presented sequentially: 171.2 Hz, 168.4 Hz, 165.6 Hz, (Fig. S23), (Fig. S31) and (Fig. S39).The NMR spectrum of benzoyl derivatives is less complex than that of benzyl derivatives because it does not contain the –CH_2_- signals (derived from –OBn) which are superimposed on the chemical protons or carbons’ shifts of the probe (Fig. S34), (Fig. S35). On the other hand, these signals are distinguishable because they focus within the certain range on the ppm scale.

#### ROE diagnostic signals

For all molecules **1C**, **2A** and **2B** the ROESY spectrum confirmed the presence of the first diagnostic signal between the probe's **1'H** proton and the aglycone's **1H** proton (Table S9), (Table S12), (Table S15). Additionally, a second diagnostic signal was observed for each case. For **1C**, the next dipolar coupling between the probe and the aglycone is **5'H**/**5exH**. Both interactions allow the determination of the position of the aglycone in space, thus assigning the absolute configuration of 1C-(R*) borneol (Fig. 6c and Fig. S25). For **2A**, three Overhauser effects were observed in the probe—aglycone relationship—**5’H**/**7H**, **5’H**/**8H** and **6’aH**/**8H**. These interdependencies point to the C1-(S*) of menthol oxymethine carbon (Fig. 6d and Fig. S33). Similarly, in the case of the **2B** molecule, the ROESY spectrum proves the occurrence of another dipolar coupling, which is important from the point of view of the present examinations—**5’H**/**7H** which allows to recognize C1- (S *) aglycone (Fig. 6e and Fig. S41).

### Molecular dynamics

In order to assess, whether a glycosidic bond formed by 2-deoxy-d-glucose derivatives in fact exhibits restricted conformational freedom, a set of molecular models of **1A**, **1B**, **1C**, **2A**, **2B** and **2C** were subjected to molecular dynamics studies. Moreover, models of the opposite enantiomers of the studied secondary alcohols with the 2-deoxy-d-glucose-based probes attached, namely **1A’**, **1B’**, **1C’**, **2A’**, **2B’** and **2C’** were also examined by the same computational methods.

**Figure 11:**
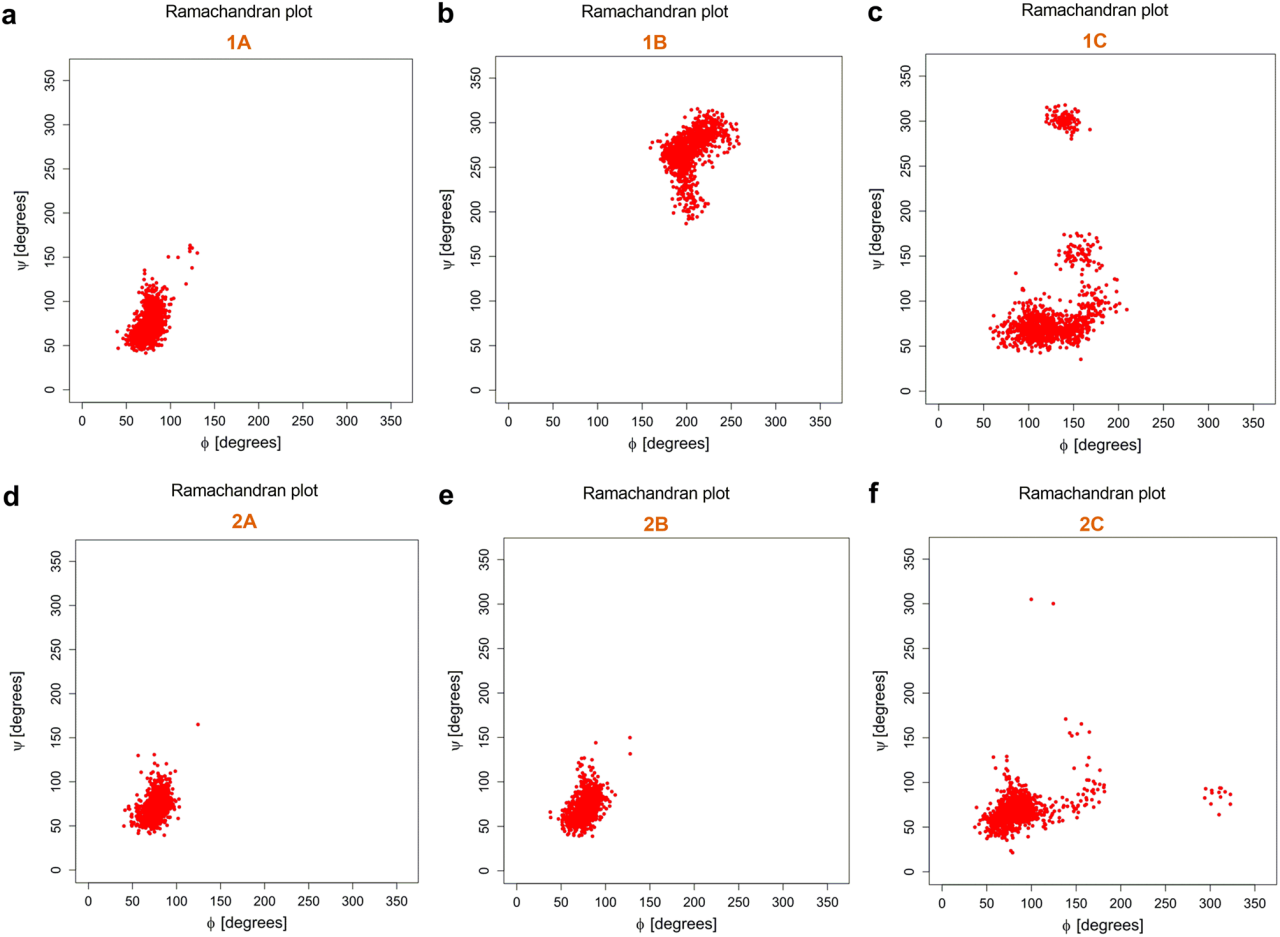
Ramachandran plots of **1A**, **1B**, **1C**, **2A**, **2B** and **2C.**

All 12 studied systems displayed pronounced lack of conformational freedom of the glycosidic linkage, which was evidenced by the Ramachandran plots (Fig. 11a–f and Fig. S49-S60). The studied glycosides almost immediately assumed the geometry, in which the anomeric **1’H** proton of a monosaccharide probe and the oxymethine **1H** proton of an aglycone were in syn-type conformation (Fig. S61). This type of geometry was maintained through the remaining simulation time, as it was associated with the global energetic minima of all studied molecules. Perhaps the most spectacular examples were the **2C** and **2C’** systems, which contained a relatively small aglycone, i.e. 2-butanol moiety. While 2-butanol is one of the smallest chiral secondary alcohols possible, its glycosides still exhibited significantly restricted conformational freedom of glycosidic linkages, as shown at (Fig. 11f and Fig. S60A).This result strongly supports versatility of the presented approach, since the ‘blockade’ of glycosidic bond seems to be an immanent feature of 2-deoxy-d-glucose-based probes, regardless the aglycone’s size and geometry.

**Figure 12:**
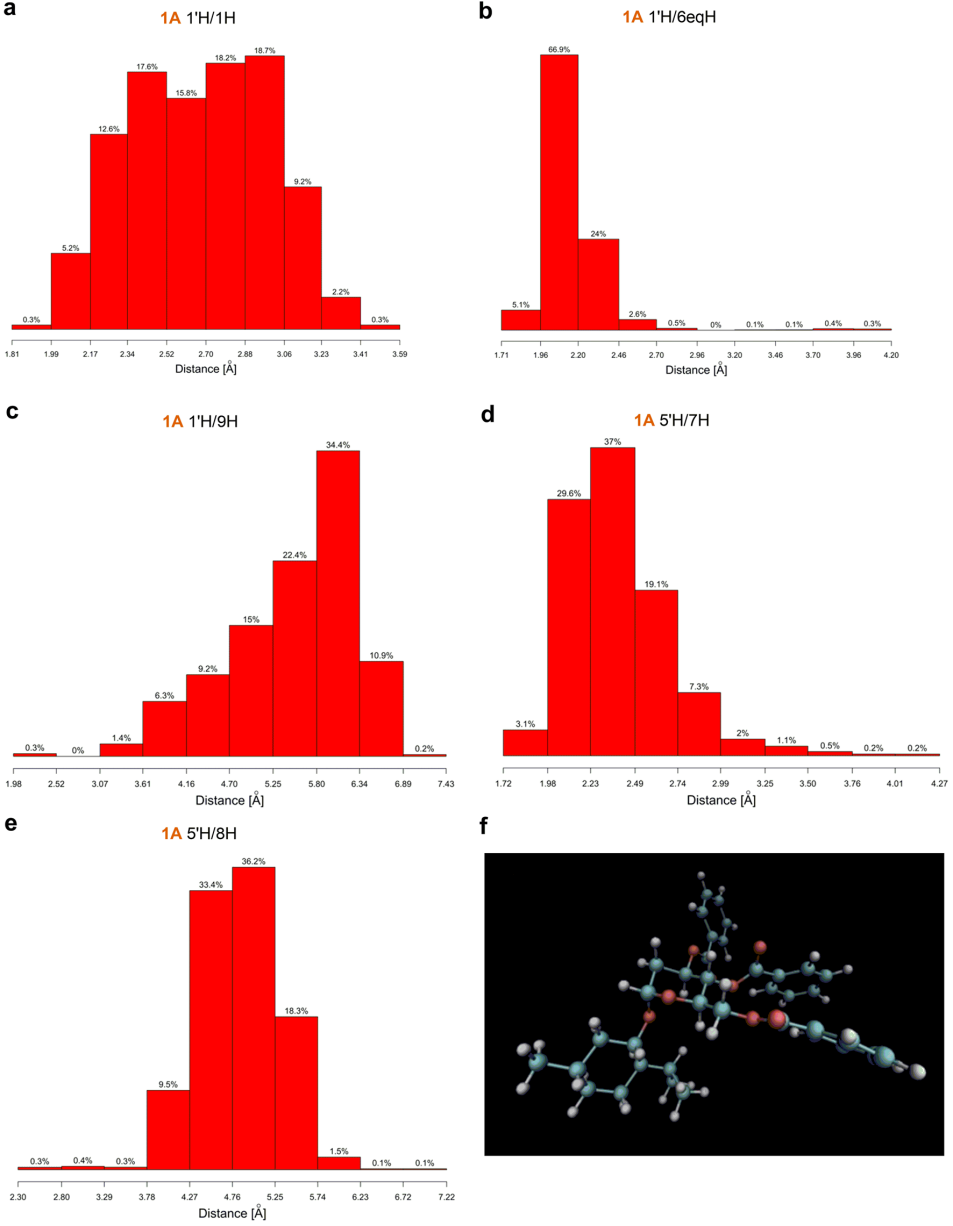
Compound **1A**: histograms of (**a**) **1’H**/**1H** (**b**) **1’H**/**6eqH** (**c**) **1’H**/**9H** (**d**) **5’H**/**7H** (**e) 5’H**/**8H** distances and (**f**) structure.

Lastly, the MD studies have suggested that the **1’H**/**1H** ROEs could—and in fact, were—observed in the ROESY spectra of all examined systems. Nevertheless, since—on the basis of molecular modeling calculations—the **1’H/1H** dipolar couplings were expected in case of all 12 studied glycosides, geometric requirements of the molecules to display all the observed diagnostic dipolar couplings at once, i.e. those involving 1’H, 5’H and 6’H protons, were met only by **1A**, **1C**, **2A**, **2B** and **2C**. For instance, in case of **1A’**, MD simulations have shown that if **1’H/1H** ROE was recorded, ROEs **5’H**/**7H** and **5’H**/**8H** could not have been recorded since average distances between respective protons were too high (Fig. S50). On the other hand, the average interatomic distances in pairs **1’H**/**1H**, **5’H**/**7H** and **5’H**/**8H** extracted from the MD simulation of **1A** were a perfect match for the observed ROESY correlations (Fig. 12). These observations were identical for the rest of the pairs based on aglycone enantiomers (**1C-1C’**, **2A-2A’** and so on, see Fig. S51-S60) and strongly supported the applicability of the presented, 2-deoxy-d-glucose-based approach of elucidation of the absolute configurations of secondary alcohols.

## Experimental

### Materials and methods

Commercially available reagents were purchased from Sigma-Aldrich or Acros. Dichloromethane (DCM) was distilled over phosphorus pentoxide (P_4_O_10_) drying agent and stored over molecular sieves (4 Å). Toluene was distilled from potassium under an argon atmosphere and stored over molecular sieves (4 Å). Thin Layer Chromatography (TLC) analysis was performed on aluminum gelled plates SiliaPlate SILICYCLE UltraPure and visualized with a UV lamp with a wavelength of 254 nm. A cerium-molybdenum developer was used for TLC analysis of the main products. Purification was performed using the BUCHI Pure C-815 flash chromatograph. FlashPure ID cartridges (silica 40 µm irregular) were used during the purification process.

### Synthetic procedures

#### General procedure for synthesis of compound 1A and 1B

##### O-(3,4,6-tri-O-benzoyl-(α,β)-2-deoxy-D-glucopyranosyl) menthol

3,4,6-tri-O-benzoyl-2-deoxy-(α,β)-d-glucose (375 mg, 0.788 mmol) was dissolved in anhydrous DCM (3.6 ml). 4 Å molecular sieves were introduced. Then trichloroacetonitrile (324 µl) was added and the temperature of the mixture was lowered to 0 °C. The reactions were initiated by the addition of a catalytic amount of NaH. After 1 h, the resulting suspension was passed through a thin pad of silica gel system (AcOEt:cyclohexane; 1:7). 0.396 g of crude product (trichloroacetimidate 3,4,6-tri-O-benzoyl-2-deoxy-(α,β)-d-glucose) was obtained in the form of a yellow oil (RF = 0.28). Trichloroacetoimidate 3,4,6-tri-O-benzoyl-2-deoxy-(α,β)-d-glucose (275 mg, 0.443 mmol) was dissolved in anhydrous DCM (3.3 ml). 4 Å molecular sieves were added to the solution, followed by (1S, 2R, 5S)-( +)-menthol (65 mg, 0.415 mmol) and a catalytic amount of TMSOTf. Reactions were carried out at room temperature for 24 h. Then a few drops of Et_3_N (30 µl) were added. The obtained mixture was concentrated and purified by flash chromatography (AcOEt:hexanes system 1% Et_3_N; 1:15). 122 mg of the product was obtained in the form of a white solid (50:50%; α:β), (R_F_ = 0.33, for AcOEt:hexanes system 1% Et_3_N; 1:15). Then, the obtained mixture was separated into alpha and beta anomer (more details in the Supporting Information pages S6-S7).

#### General procedure for synthesis of compound 1C

##### O-(3,4,6-tri-O-benzoyl-(α,β)-2-deoxy-D-glucopyranosyl) borneol

3,4,6-tri-O-benzoyl-2-deoxy-(α,β)-d-glucose (247 mg, 0.519 mmol) was dissolved in anhydrous DCM (2.4 ml). 4 Å molecular sieves were introduced. Then, trichloroacetonitrile (214 µl) was added in room temperature. The reactions were initiated by the addition of catalytic 0.172 g of crude product (trichloroacetimidate 3,4,6-tri-O-benzoyl-2-deoxy-(α,β)-d-glucose) was obtained in the form of a yellow oil (RF = 0.28). Trichloroacetoimidate 3,4,6-tri-O-benzoyl-2-deoxy-(α,β)-d-glucose (172 mg, 0.277 mmol) was dissolved in anhydrous DCM (1.8 ml). 4 Å molecular sieves were added to the solution, followed by (-)-borneol (39 mg, 0.252 mmol) and a catalytic amount of TMSOTf. Reactions were carried out at room temperature for 24 h. Then a few drops of Et_3_N (20 µl) were added. The obtained mixture was concentrated and purified by flashchromatography (AcOEt:hexanes system 1% Et_3_N; 1:3). 28 mg of the product was obtained in the form of a white solid (90:10%; α:β) (R_F_ = 0.31, for A:H system 1% Et_3_N; 1:3). amount of NaH. After 1 h, the resulting suspension was passed through a thin pad of silica gel system (AcOEt:cyclohexane; 1:7).

#### General procedure for synthesis of compound 2A, 2B and 2C

##### O-(3,4,6-tri-O-benzyl-(α,β)-2-deoxy-D-glucopyranosyl) secondary alkohols

3,4,6-tri-O-benzyl-2-deoxy-(α,β)-d-glucose (86 mg, 0.2 mmol) was dissolved in anhydrous DCM (3 ml). 4 Å molecular sieves were introduced. Then, trichloroacetonitrile (200 µl, 2 mmol 10 eq) was added at room temperature. The reactions were initiated by the addition of a catalytic amount of NaH in oil. After 2 h, the resulting suspension was passed through a thin pad of silica gel system (AcOEt:hexanes; 1:3 with 1% Et_3_N). The crude product (trichloroacetimidate 3,4,6-tri-O-benzyl-2-deoxy-(α,β)-d-glucose) was obtained in the form of a yellow oil and immediately used for the next step). Crude trichloroacetoimidate 3,4,6-tri-O benzyl-2-deoxy-(α,β)-d-glucose was dissolved in anhydrous DCM (3 ml). 4 Å molecular sieves were added to the solution, followed by (0.6 mmol) of appropriate alcohol (menthol or (S)-2-butanol) and a catalytic amount (15 µl) of TMSOTf. Reactions were carried out at room temperature for 24 h. Then, (300 µl) of Et_3_N was added. The obtained mixture was concentrated and purified by flash chromatography (AcOEt:hexanes; 1:10, with 1% Et_3_N) yielding suitable secondary alkyl 3,4,6-tri-O-benzyl-(α,β)-2-deoxy-D-glucopyranosides in the form of a white solids.

#### General information of NMR experiments

^1^H and ^13^C NMR spectra were recorded on a Varian INOVA 500 spectrometer at 500 and at 125 MHz, respectively. ^1^H NMR spectrum were collected with standard parameters (at ambient temperature, 45° pulse length, 2 s acquisition time and delay time 1 s) in acetone-d_6_ or benzene-d_6_ solution. Chemical shifts are reported in *δ* (ppm) units using ^1^H (residual) from acetone-d6 (2.05 ppm) or benzene-d_6_ (7.16 ppm) as internal standard. ^13^C NMR spectrum were collected with standard parameters (at ambient temperature, 45° pulse length, 1 s acquisition time and 1 s delay time) in acetone-d6 or benzene-d6 solution.

Two-dimensional NMR spectra were recorded at ambient temperature in acetone-d_6_ or benzene-d_6_ solution. The set of 2D spectra for each compound includes: gDQCOSY, zTOCSY, ROESYAD, gHSQCAD, *non*-*decoupled* gHSQCAD, gHMBCAD experiments. More experimental details are included in the Supporting Information file (pages S4-S64).

#### General information of molecular modelling

Parameters for the molecular models of **1A**, **1A’**, **1B**, **1B’**, **1C**, **1C’**, **2A**, **2A’**, **2B**, **2B’**, **2C** and **2C’** were taken from CHARMM carbohydrate-specific force field^20^. Partial atomic charges were recalculated using GAUSSIAN09 software^21^ at MP2/6-31G* level of theory. All the studied compounds were explicitly solvated in acetone or benzene cubic boxes (circa 600 solvent molecules), accordingly to the solvent used in NMR studies. Acetone and benzene parameters were taken from CHARMM36 Generalized Force Field^22^. After initial, 100-ns long equilibration, all 12 systems were subjects to 200-ns long MD runs. All the simulations were carried out using GROMACS 2020.4 software^23^ using leapfrog scheme with a time step of 2 fs. The particle mesh Ewald technique with a cutoff of 1 nm and a grid spacing of approx. 0.1 nm was employed to evaluate the electrostatic forces. The van der Waals interactions were calculated using a Lennard–Jones potential with a cutoff of 1 nm. The simulation was conducted at a constant temperature of 298 K and a constant pressure of 1 bar using Parrinello-Rahman barostat with relaxation times of 0.1 ps and 0.5 ps, respectively. All of the covalent bonds’ lengths were constrained using the P-LINCS and SETTLE algorithms. All the Ramachandran plots and histograms were prepared using R programming language, v 4.1.2^24^.

## Conclusions

In this contribution, we have evidenced the applicability of 2-deoxy-d-glucose-based probes for elucidation of the stereochemistry of chiral secondary alcohols. The foundation of a probe, namely 2-deoxy-d-glucose, is relatively inexpensive and commonly available; the syntheses of its O-benzyl and O-benzoyl derivatives are relatively straightforward. The glycosylation of an secondary alcohol is also fairly uncomplicated, as the studied probes almost selectively yield α-glycosides, which are far more useful for stereochemical studies. The absolute configuration of an oxymethine carbon atom of a studied alcohol may be assigned via set of standard 2D NMR experiments. The results are unambiguous due to the proven lack of conformational freedom of probe-aglycone glycosidic linkage, as evidenced by both NMR and molecular modeling calculations. Our studies suggest that 3,4,6-tri-O-benzoyl-2-deoxy-d-glucose might a better probe in general since the proton resonances of monosaccharide’s protective groups do not superimpose with 2-deoxyglucose’s signals.

Possibly the most important observation, gained from both NMR and MD studies, was the fact that the 2-deoxy-d-glucose-based chiral probe was proven to be successful in case of 2-butanol. This molecule is one of the smallest and lesser sterically hindered, chiral secondary alcohols possible. While both ROESY spectrum and MD calculations have unambiguously proven that in case of **2C** system the conformational freedom of its glycosidic bond was significantly restricted, one could reasonably conclude that any secondary alcohol, ‘bulkier’ than 2-butanol, would also be a viable target for stereochemical studies incorporating the discussed probes. This assumption was supported by studies on menthol and borneol models presented herein; additional examples, considering more complex aglycones, are to be examined in the near future. Nevertheless, one should acknowledge that the method proposed in this contribution was inspired by natural compounds, i.e. heptaene macrolide antifungal antibiotics, which contain an enormous, secondary alcohol aglycone attached to naturally occurring chiral probe—in most cases, the mycosamine moiety^25,26^. Stereochemistry of many of these compounds was elucidated basing on the same concepts as discussed herein^27–30^. Therefore, while incorporating the 2-deoxy-d-glucose’s derivatives as the chiral probes, the sterical hindrance of a studied secondary alcohol could possibly be an issue only at the stage of the probe’s attachment, potentially lowering the total yield of the organic syntheses.

## Supplementary Information

Supplementary Legends.

Supplementary Information.

## Data Availability

The datasets presented in the current study are available from the corresponding author on reasonable request.
