# Supplementary material for: Application of the 2-deoxyglucose scaffold as a new chiral probe for elucidation of the absolute configuration of secondary alcohols
Source: Sci Rep. 2022 Oct 7;12:16838. doi: 10.1038/s41598-022-21174-8 (PMC9547072; doi:10.1038/s41598-022-21174-8)
Supplement: Supplementary file 1 — Supplementary Legends. [file 41598_2022_21174_MOESM1_ESM.docx]

# Additional information

**Electronic Supplementary Information** (ESI) available: 1D and 2D NMR spectra (images) of studied glycosides, data on chemical shifts, coupling constants and Overhauser effects (tables), MD simulation analysis and histograms of **1A**, **1B**, **1C** and **2A**, **2B**, **2C**.
