# Supplementary material for: Application of the 2-deoxyglucose scaffold as a new chiral probe for elucidation of the absolute configuration of secondary alcohols
Source: Sci Rep. 2022 Oct 7;12:16838. doi: 10.1038/s41598-022-21174-8 (PMC9547072; doi:10.1038/s41598-022-21174-8)
Supplement: Supplementary file 2 — Supplementary Information. [file 41598_2022_21174_MOESM2_ESM.pdf]

ELECTRONIC SUPPLEMENTARY INFORMATION

Application of the 2-deoxyglucose scaffold as a new chiral probe for elucidation of the absolute configuration of secondary alcohols

Alicja Trocka[1,*], Katarzyna Szwarc-Karabyka[2], Sławomir Makowiec[1], Tomasz Laskowski[3]

[1] Department of Organic Chemistry, Faculty of Chemistry, Gdańsk University of Technology, Narutowicza Street 11/12, 80-233 Gdańsk, Poland; mak@pg.edu.pl (S.M.)

[2] Nuclear Magnetic Resonance Laboratory, Faculty of Chemistry, Gdańsk University of Technology, Narutowicza Street 11/12, 80-233 Gdańsk, Poland; katszwar@pg.edu.pl (K.S.-K.)

[3] Department of Pharmaceutical Technology and Biochemistry, Faculty of Chemistry, Gdańsk University of Technology, Narutowicza Street 11/12, 80-233 Gdańsk, Poland; tomasz.laskowski@pg.edu.pl (T.L.)

***Correspondence:** tel: +48 509 971 512 e-mail address: alicja.trocka@pg.edu.pl

TABLE OF CONTENTS

1.	List of figures	S2
2.	List of tables	S3
3.	Experimental section	S4
3.1.	Synthesis of benzoyl derivatives	S4
3.2.	Synthesis of benzyl derivatives	S9
4.	Structures of chiral probes	S13
5.	NMR data assignments of 1A – 2C	S14
5.1	Spectral sets for chiral probes	S14
5.2	NMR experimental dietails	S62
6.	Molceular modeling	S65
7.	References	S78

1. List of figures

**Figure.1.**	^{1}H NMR spectrum for **1A**	S14
**Figure.2**	^{13}C NMR spectrum for **1A**	S15
**Figure.3.**	DQF-COSY spectrum for **1A**	S16
**Figure.4.**	TOCSY spectrum for **1A**	S17
**Figure.5.**	HSQC spectrum for **1A**	S18
**Figure.6.**	nd-HSQC spectrum for **1A**	S19
**Figure.7.**	HMBC spectrum for **1A**	S20
**Figure.8**	H2BC spectrum for **1A**	S21
**Figure.9.**	ROESY spectrum for **1A**	S22
**Figure.10.**	^{1}H NMR spectrum for **1B**	S23
**Figure.11.**	^{13}C NMR spectrum for **1B**	S24
**Figure.12.**	DQF-COSY spectrum for **1B**	S25
**Figure.13.**	TOCSY spectrum for **1B**	S26
**Figure.14**	HSQC spectrum for **1B**	S27
**Figure.15.**	nd-HSQC spectrum for **1B**	S28
**Figure.16.**	HMBC spectrum for **1B**	S29
**Figure.17.**	ROESY spectrum for **1B**	S30
**Figure.18.**	^{1}H NMR spectrum for **1C**	S31
**Figure.19.**	^{13}C NMR spectrum for **1C**	S32
**Figure.20**	DQF-COSY spectrum for **1C**	S33
**Figure.21.**	TOCSY spectrum for **1C**	S34
**Figure.22.**	HSQC spectrum for **1C**	S35
**Figure.23.**	nd-HSQC spectrum for **1C**	S36
**Figure.24.**	HMBC spectrum for **1C**	S37
**Figure.25.**	ROESY spectrum for **1C**	S38
**Figure.26**	^{1}H NMR spectrum for **2A**	S39
**Figure.27.**	^{13}C NMR spectrum for **2A**	S40
**Figure.28.**	DQF-COSY spectrum for **2A**	S41
**Figure.29.**	TOCSY spectrum for **2A**	S42
**Figure.30.**	HSQC spectrum for **2A**	S43
**Figure.31.**	nd-HSQC spectrum for **2A**	S44
**Figure.32**	HMBC spectrum for **2A**	S45
**Figure.33.**	ROESY spectrum for **2A**	S46
**Figure.34.**	^{1}H NMR spectrum for **2B**	S47
**Figure.35.**	^{13}C NMR spectrum for **2B**	S48
**Figure.36.**	DQF-COSY spectrum for **2B**	S49
**Figure.37.**	TOCSY spectrum for **2B**	S50
**Figure.38.**	HSQC spectrum for **2B**	S51
**Figure.39.**	nd-HSQC spectrum for **2B**	S52
**Figure.40.**	HMBC spectrum for **2B**	S53
**Figure.41.**	ROESY spectrum for **2B**	S54
**Figure.42.**	^{1}H NMR spectrum for **2C**	S55
**Figure.43.**	^{13}C NMR spectrum for **2C**	S56
**Figure.44.**	DQF-COSY spectrum for **2C**	S57

**Figure.45.**	TOCSY spectrum for **2C**	S58
**Figure.46.**	HSQC spectrum for **2C**	S59
**Figure.47.**	nd-HSQC spectrum for **2C**	S60
**Figure.48.**	ROESY spectrum for **2C**	S61
**Figure.49.**	Compound **1A'**: histograms of H1'/H1, H1'/H6eq, H1'/H9, H5'/H7 and H5'/H8 distances	S65
**Figure.50.**	Compound **1A'**: histograms of H1'/H1, H1'/H6eq, H1'/H9, H5'/H7 and H5'/H8 distances	S66
**Figure.51.**	Compound **1B**: histograms of H1'/H1, H1'/H6eq, H1'/H7 and H1'/H9 distances	S67
**Figure.52.**	Compound **1B'**: histograms of H1'/H1, H1'/H6eq, H1'/H7 and H1'/H9 distances	S68
**Figure.53.**	Compound **1C**: histograms of H1'/H1, H1'/H10 and H5'/H5ex distances	S69
**Figure.54.**	Compound **1C'**: histograms of H1'/H1, H1'/H10 and H5'/H5ex distances	S70
**Figure.55.**	Compound **2A**: histograms of H1'/H1, H1'/H6eq, H5'/H7, H5'/H8 and H6'a/H8 distances	S71
**Figure.56.**	Compound **2A'**: histograms of H1'/H1, H1'/H6eq, H5'/H7, H5'/H8 and H6'a/H8 distances	S72
**Figure.57.**	Compound **2B**: histograms of H1'/H1, H1'/H6eq and H5'/H7	S73
**Figure.58.**	Compound **2B'**: histograms of H1'/H1, H1'/H6eq and H5'/H7	S74
**Figure.59.**	Compound **2C**: histograms of H1'/H1, H1'/H2 and H5'/H4	S75
**Figure.60.**	Compound **2C'**: histograms of H1'/H1, H1'/H2 and H5'/H4	S76
**Figure.61.**	Definition of double-walled angles Φ and Ψ chiral probe - aglycone	S77

2. List of tables

**Table.1.**	Chemical shifts and coupling constants for molecule **1A** (^{1}H NMR)	S14
**Table.2**	Chemical shifts for molecule **1A** (^{13}C NMR)	S15
**Table.3.**	Diagnostic ROE to proton for **1A**	S22
**Table.4.**	Chemical shifts and coupling constants for molecule **1B** (^{1}H NMR)	S23
**Table.5.**	Chemical shifts for molecule **1B** (^{13}C NMR)	S24
**Table.6.**	Diagnostic ROE to proton for **1B**	S30
**Table.7.**	Chemical shifts and coupling constants for molecule **1C** (^{1}H NMR)	S31
**Table.8.**	Chemical shifts for molecule **1C** (^{13}C NMR)	S32
**Table.9.**	Diagnostic ROE to proton for **1C**	S38
**Table.10**	Chemical shifts and coupling constants for molecule **2A** (^{1}H NMR)	S39
**Table.11.**	Chemical shifts for molecule **2A** (^{13}C NMR)	S40
**Table.12.**	Diagnostic ROE to proton for **2A**	S46
**Table.13.**	Chemical shifts and coupling constants for molecule **2B** (^{1}H NMR)	S47
**Table.14.**	Chemical shifts for molecule **2B** (^{13}C NMR)	S48
**Table.15.**	Diagnostic ROE to proton for **2B**	S54
**Table.16.**	Chemical shifts and coupling constants for molecule **2C** (^{1}H NMR)	S55
**Table.17.**	Chemical shifts for molecule **2C** (^{13}C NMR)	S56
**Table.18.**	Diagnostic ROE to proton for **2C**	S61

3. Experimental section

3.1. Synthesis of benzoyl derivatives

1,3,4,6-tetra-O-benzoyl-2-deoxy-(α,β)-D-glucopyranoside

A mixture of 2-deoxy-(α,β)-D-glucose (1 g, 6.09 mmol), benzoyl chloride (4.25 ml) and DMAP (33.4 mg, 0.27 mmol) in anhydrous pyridine (40 ml) was stirred at room temperature for 72 h. The contents of flask were evaporated with toluene, then the mixture was diluted with water and extracted with DCM (90 ml - 2 x 45 ml). The organic layer was washed with a saturated $CuSO_4$ solution and dried with $MgSO_4$. The crude product was purified by flash chromatography (ethyl acetate:hexane system; gradient 1:15 -> 1:3), 2.41 g (68%) of the product was obtained in the form of a white solid (5%:95% α:β); (R_F = 0.26 and R_F = 0.33, for A:H system: 1:6).

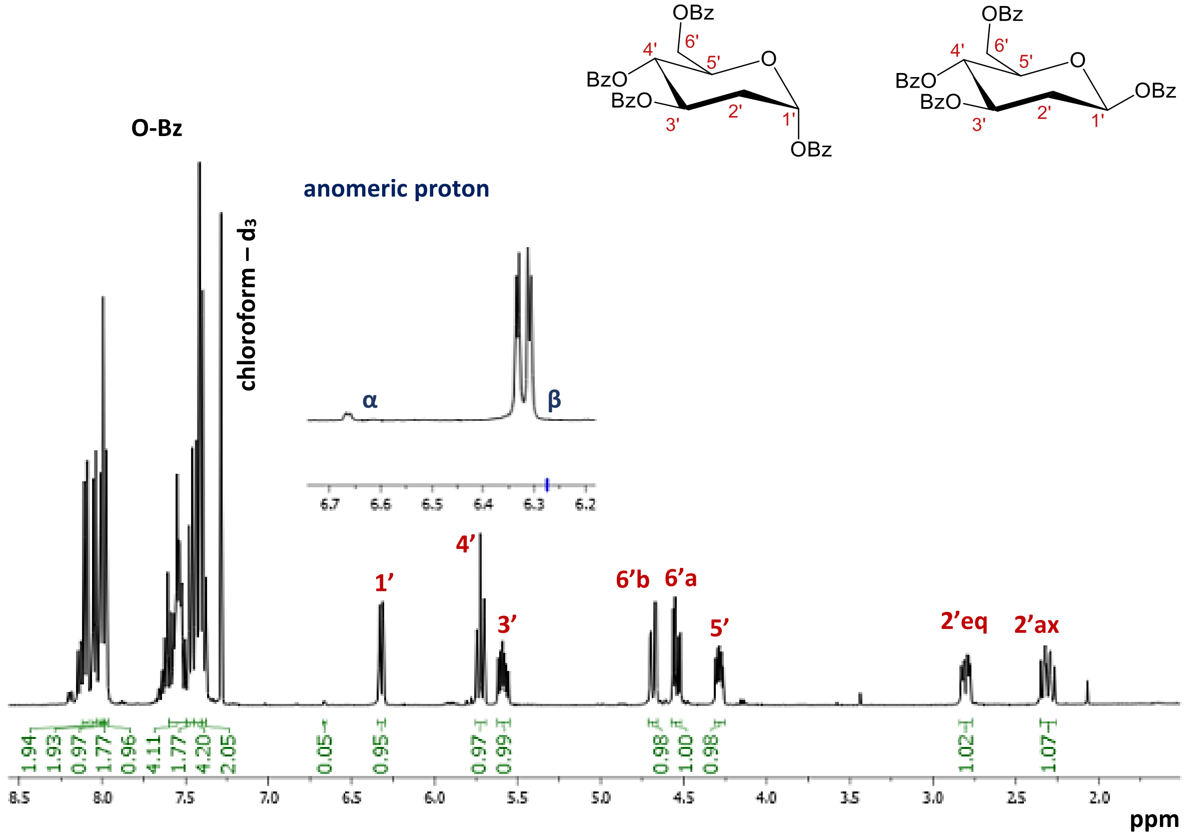

^{1}H NMR (500 MHz, $CDCl_3$) δ = 8.22 – 7.36 (m, 20H, **O-Bz**), 6.32 (dd, 1H, J^3=3.0, 12.0 Hz, **1’**), 5.72 (t, 1H, ^3J = 11.5 Hz, **4’**), 5.63 – 5.55 (m, 1H, **3’**), 4.68 (dd, 1H, J^3=4.0, 15.5 Hz, **6’b**), 4.54 (dd, 1H, J^3=6.5, 15.5 Hz, **6’a**), 4.31 – 4.25 (m, 1H, **5’**), 2.83 – 2.77 (m, 1H, **2’eq**), 2.36 – 2.26 (m, 1H, **2’ax**)

3,4,6-tri-O-benzoyl-2-deoxy-(α,β)-D-glucose

HBr / HOAc (45%, 0.3 ml) under argon was added to a solution of 1,3,4,6-tetra-O-benzoyl-(α, β)-2-deoxy-D-glucose (0.250 g, 0.431 mmol) in dry DCM (1.3 ml). The reaction was stirred at room temperature for 2.5 h. Then the solution was washed with saturated $NaHCO_3$ solution and brine. The residue was dissolved in acetone (2.3 ml), H_2O (0.09 ml), and DCM (0.2 ml). Then Ag_2CO_3 (0.237 g, 0.86 mmol) was added portionwise, the reaction was stirred for 1 hour at room temperature. The mixture was successively filtered through a bed of celite. The filtrate was concentrated under reduced pressure and the residue was purified by flash column chromatography (ethyl acetate:hexane system; 3:7 -> 2:3), 0.172 g (86%) of the product was obtained in the form of a white foam (80%:20% α:β); (R_F = 0.31 and R_F = 0.24, for A:H system: 3:7).

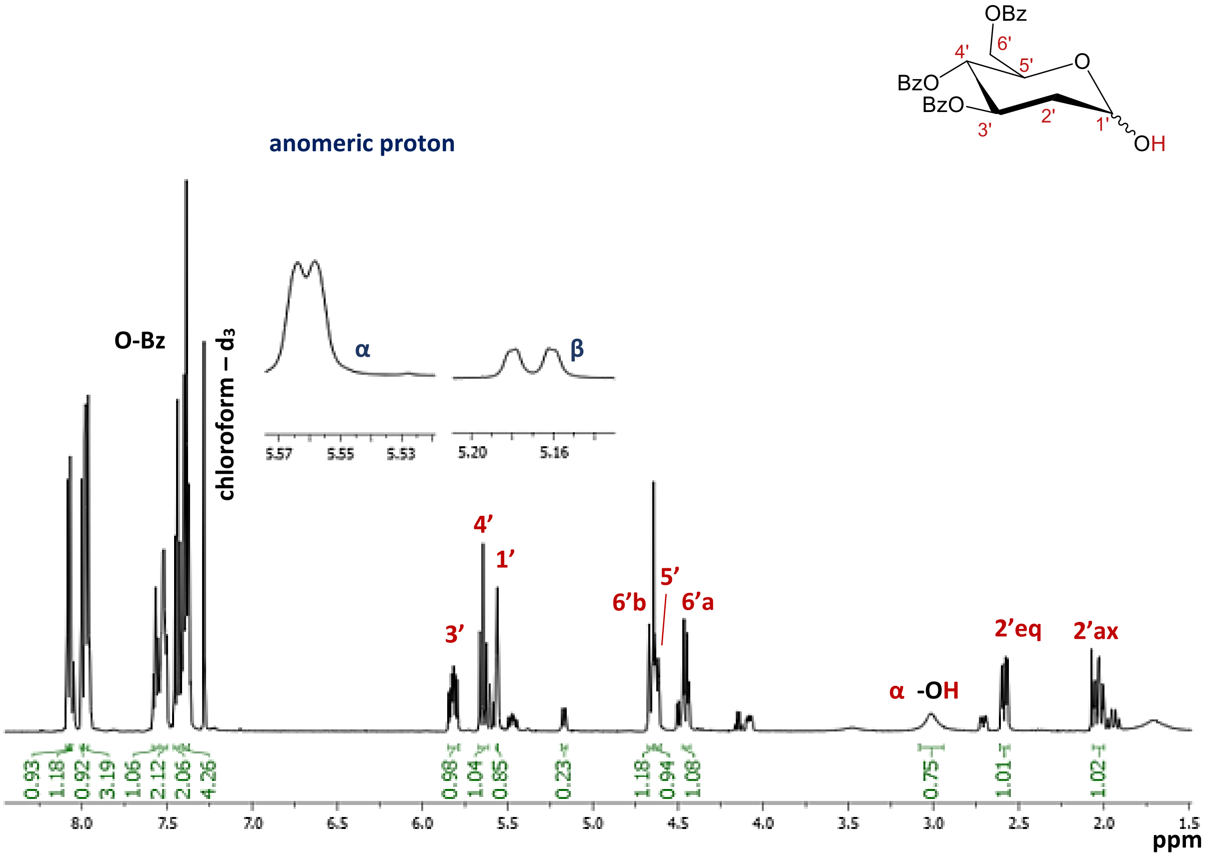

^{1}H NMR (500 MHz, $CDCl_3$) δ = 8.10 – 7.35 (m, 15H, **O-Bz**), 5.85 – 5.79 (m, 1H, **3'**), 5.65 (t, 1H, ^{3}J = 9.5 Hz, **4'**), 5.56 (dd, 1H, J^3=~0.0, 3.0 Hz, **1'**), 4.66 (m, 1H, **6'b**), 4.65 – 4.60 (m, 1H, **5'**), 4.45 (dd, 1H, J^3=4.0, 12.0 Hz, **6'a**), 3.01 (s, 1H, -O**H**), 2.60 – 2.55 (m, 1H, **2'eq**), 2.06 – 1.99 (m, 1H, **2'ax**)

O-(3,4,6-tri-O-benzoyl-(α,β)-2-deoxy-D-glucopyranosyl) menthol (1A, 1B)

3,4,6-tri-O-benzoyl-2-deoxy-(α,β)-D-glucose (375 mg, 0.788 mmol) was dissolved in anhydrous DCM (3.6 ml). 4 Å molecular sieves were introduced. Then trichloroacetonitrile (µl) was added and the temperature of the mixture was lowered to 0°C. The reactions were initiated by the addition of a catalytic amount of NaH. After 1 h, the resulting suspension was passed through a thin pad of silica gel system (ethyl acetate: cyclohexane; 1:7). 0.396 g of crude product (trichloroacetimidate 3,4,6-tri-O-benzoyl-2-deoxy-(α,β)-D-glucose) was obtained in the form of a yellow oil (R_F = 0.28). Trichloroacetoimidate 3,4,6-tri-O-benzoyl-2-deoxy-(α,β)-D-glucose (275 mg, 0.443 mmol) was dissolved in anhydrous DCM (3.3 ml). 4 Å molecular sieves were added to the solution, followed by (1S, 2R, 5S)-(+)-menthol (65 mg, 0.415 mmol) and a catalytic amount of TMSOTf. Reactions were carried out at room temperature for 24 h. Then a few drops of Et_3N (30 µl) were added. The obtained mixture was concentrated and purified by flash chromatography (ethyl acetate:hexane system 1% Et_3N; 1:15). 122 mg (44%) of the product was obtained in the form of a white solid (50%:50% α:β) (R_F = 0.33, for A:H system 1% Et_3N; 1:15).

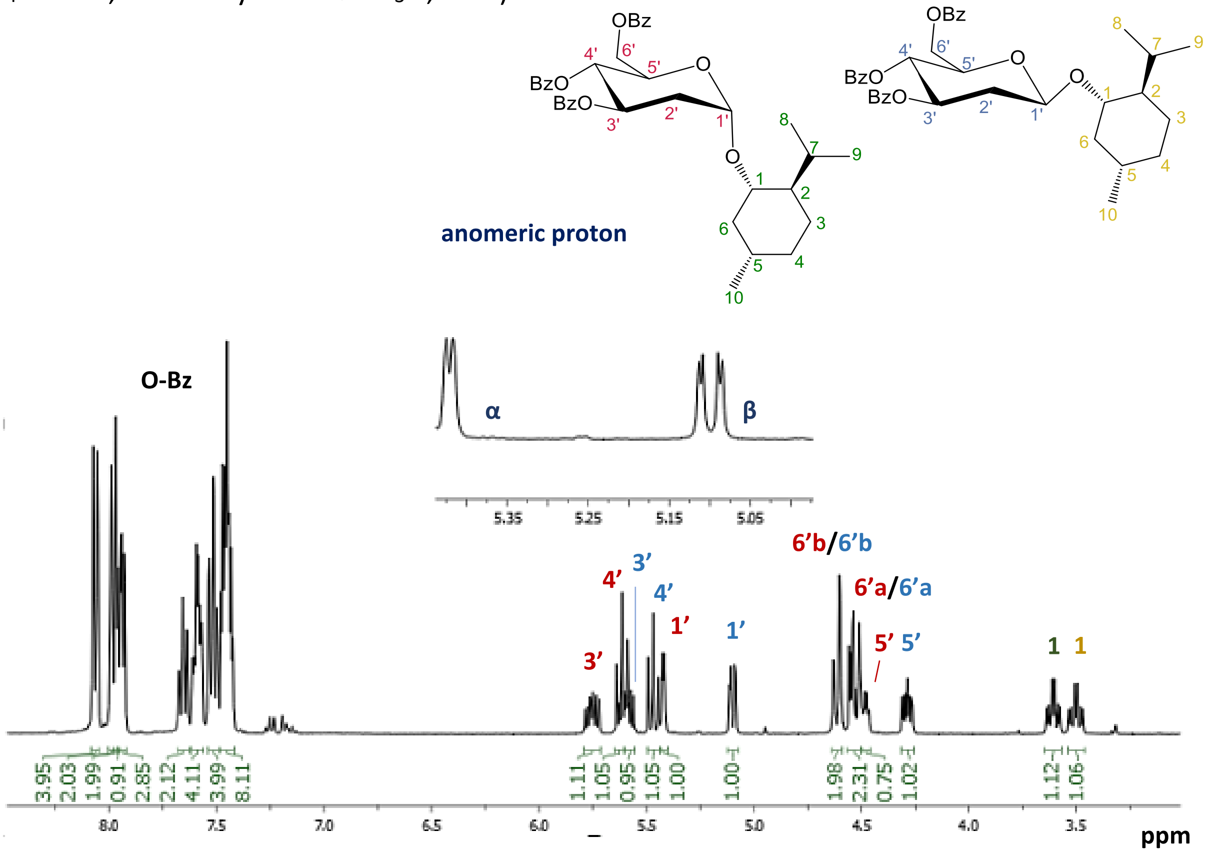

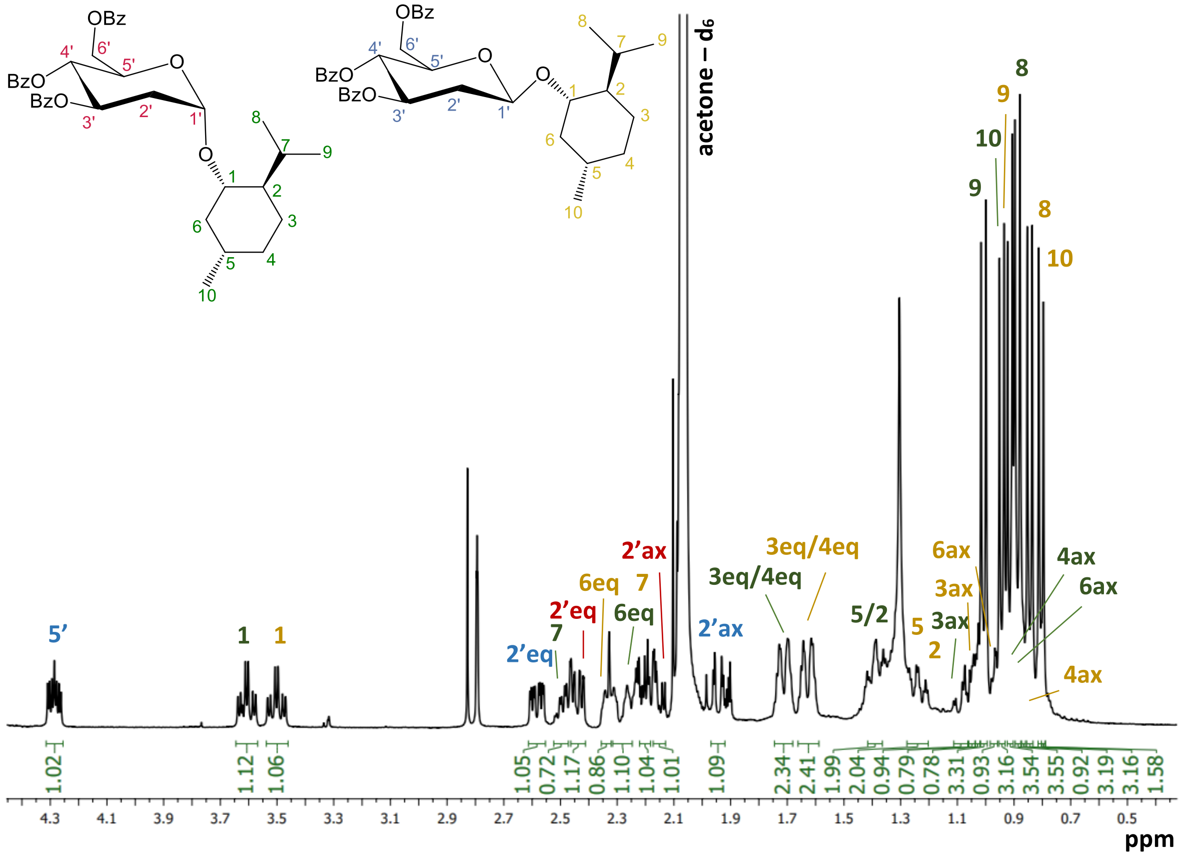

The obtained mixture of anomers was separated by column chromatography (100% toluene system), 27 mg of (+)-1-(3,4,6-tri-O-benzoyl-(α)-2-deoxy-D-glucopyranosyloxy) menthol was obtained in the form of white solid (R_F = 0.35) and 4 mg (+)-1- (3,4,6-tri-O-benzoyl-(β)-2-deoxy-D-glucopyranosyloxy) menthol as a white solid (R_F = 0.26).

^{1}H NMR (500 MHz, acetone-d$_6$) δ = 8.10 – 7.40 (m, 30H, **O-Bz**), 5.80 – 5.72 (m, 1H, **3'**), 5.61 (t, 1H, ^{3}J = 12.5, **4'**), 5.62 – 5.56 (m, 1H, **3'**), 5.47 (t, 1H, ^{3}J = 12.0, **4'**), 5.42 (dd, 1H, J^3=~0.0, 3.5 Hz, **1'**), 5.10 (dd, 1H, J^3=2.0, 11.5 Hz, **1'**), 4.63 – 4.59 (m, 2H, **6'b**/**6'b**), 4.57 – 4.50 (m, 2H, **6'a**/**6'a**), 4.50 – 4.46 (m, 1H, **5'**), 4.31 – 4.26 (m, 1H, **5'**), 3.65 – 3.57 (m, 1H, **1**), 3.54 – 3.47 (m, 1H, **1**), 2.61 – 2.55 (m, 1H, **2'eq**), 2.52 – 2.48 (m, 1H, **7**), 2.46 – 2.41 (m, 1H, **2'eq**), 2.35 – 2.32 (m, 1H, **6eq**), 2.31 – 2.25 (m, 1H, **6eq**), 2.21 – 2.19 (m, 1H, **7**), 2.17 – 2.13 (m, 1H, **2'ax**), 1.96 – 2.93 (m, 1H, **2'ax**), 1.74 – 1.68 (m, 2H, **3eq**/**4eq**), 1.66 – 1.59 (m, 2H, **3eq**/**4eq**), 1.41 – 1.37 (m, 2H, **5**/**2**), 1.28 – 1.21(m, 2H, **5**/**2**), 1.11 – 1.07 (m, 1H, **3ax**), 1.06 – 1.04 (m, 1H, **3ax**), 1.04 – 1.03 (m, 1H, **6ax**), 1.02 – 0.99 (m, 1H, **4ax**), 1.00 (d, 3H, J^3=8.5 Hz, **9**), 0.98– 0.96 (m, 1H, **6ax**), 0.95 (d, 3H, J^3=8.5 Hz, **10**), 0.92 (d, 3H, J^3=8.5 Hz, **9**), 0.89 (d, 3H, J^3=8.5 Hz, **8**), 0.88 – 0.86 (m, 1H, **4ax**), 0.84 (d, 3H, J^3=8.5 Hz, **8**), 0.80 (d, 3H, J^3=8.5 Hz, **10**)

1D and 2D NMR spectra sets for the major products are presented in **Section 5.**

O-(3,4,6-tri-O-benzoyl-(α,β)-2-deoxy-D-glucopyranosyl) borneol (1C)

3,4,6-tri-O-benzoyl-2-deoxy-(α,β)-D-glucose (247 mg, 0.519 mmol) was dissolved in anhydrous DCM (2.4 ml). 4 Å molecular sieves were introduced. Then trichloroacetonitrile (214 µl) was added in room temperature. The reactions were initiated by the addition of a catalytic amount of NaH. After 1 h, the resulting suspension was passed through a thin pad of silica gel system (ethyl acetate: cyclohexane; 1:7). 0.172 g of crude product (trichloroacetimidate 3,4,6-tri-O-benzoyl-2-deoxy-(α,β)-D-glucose) was obtained in the form of a yellow oil (R_F = 0.28). Trichloroacetoimidate 3,4,6-tri-O-benzoyl-2-deoxy-(α,β)-D-glucose (172 mg, 0.277 mmol) was dissolved in anhydrous DCM (1.8 ml). 4 Å molecular sieves were added to the solution, followed by (-)-borneol (39 mg, 0.252 mmol) and a catalytic amount of TMSOTf. Reactions were carried out at room temperature for 24 h. Then a few drops of Et_3N (20 µl) were added. The obtained mixture was concentrated and purified by flash chromatography (ethyl acetate:hexane system 1% Et_3N; 1:3). 28 mg of the product was obtained in the form of a white solid (90%:10% α:β) (R_F = 0.31, for A:H system 1% Et_3N; 1:3).

1D and 2D NMR spectra sets for the major products are presented in **Section 5.**

3.2. Synthesis of benzyl derivatives

1-O-Methyl-2-Deoxy-(α,β)-D-glucopyranose

was prepared according to literature procedure [1], ^{1}H NMR spectrum of prepared compound was in agreement with reference data.

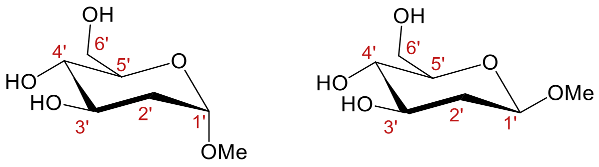

1-O-Methyl-2-deoxy-3,4,6-tri-O-benzyl-(α,β)-D-glucopyranose

1-O-Methyl-2-Deoxy-(α/β)-D-glucopyranose (890 mg, 5 mmol) was dissolved in dry DMF (10 ml), benzyl bromide (2.74 g, 16 mmol) was added and a reaction mixture was cooled to 0°C. NaH (suspension in oil, 60%) (0.64 g, 16 mmol) was added in one portion. Reaction mixture was stirred 18 h. DMF was removed under reduced pressure , to the residue water (20 ml) was added and extracted with ethyl ether (5 x 20 ml). Organic phase was washed with aq. solutions of NH_4Cl (20 ml) and $NaHCO_3$ (20 ml) and dried with $MgSO_4$. Solvents was removed under reduced pressure, and resulting oil was purified by flash column chromatography (AcOEt : Hexane; 1:6). The title compound (0.7 g, 1.56 mmol, 31%) was obtained as colourless liquid (90%:10% α:β).

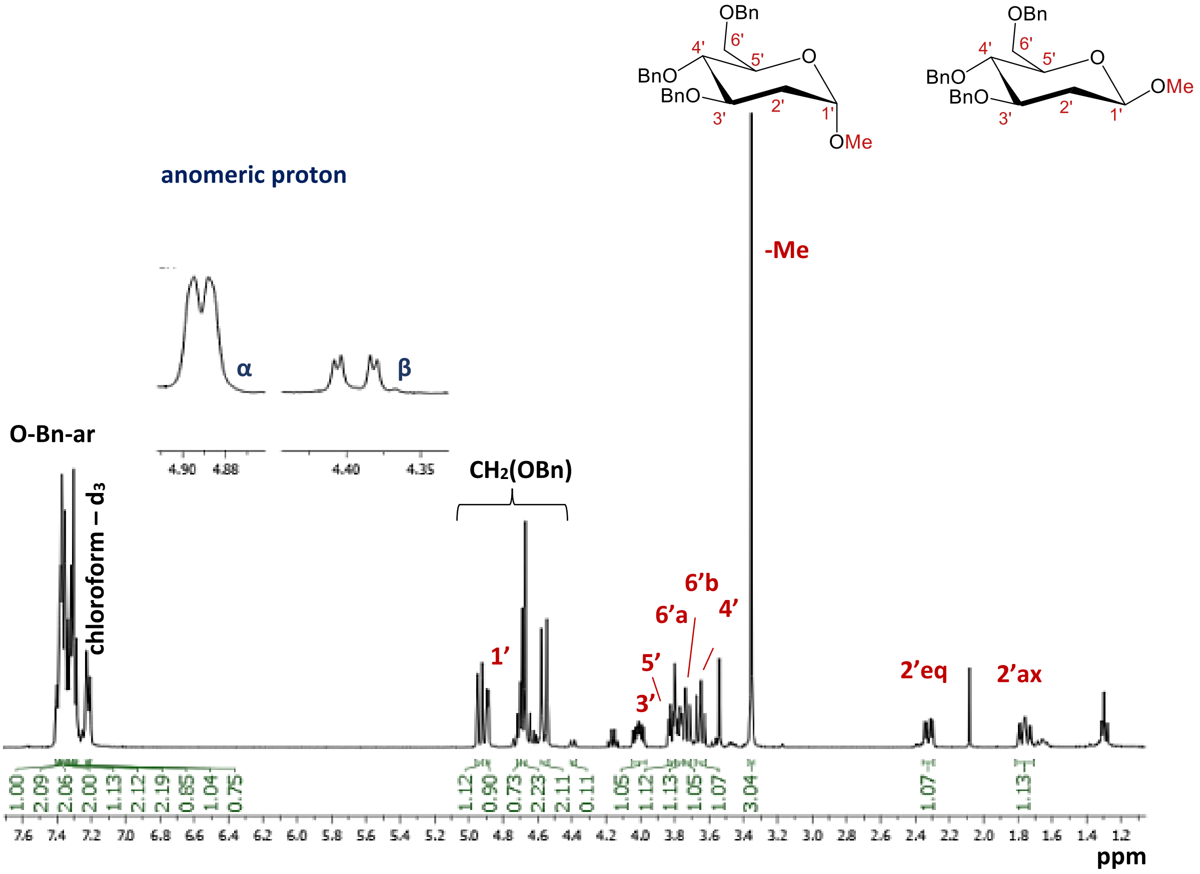

^{1}H NMR (500 MHz, $CDCl_3$) δ = 7.42 – 7.18 (m, 15H, **O-Bn-ar**), 4.96 – 4.54 (m, 6H, -C**H₂**(OBn)), 4.89 (dd, 1H, J^3=~0.0, 3.5 Hz, **1'**), 4.05 – 3.99 (m, 1H, **3'**), 3.82 (dd, 1H, J^3=4.5, 12.5 Hz, **6'a**), 3.79 – 3.75 (m, 1H, **5'**), 3.72 (dd, 1H, J^3=2.0, 12.5 Hz, **6'b**), 3.65 (t, 1H, ^3J = 11.5 Hz, **4'**), 3.35 (s, 3H, **-Me**), 2.35 – 2.29 (m, 1H, **2'eq**), 1.80 – 1.72 (m, 1H, **2'ax**)

2-Deoxy-3,4,6-tri-O-benzyl-(α,β)-D-glucopyranose

1-O-Methyl-2-deoxy-3,4,6-tri-O-benzyl-(α,β)-D-glucopyranose (147 mg, 0.33 mmol) was dissolved in AcOH (2 ml) and conc. aq HCl (0.5 ml) was added. Resulting mixture was stirred and heated to 55°C in oil bath for 1 h. AcOH was removed under reduced pressure, residue was dissolved in AcOEt (20 ml) washed with with aq. solution of $NaHCO_3$ (20 ml) and dried with $MgSO_4$. Solvents was removed under reduced pressure, and residue was purified by flash column chromatography (AcOEt : Hexane, 1:3). The title compound (0.065 g, 0.15 mmol, 45%) was obtained as colourless oil (80%:20% α:β).

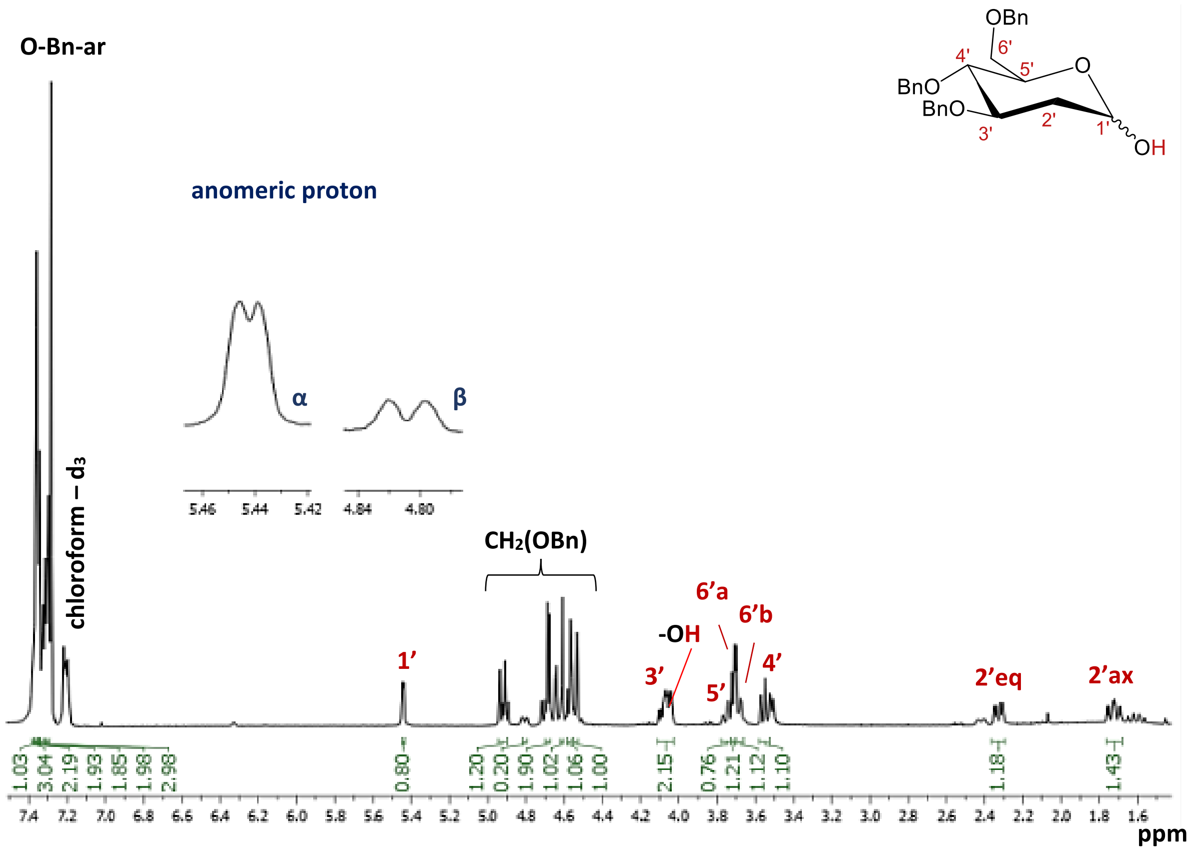

^{1}H NMR (500 MHz, $CDCl_3$) δ = 7.40 – 7.18 (m, 15H, **O-Bn-ar**), 4.44 (dd, 1H, J^3=~0.0, 3.5 Hz, **1'**), 4.95 – 4.50 (m, 6H, -C**H₂**(OBn)), 4.10 – 4.05 (m, 1H, **3'**), 4.07 (s, 1H, -O**H**), 3.79 – 3.73 (m, 1H, **5'**), 3.73 -3.71 (m, 1H, **6'a**), 3.69 - 3.67 (m, 1H, **6'b**), 3.55 (t, 1H, ^3J = 12.0 Hz, **4'**), 2.36 – 2.29 (m, 1H, **2'eq**), 1.77 – 1.69 (m, 1H, **2'ax**)

General procedure for synthesis of O-(3,4,6-tri-O-benzyl-(α,β)-2-deoxy-D-glucopyranosyl) secondary alkohols (2A, 2B, 2C)

3,4,6-tri-O-benzyl-2-deoxy-(α,β)-D-glucose (86 mg, 0.2 mmol) was dissolved in anhydrous DCM (3 ml). 4 Å molecular sieves were introduced. Then trichloroacetonitrile (200 µl, 2 mmol 10 eq) was added at room temperature. The reactions were initiated by the addition of a catalytic amount of NaH in oil. After 2 h, the resulting suspension was passed through a thin pad of silica gel system (ethyl acetate:hexanes; 1:3 with 1% Et_3N). The crude product (trichloroacetimidate 3,4,6-tri-O-benzyl-2-deoxy-(α,β)-D-glucose) was obtained in the form of a yellow oil and imidiately used for the next step). Crude trichloroacetoimidate 3,4,6-tri-O-benzyl-2-deoxy-(α,β)-D-glucose was dissolved in anhydrous DCM (3 ml). 4 Å molecular sieves were added to the solution, followed by (0.6 mmol) of appropriate alcohol (menthol or (S)-2-butanol) and a catalytic amount (15 µl) of TMSOTf. Reactions were carried out at room temperature for 24 h. Then a (300 µl) of Et_3N were added. The obtained mixture was concentrated and purified by flash chromatography (ethyl acetate:hexanes; 1:10, with 1% Et_3N) yielding suitable secondary alkyl 3,4,6-tri-O-benzyl-(α,β)-2-deoxy-D-glucopyranosides in the form of a white solids.

1D and 2D NMR spectra sets for the major products are presented in **Section 5.**

4. Structures of chiral probes

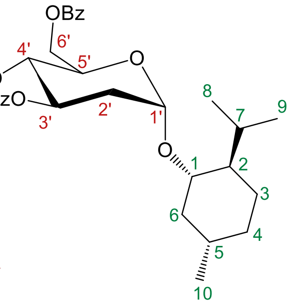

1A

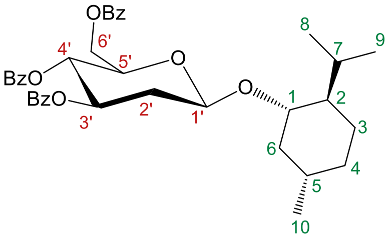

1B

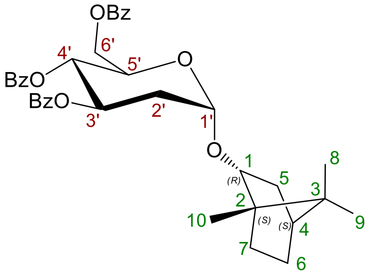

1C

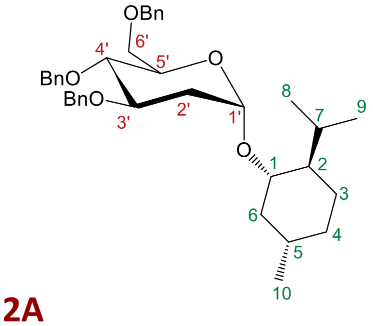

2A

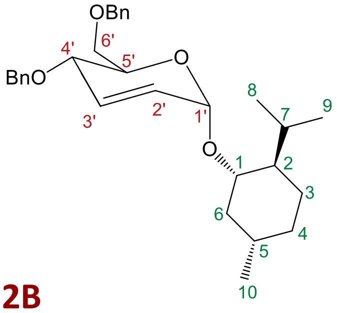

2B

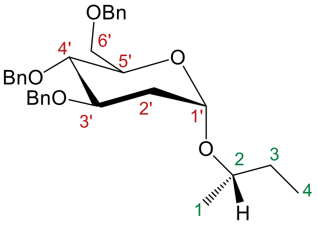

2C

5. NMR data assignments of 1A – 2C

5.1. Spectral sets for chiral probes

Table 1. Chemical shifts and coupling constants for molecule **1A** (^{1}H NMR)

^{1}H NMR<br>Data for 1A		
position	^{1}H δ (ppm)	$J_{H,H}$ (Hz)
**Aglycone Unit**		
**1**	3.61	10.7 (**2**), 10.7 (**6ax**), 4.1 (**6eq**)
**2**	1.39	10.7 (**1**), 11.1 (**3ax**), (**3eq**)*, 2.6 (**7**)
**3ax**	1.06	11.1 (**2**), 13,1 (**3eq**), (**4ax**)*, (**4eq**)*
**3eq**	1.72	(**2**)*, 13.1 (**3ax**), (**4ax**)*, (**4eq**)*
**4ax**	0.92	(**3ax**)*, (**3eq**)*, 12.3 (**4eq**), 12.1 (**5**)
**4eq**	1.71	(**3ax**)*, (**3eq**)*, 12.3 (**4ax**), 5.7 (**5**)
**5**	1.39	12.1 (**4ax**), 5.7 (**4eq**), (**6ax**)*, (**6eq**)*, 7.0 (**10**)
**6ax**	0.89	10.7 (**1**), (**5**)*, 12.4 (**6eq**)
**6eq**	2.25	4.1 (**1**), (**5**)*, 12.4 (**6ax**)
**7**	2.49	2.6 (**2**), 7.0 (**8**), 7.0 (**9**)
**8**	0.89	7.0 (**7**)
**9**	1.01	7.0 (**7**)
**10**	0.95	7.0 (**5**)
**Probe Moiety**		
**1'**	5.43	0.8 (**2'eq**), 4.2 (**2'ax**)
**2'ax**	2.18	4.2 (**1'**), 12.9 (**2'eq**), 12.0 (**3'**)
**2'eq**	2.46	0.8 (**1'**), 12.9 (**2'ax**), 5.6 (**3'**)
**3'**	5.76	12.0 (**2'ax**), 5.6 (**2'eq**), 9.9 (**4'**)
**4'**	5.63	9.9 (**3'**), 9.9 (**5'**)
**5'**	4.49	9.9 (**4'**), 4.6 (**6'a**), 2.4 (**6'b**)
**6'a**	4.54	4.6 (**5'**), 12.1 (**6'b**)
**6'b**	4.62	2.4 (**5'**), 12.1 (**6'a**)
**O-Bz (15H)**	7.43 – 8.09	
*These coupling constants could not be measured. Signal pattern remains partially unclear due to serve signal overlap ang higher order effects		

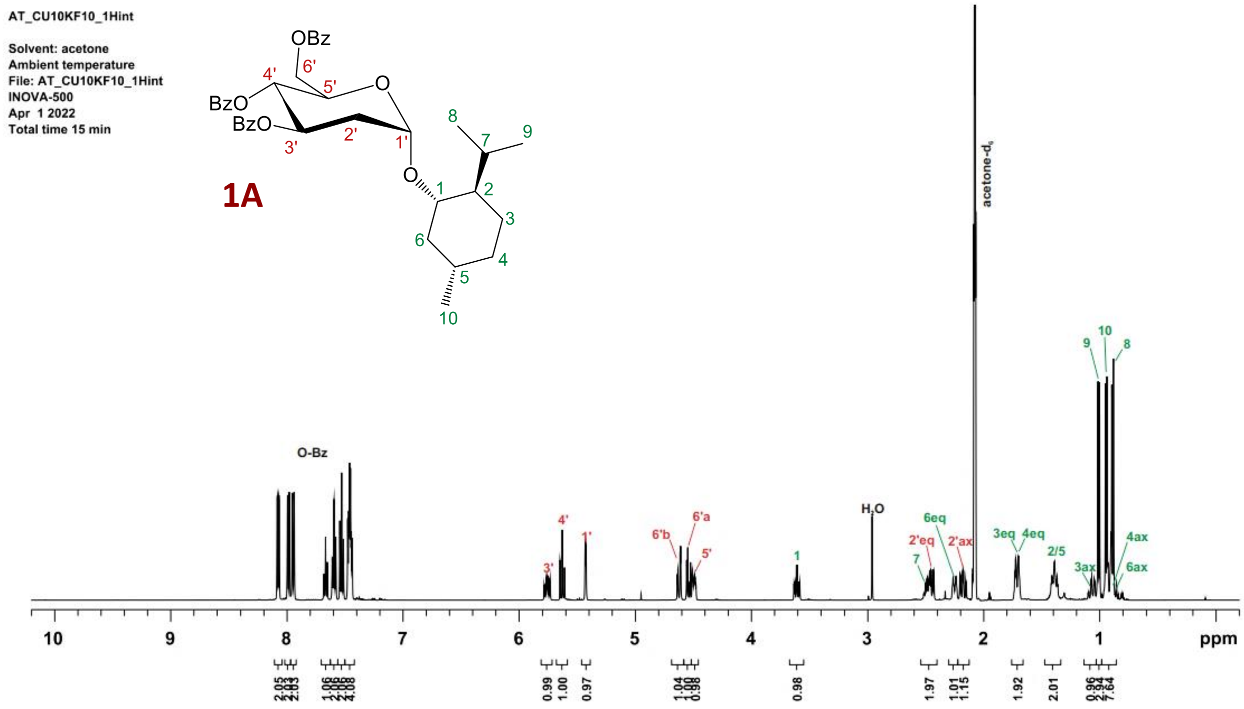

Figure.1. ^{1}H NMR spectrum for **1A**

Table 2. Chemical shifts for molecule **1A** (^{13}C NMR)

^{13}C NMR

^{13}C NMR Data for **1A**		
position		^{13}C δ (ppm)
**Aglycone Unit**		
**1**	CH	75.0
**2**	CH	48.1
**3**	CH_2	22.7
**4**	CH_2	34.3
**5**	CH	31.2
**6**	CH_2	39.4
**7**	CH	25.3
**8**	CH_3	15.2
**9**	CH_3	20.8
**10**	CH_3	21.8
**Probe Moiety**		
**1'**	anomeric carbon	92.9
**2'**		35.8
**3'**		70.2
**4'**		70.6
**5'**		68.9
**6'**		63.3
**O-Bz**	18 aromatic carbons	129.0 - 133.4
**C=O**	3 carbonyl carbons	165.2<br>165.3<br>165.6

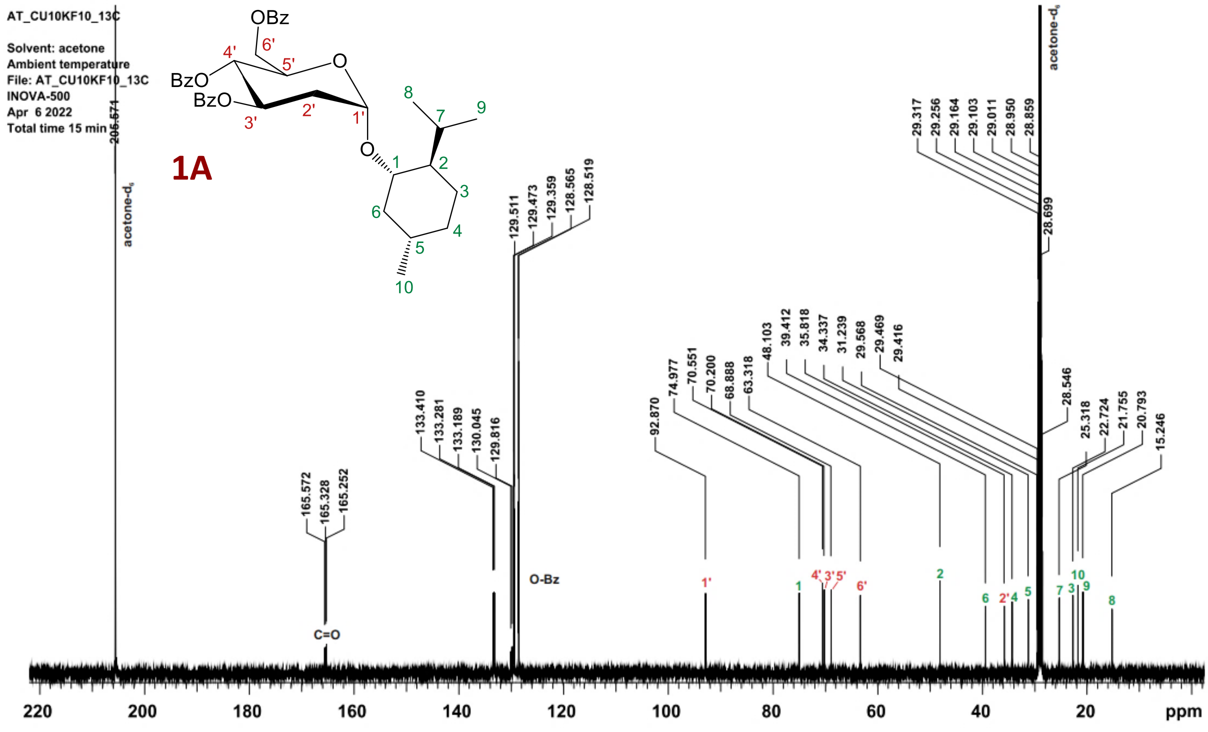

Figure.2. ^{13}C NMR spectrum for **1A**

DQF-COSY

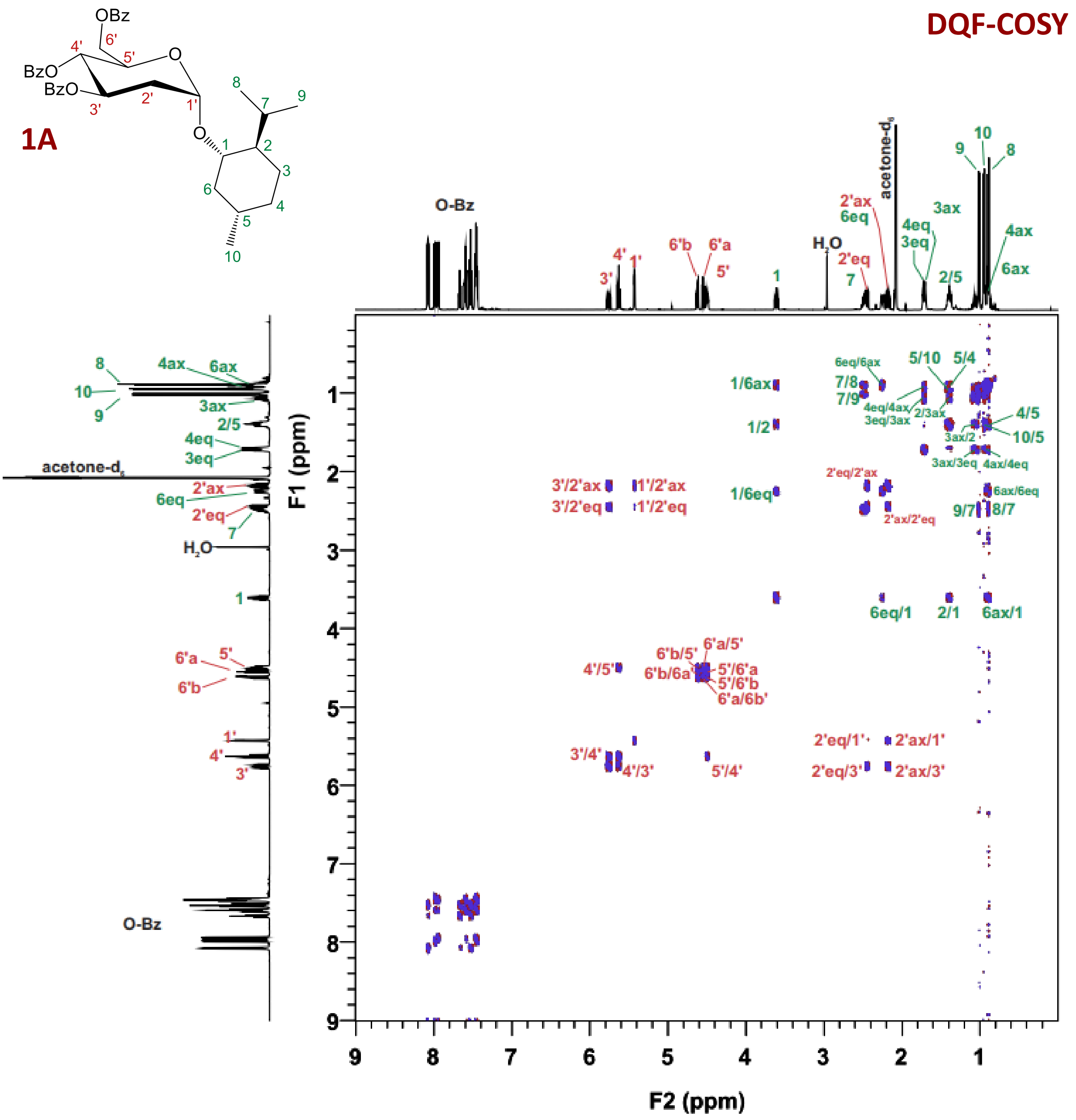

Figure.3. DQF-COSY spectrum for **1A**

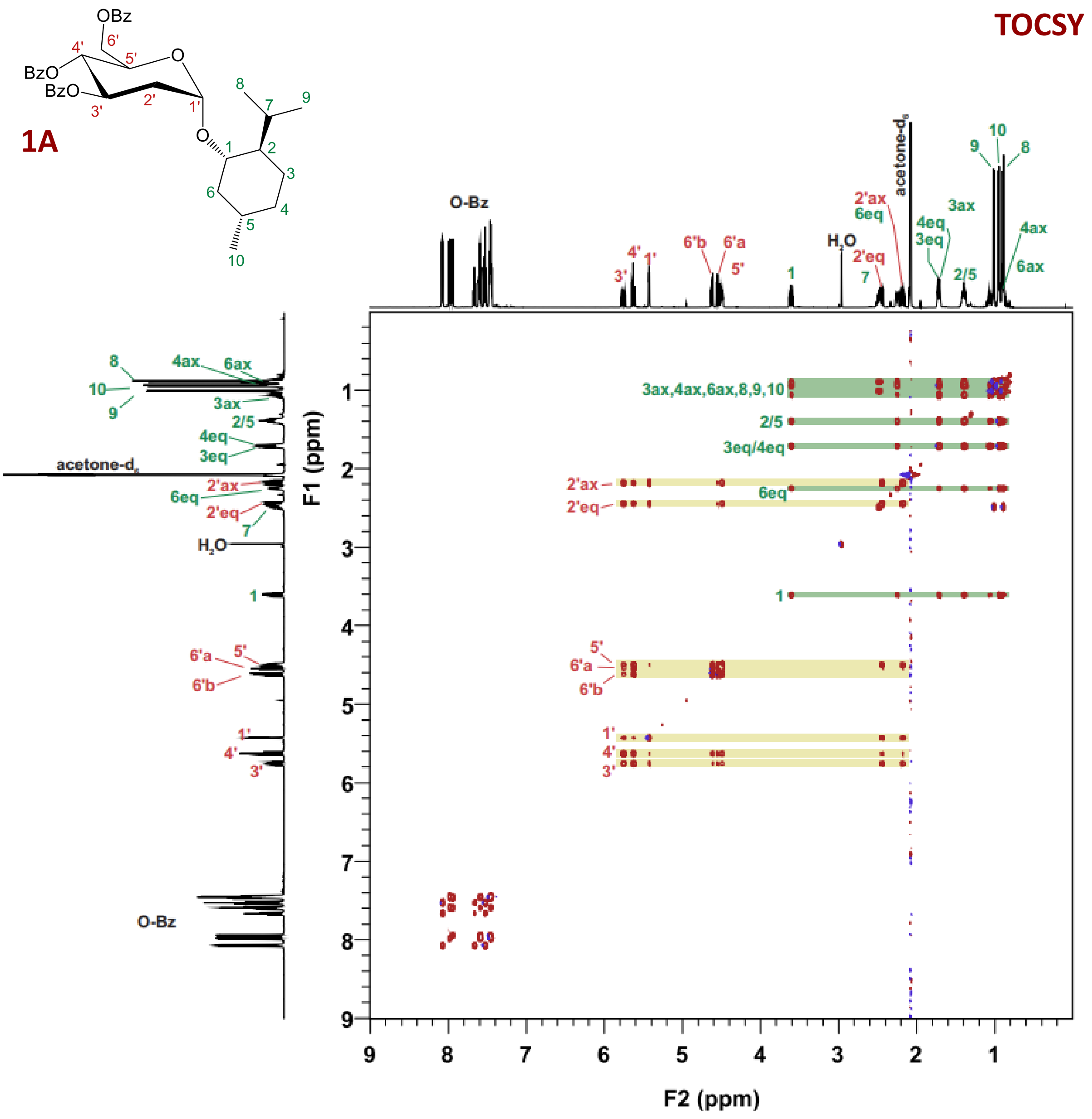

Figure.4. TOCSY spectrum for **1A**

HSQC

1A

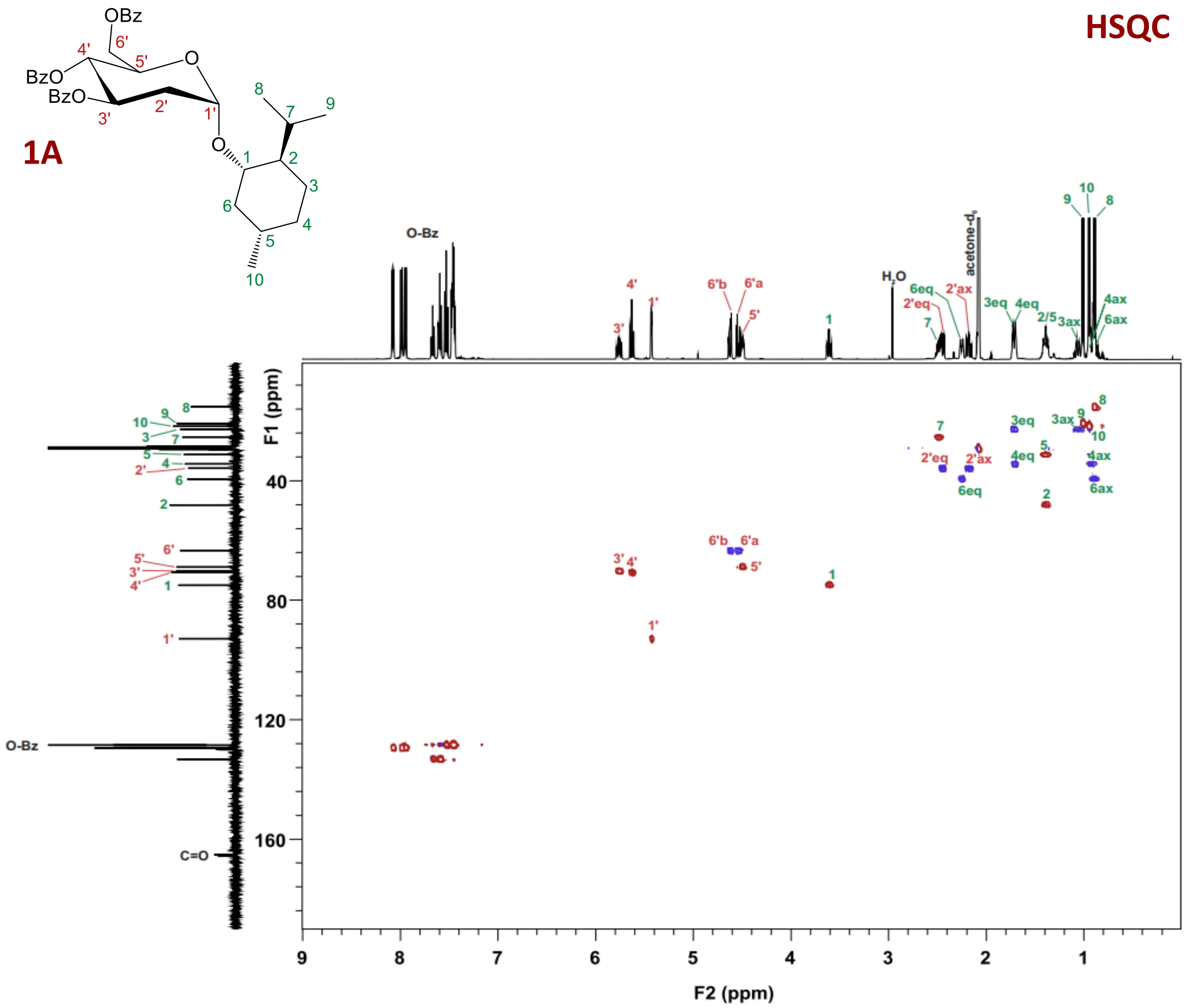

Figure.5. HSQC spectrum for **1A**

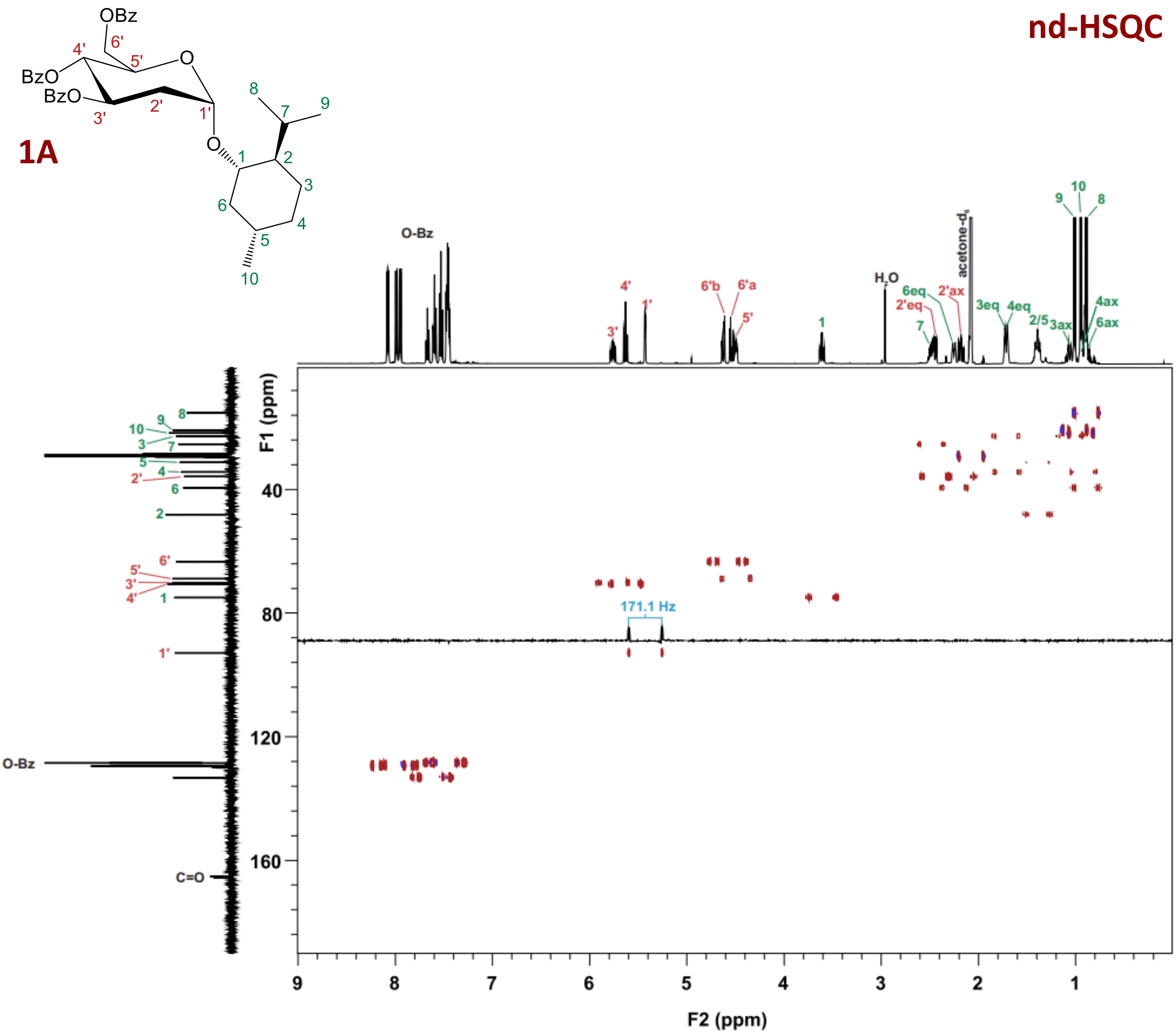

Figure.6. nd-HSQC spectrum for **1A**

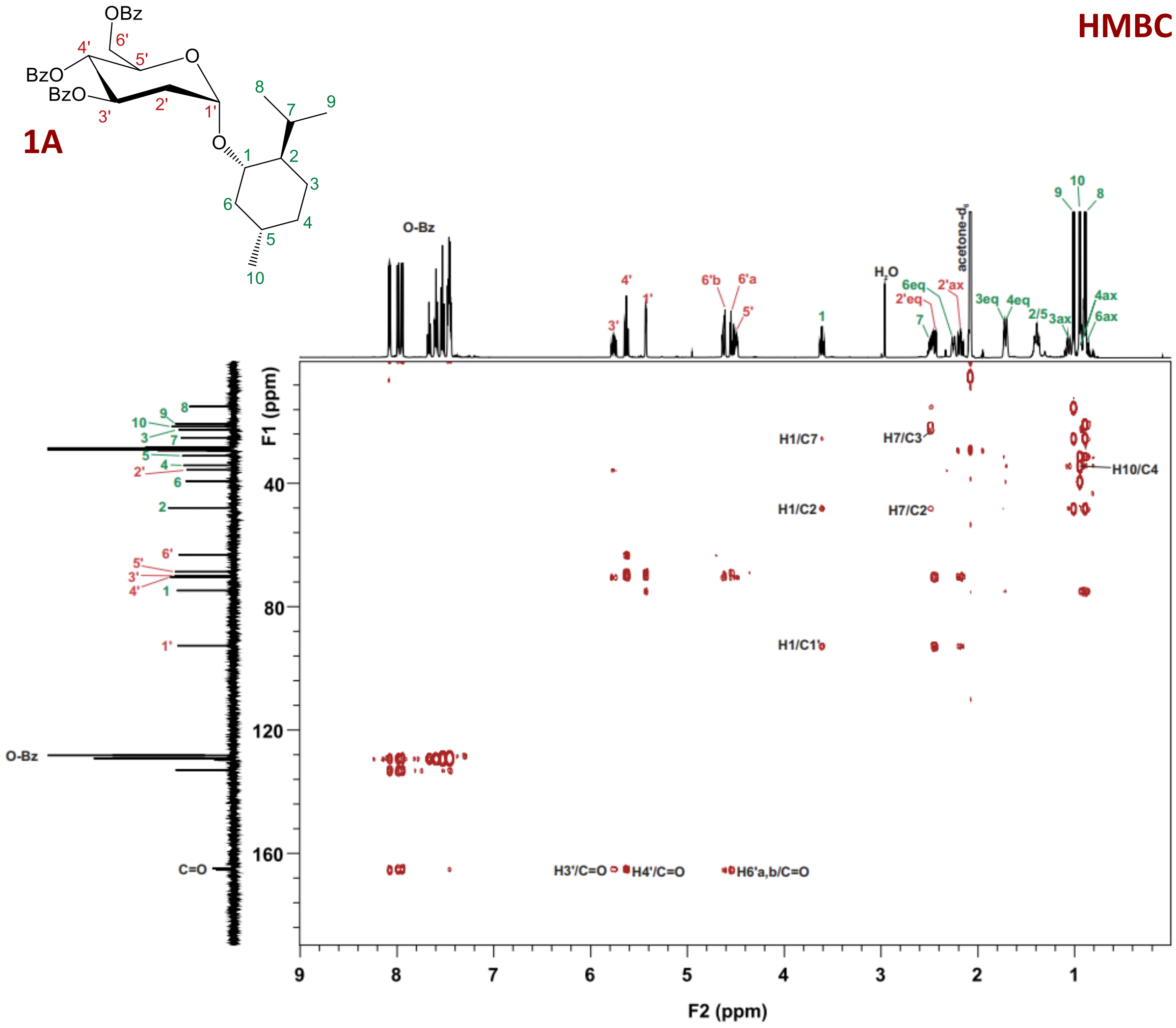

Figure.7. HMBC spectrum for **1A**

H2BC

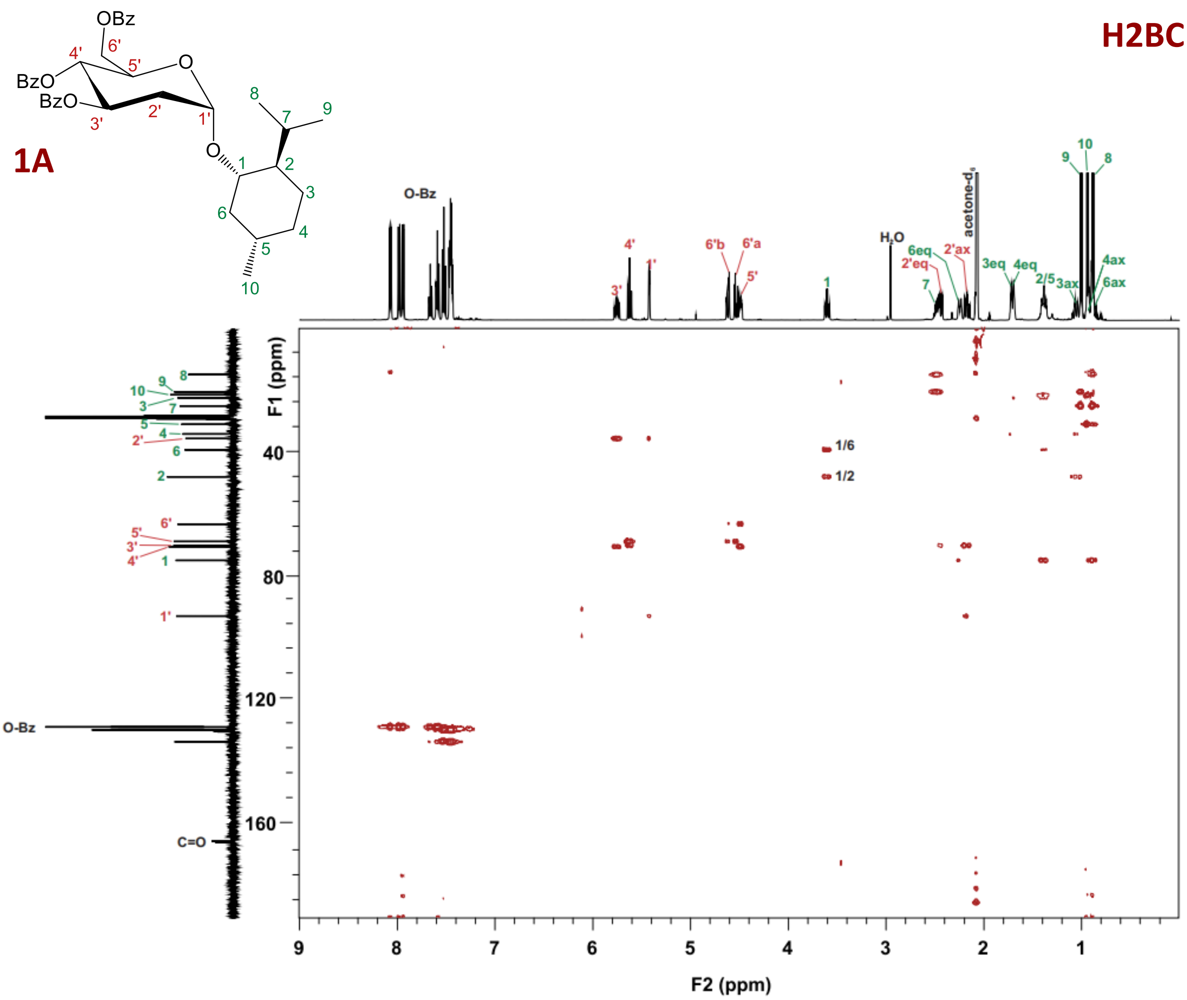

Figure.8. H2BC spectrum for **1A**

Table.3. Diagnostic ROE to proton for **1A**

2D ROESY NMR Data for **1A**	
position	diagnostic **ROE to proton**
**Aglycone Unit**	
**1**	3ax
**Probe Moiety**	
**1'**	1, 6eq, 8
**4'**	2'ax
**5'**	7, 8

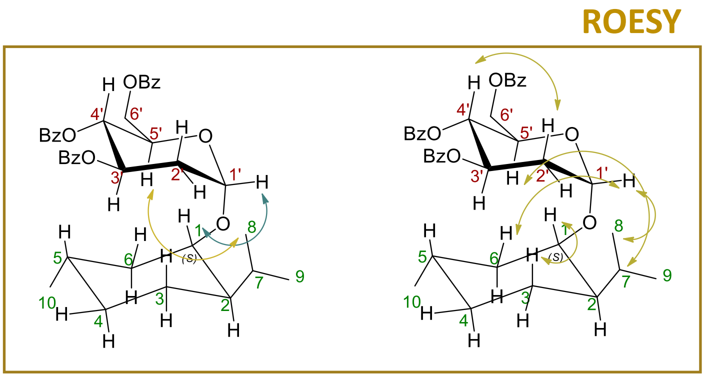

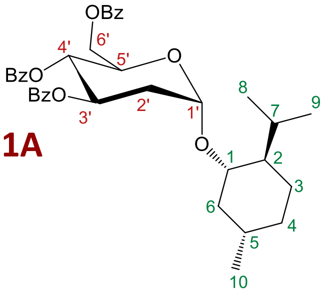

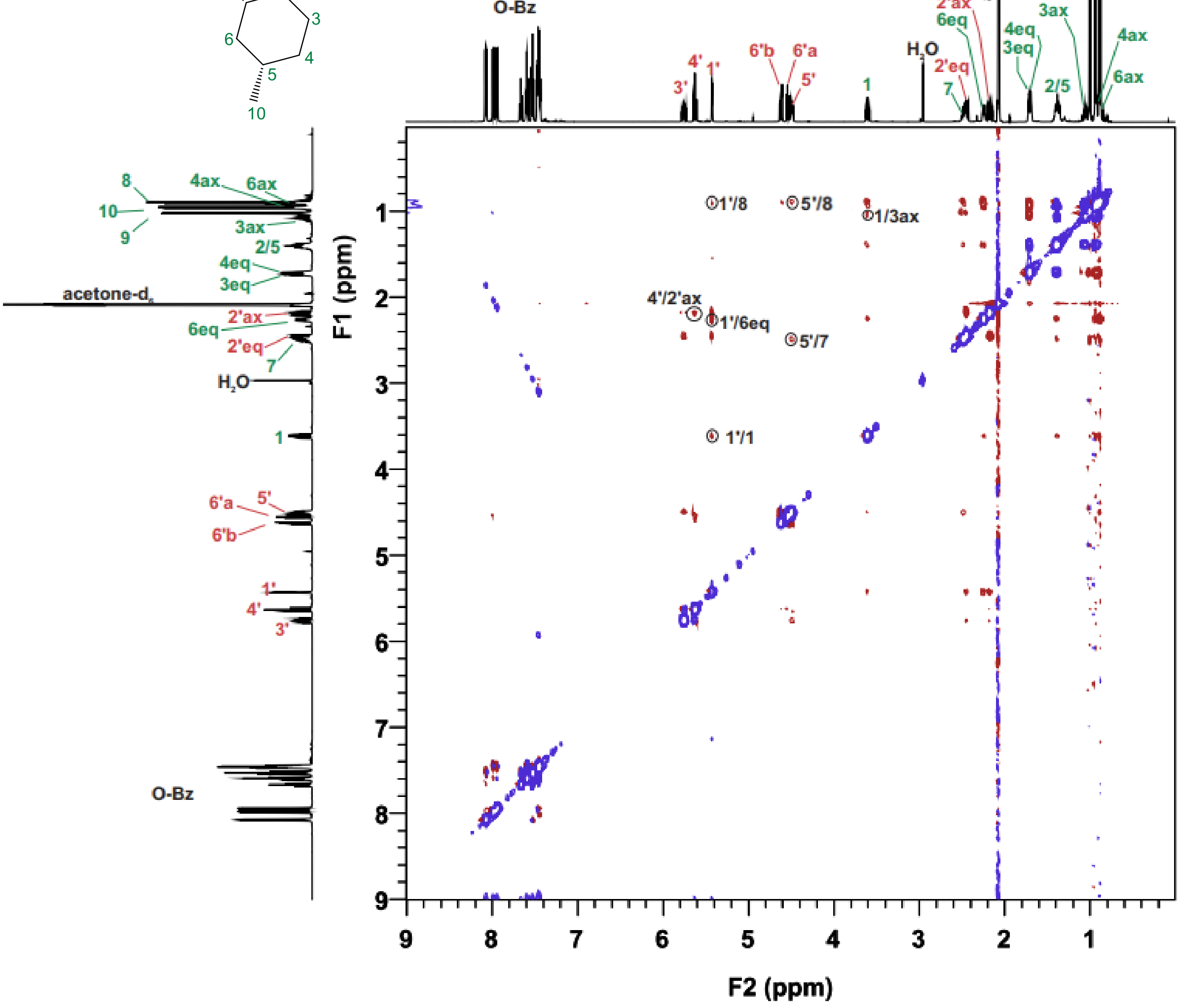

Figure.9. ROESY spectrum for **1A**

Table 4. Chemical shifts and coupling constants for molecule **1B** (^{1}H NMR)

^{1}H NMR

^{1}H NMR Data for **1B**		
position	^{1}H δ (ppm)	$J_{H,H}$ (Hz)
**Aglycone Unit**		
**1**	3.51	10.3 (**2**), 4.0 (**6eq**), 10.3 (**6ax**)
**2**	1.24	10.3 (**1**), (**3ax**)*, 5.6 (**3eq**), 3.6 (**7**)
**3ax**	1.02	(**2**)*, 12.8 (**3eq**), 12.2 (**4ax**), (**4eq**)*
**3eq**	1.63	5.6 (**2**), 12.8 (**3ax**), (**4ax**)*, (**4eq**)*
**4ax**	0.82	12.2 (**3ax**), (**3eq**)*, 12.8 (**4eq**), 11.3 (**5**)
**4eq**	1.63	(**3ax**)*, (**3eq**)*, 12.8 (**4ax**), (**5**)*
**5**	1.33	11.3 (**4ax**), (**4eq**)*, 12.2 (**6ax**), 4.8 (**6eq**), 6.9 (**10**)
**6ax**	1.01	10.3 (**1**), 12.2 (**5**), (**6eq**)*
**6eq**	2.33	4.0 (**1**), 4.8 (**5**), (**6ax**)*
**7**	2.19	3.6 (**2**), 6.9 (**8**), 6.9 (**9**)
**8**	0.85	6.9 (**7**)
**9**	0.91	6.9 (**7**)
**10**	0.81	6.9 (**5**)
**Probe Moiety**		
**1'**	5.11	1.7 (**2'eq**), 9.6 (**2'ax**)
**2'ax**	1.95	9.6 (**1'**), 11.9 (**2'eq**), 10.3 (**3'**)
**2'eq**	2.59	1.7 (**1'**), 11.9 (**2'ax**), 4.5 (**3'**)
**3'**	5.60	10.3 (**2'ax**), 4.5 (**2'eq**), 9.8 (**4'**)
**4'**	5.47	9.8 (**3'**), 9.8 (**5'**)
**5'**	4.30	9.8 (**4'**), 6.2 (**6'a**), 2.8 (**6'b**)
**6'a**	4.54	6.2 (**5'**), 11.8 (**6'b**)
**6'b**	4.62	2.8 (**5'**), 11.8 (**6'a**)
**O-Bz (15H)**	7.42 – 8.08	
*These coupling constants could not be measured. Signal pattern remains partially unclear due to serve signal overlap ang higher order effects		

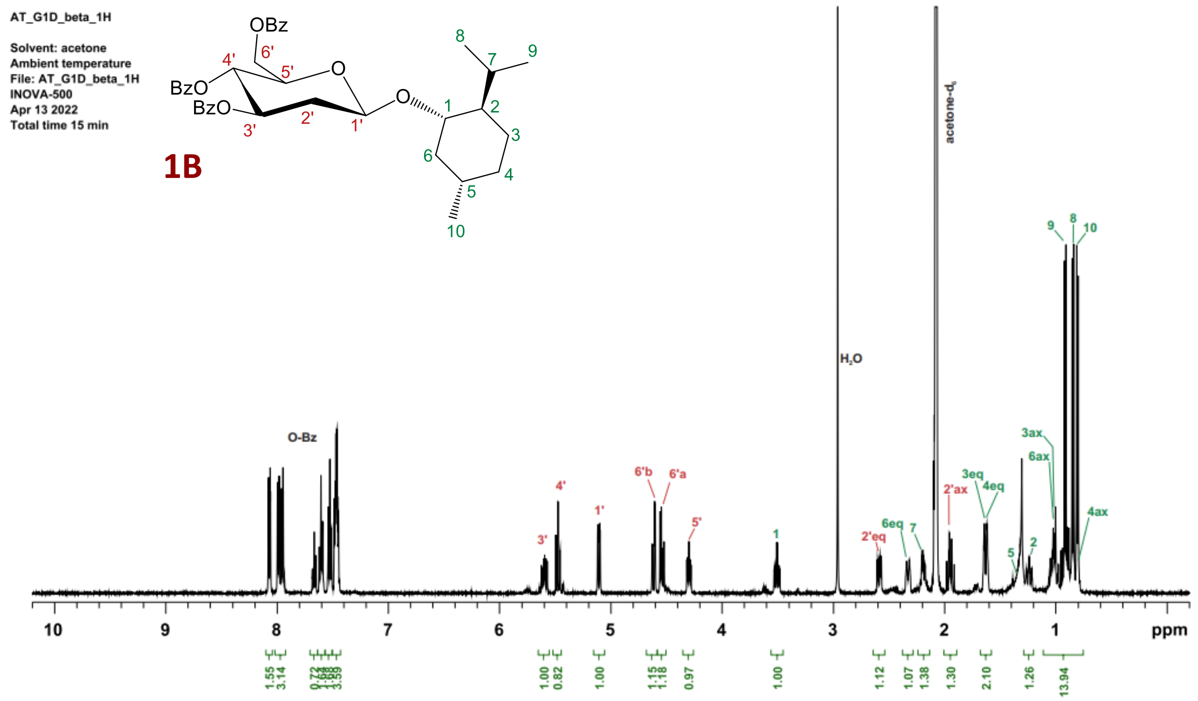

Figure.10. ^{1}H NMR spectrum for **1B**

Table 5. Chemical shifts for molecule **1B** (^{13}C NMR)

^{13}C NMR

^{13}C NMR Data for **1B**		
position		^{13}C δ (ppm)
**Aglycone Unit**		
**1**	CH	81.5
**2**	CH	48.5
**3**	CH_2	23.1
**4**	CH_2	34.2
**5**	CH	31.5
**6**	CH_2	43.4
**7**	CH	25.6
**8**	CH_3	15.8
**9**	CH_3	20.5
**10**	CH_3	21.7
**Probe Moiety**		
**1'**	anomeric carbon	100.9
**2'**		36.8
**3'**		71.6
**4'**		70.7
**5'**		71.6
**6'**		63.5
**O-Bz**	18 aromatic carbons	128.5 - 133.5
**C=O**	3 carbonyl carbons	165.2<br>165.4<br>165.6

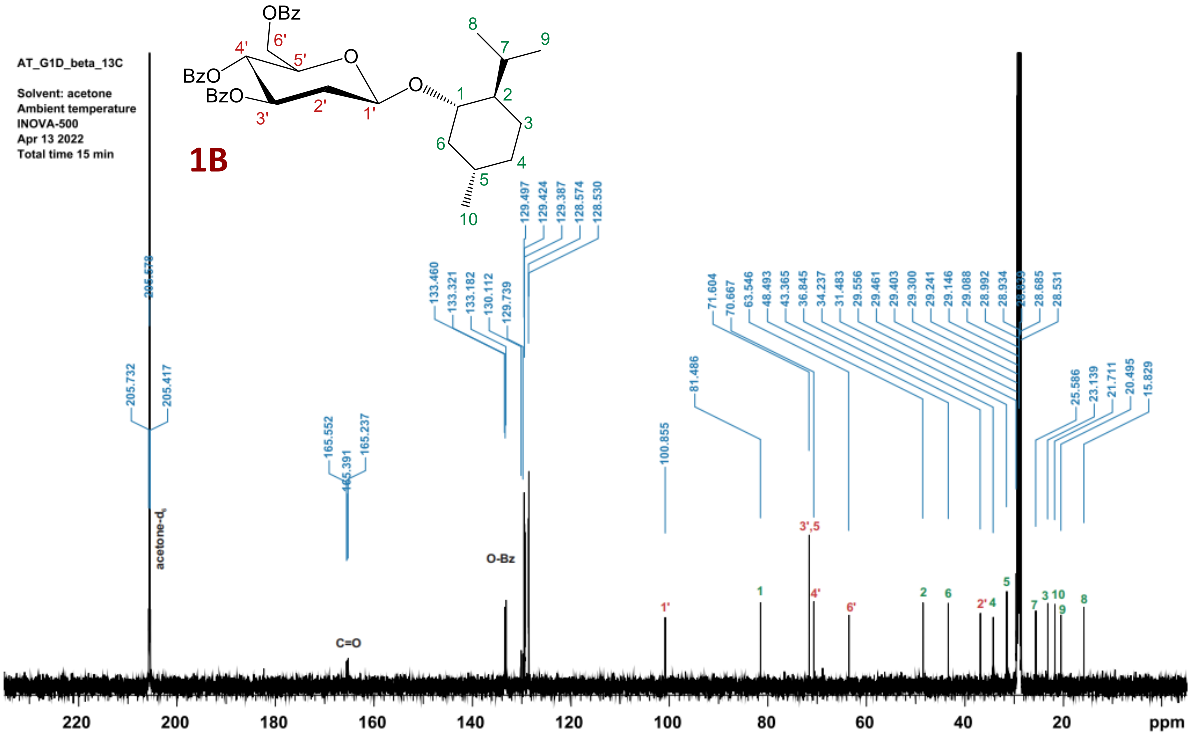

Figure.11. ^{13}C NMR spectrum for **1B**

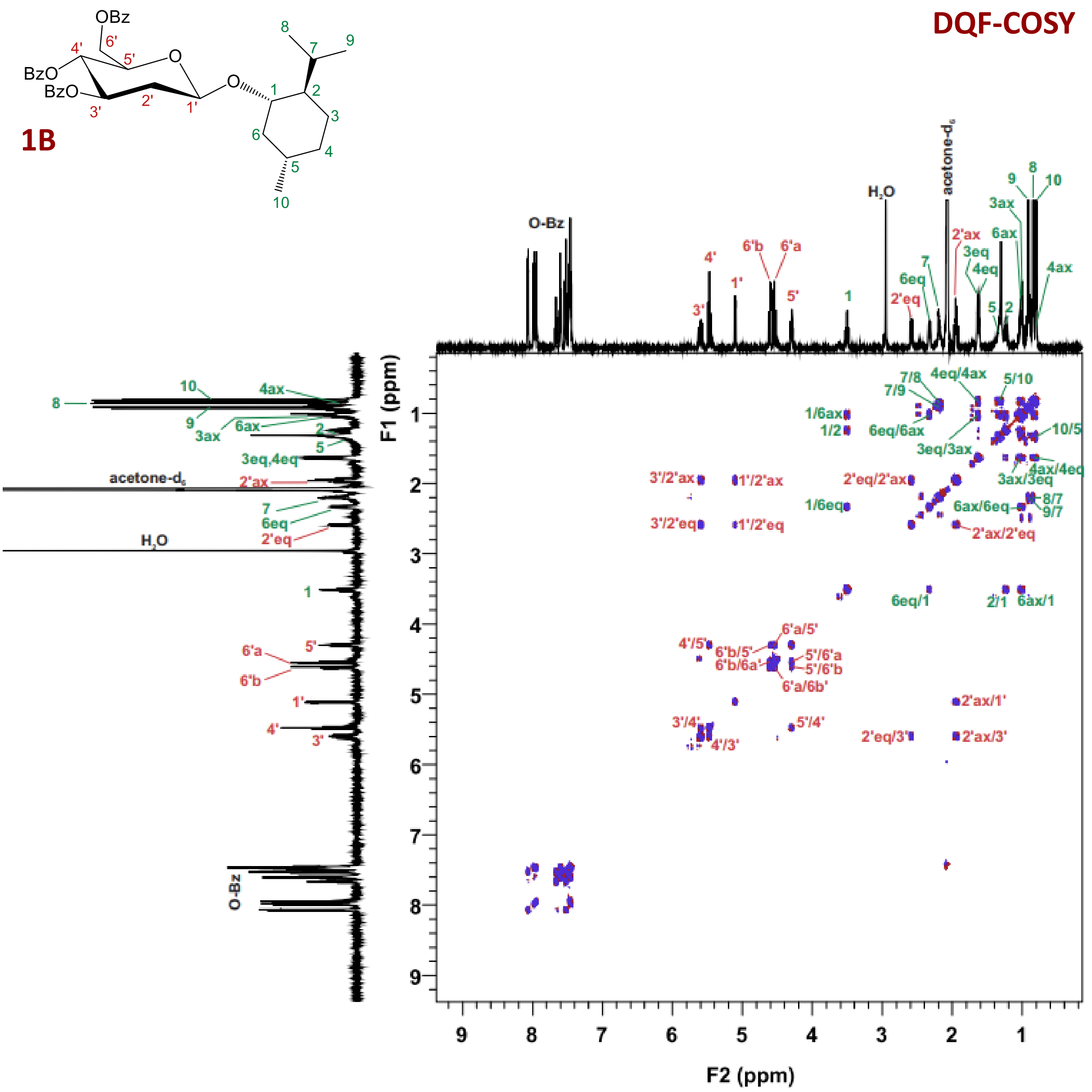

Figure.12. DQF-COSY spectrum for **1B**

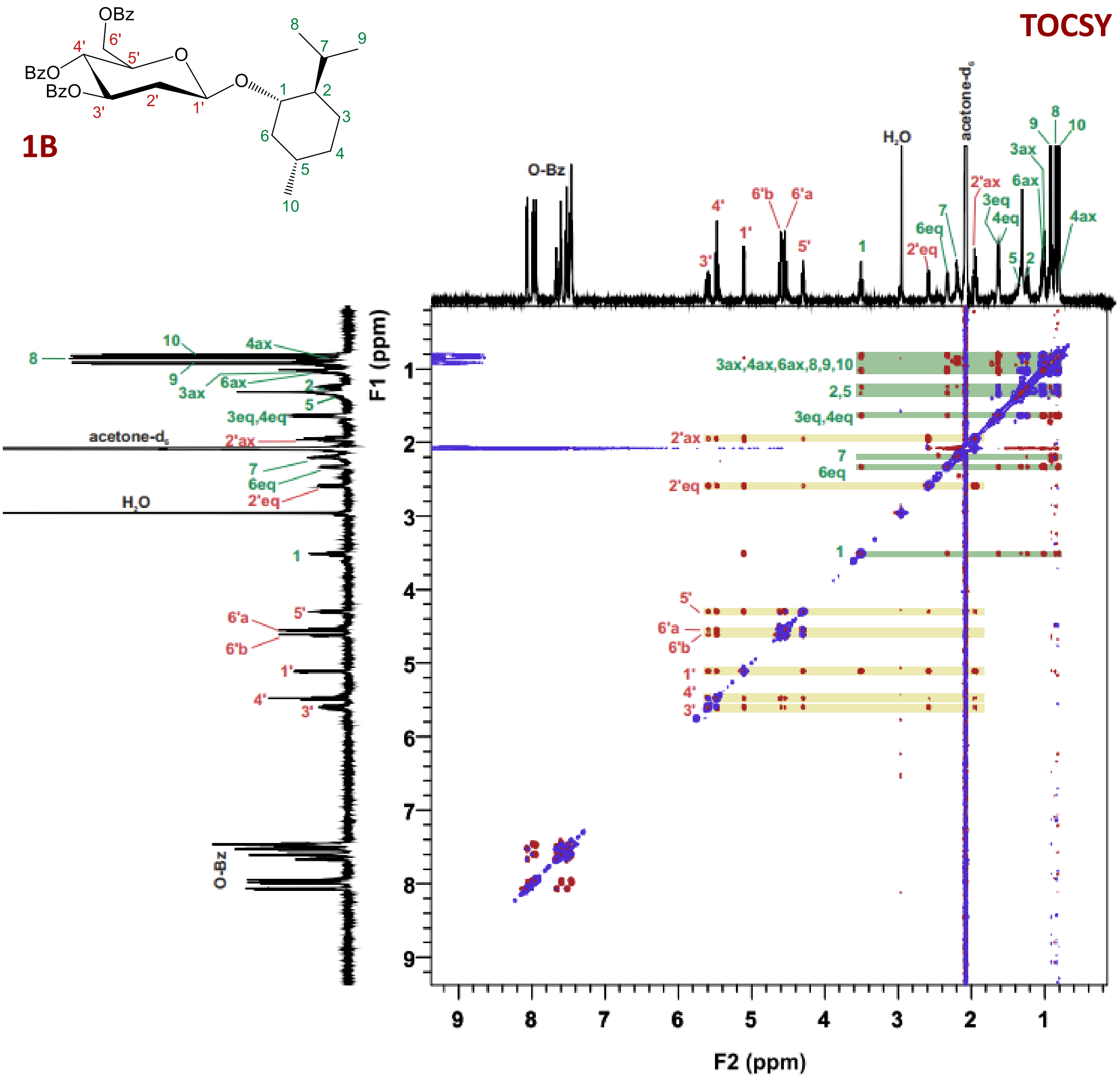

Figure..13 TOCSY spectrum for **1B**

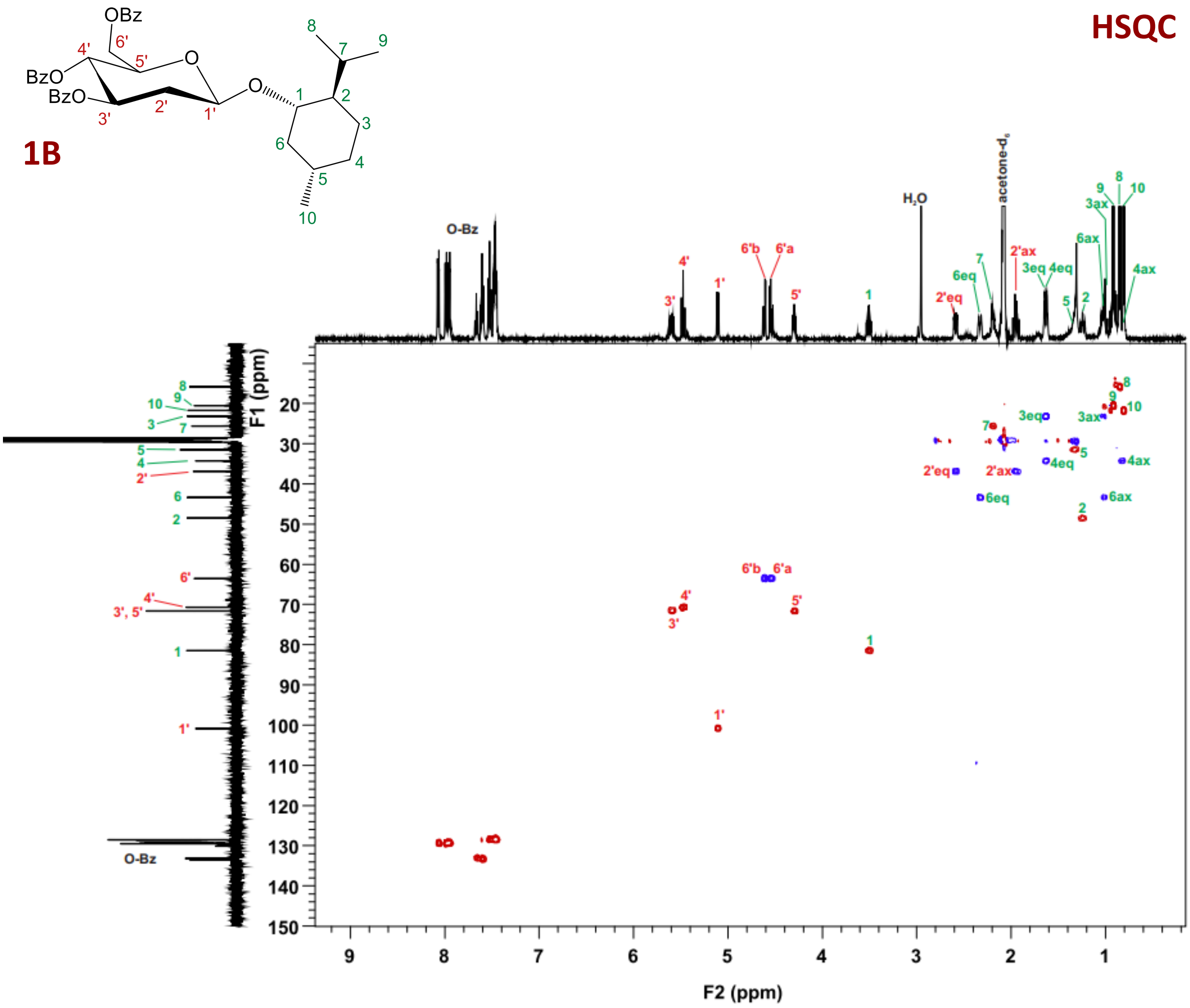

Figure.14. HSQC spectrum for **1B**

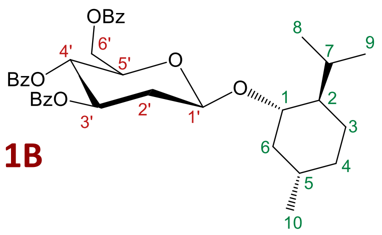

1B

nd-HSQC

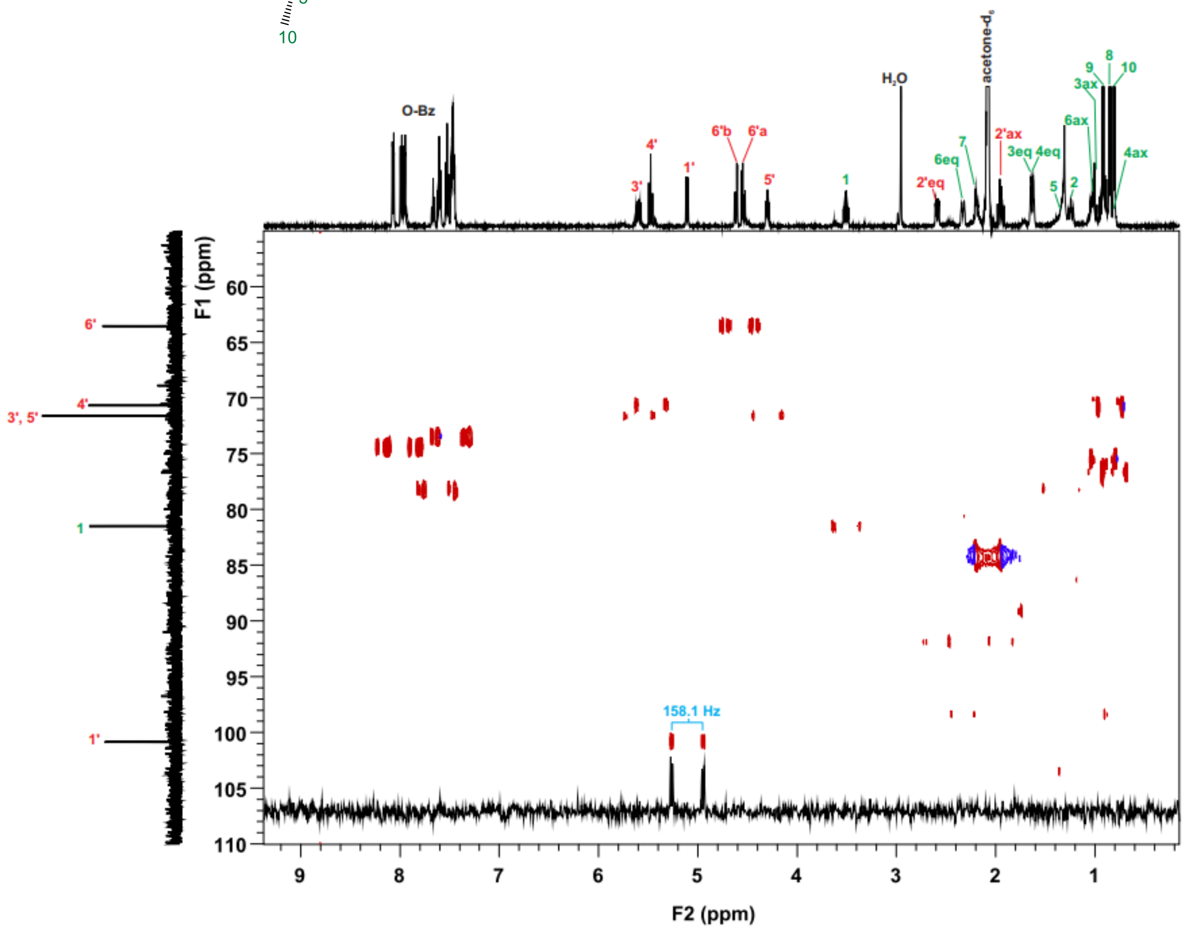

Figure.15. HSQC (without decoupling) spectrum for **1B**

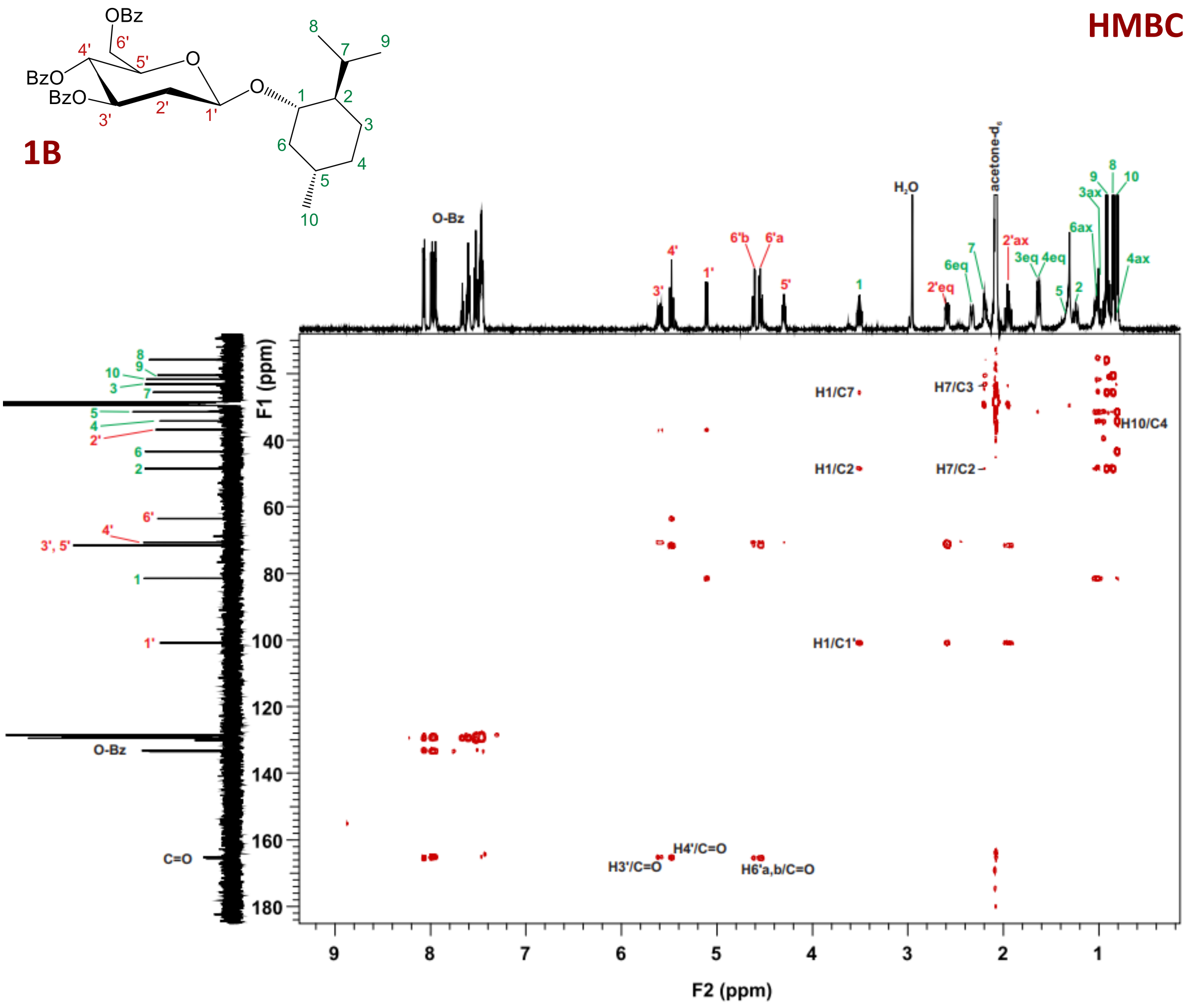

Figure.16. HMBC spectrum for **1B**

Table.6. Diagnostic ROE to proton for **1B**

2D ROESY NMR Data for **1B**	
position	diagnostic **ROE to proton**
**Probe Moiety**	
**1'**	1, 8, 3', 5', 6eq, 7
**3'**	2'eq, 5', 1'
**4'**	2'ax
**5'**	1'. 3'

ROESY

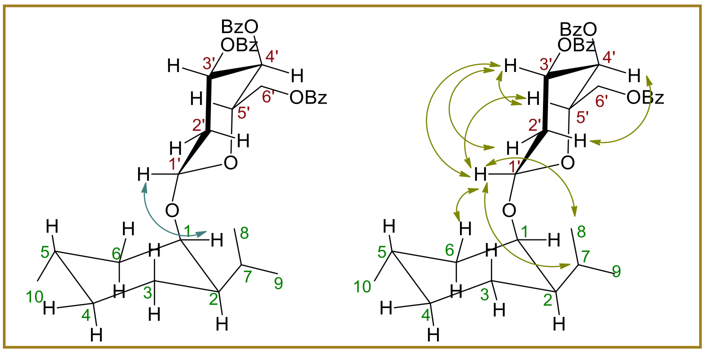

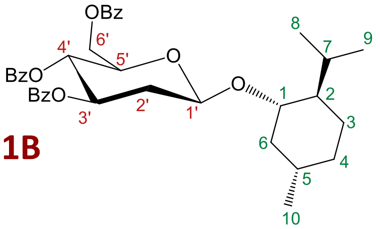

1B

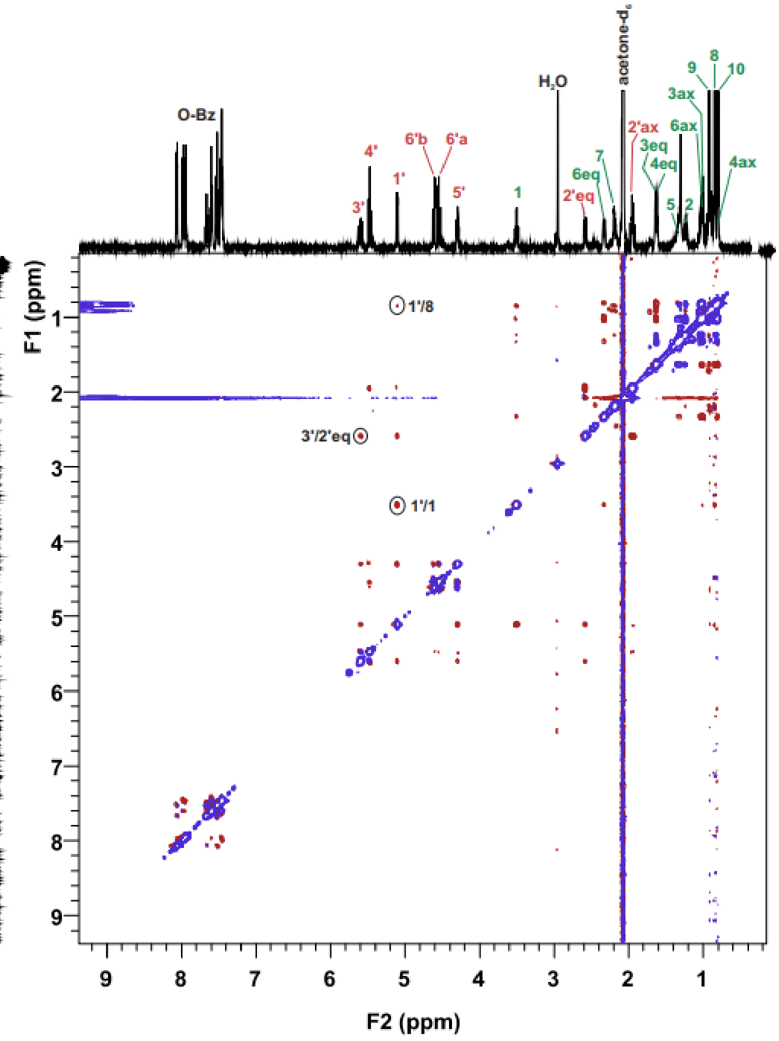

Figure.17. ROESY spectrum for **1B**

^{1}H NMR

Table 7. Chemical shifts and coupling constants for molecule **1C** (^{1}H NMR)

^{1}H NMR Data for **1C**		
position	^{1}H δ (ppm)	$J_{H,H}$ (Hz)
**Aglycone Unit**		
**1**	4.00	3.4 (**5ex**), 10.5 (**5en**)
**4**	1.61	~ 0.0 (**5ex**), 4.6 (**5en**), ~ 0.0 (**6ex**), 4.6 (**6en**)
**5ex**	1.35	3.4 (**1**), ~ 0.0 (**4**), 12.7 (**5en**)
**5en**	2.28	10.5 (**1**), 4.6 (**4**), 12.7 (**5ex**)
**6ex**	1.35	~ 0.0 (**4**), 12.6 (**6en**), (**7ex**)*, (**7en**)*
**6en**	1.78	4.6 (**4**), 12.6 (**6ex**), (**7ex**)*, 5.6 (**7en**)
**7ex**	1.32	(**6ex**)*, (**6en**)*, 12.6 (**7en**)
**7en**	2.17	(**6ex**)*, 5.6 (**6en**), 12.6 (**7ex**)
**8**	0.90	
**9**	0.88	
**10**	0.94	
**Probe Moiety**		
**1'**	5.20	0.8 (**2'ax**), 3.3 (**2'eq**)
**2'ax**	2.12	0.8 (**1**), 12.9 (**2'eq**), 9.5 (**3'**)
**2'eq**	2.57	3.3 (**1**), 12.9 (**2'ax**), 5.5 (**3'**)
**3'**	5.80	9.5 (**2'ax**), 5.5 (**2'eq**), 9.5 (**4'**)
**4'**	5.58	9.5 (**3'**), 9.5 (**5'**)
**5'**	4.58	9.5 (**4'**), 5.5 (**6'a**), 2.6 (**6'b**)
**6'a**	4.52	5.5 (**5'**), 11.9 (**6'b**)
**6'b**	4.61	2.6 (**5'**), 11.9 (**6'a**)
**O-Bz (15H)**	7.41-8.09	
*These coupling constants could not be measured. Signal pattern remains partially unclear due to serve signal overlap and higher order effects		

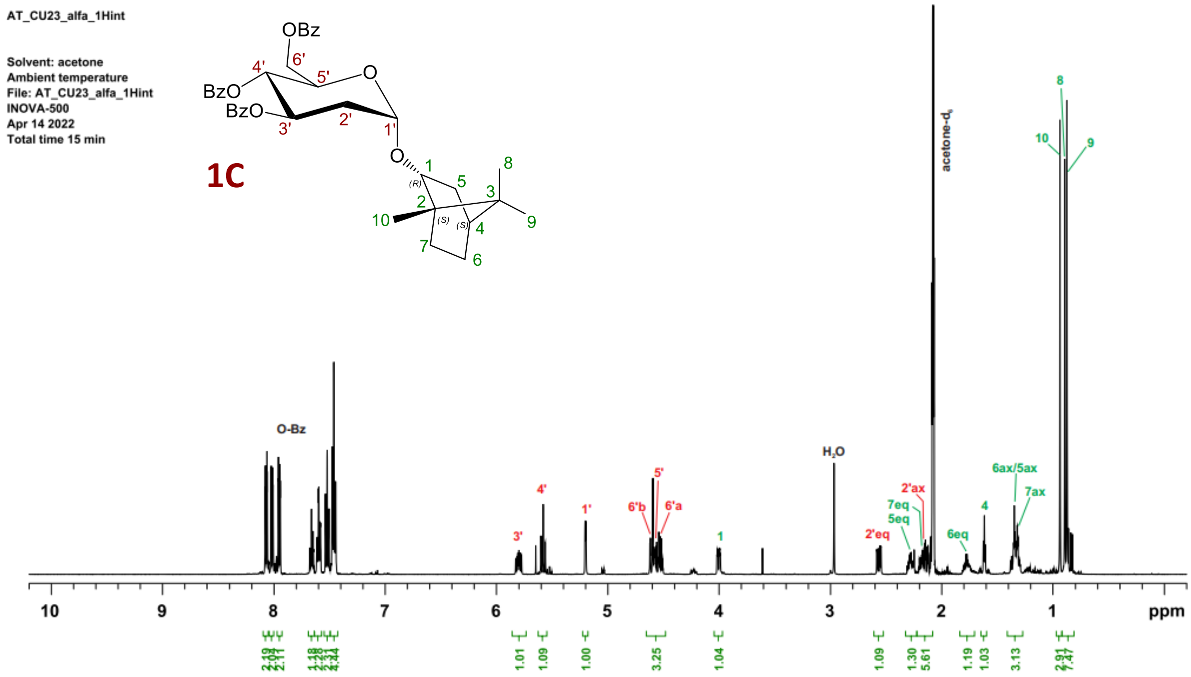

Figure.18. ^{1}H NMR spectrum for **1C**

Table 8. Chemical shifts for molecule **1C** (^{13}C NMR)

^{13}C NMR Data for **1C**		
position		^{13}C δ (ppm)
**Aglycone Unit**		
**1**	CH	84.6
**2**	C (4º)	49.3
**3**	C (4º)	47.2
**4**	CH	45.0
**5**	CH_2	36.8
**6**	CH_2	28.1
**7**	CH_2	26.6
**8**	CH_3	19.2
**9**	CH_3	18.2
**10**	CH_3	13.3
**Probe Moiety**		
**1'**	anomeric carbon	98.7
**2'**		35.6
**3'**		70.7
**4'**		70.1
**5'**		68.7
**6'**		63.5
**O-Bz**	18 aromatic carbons	128.5 - 133.4
**C=O**	3 carbonyl carbons	165.3<br>165.4<br>165.6

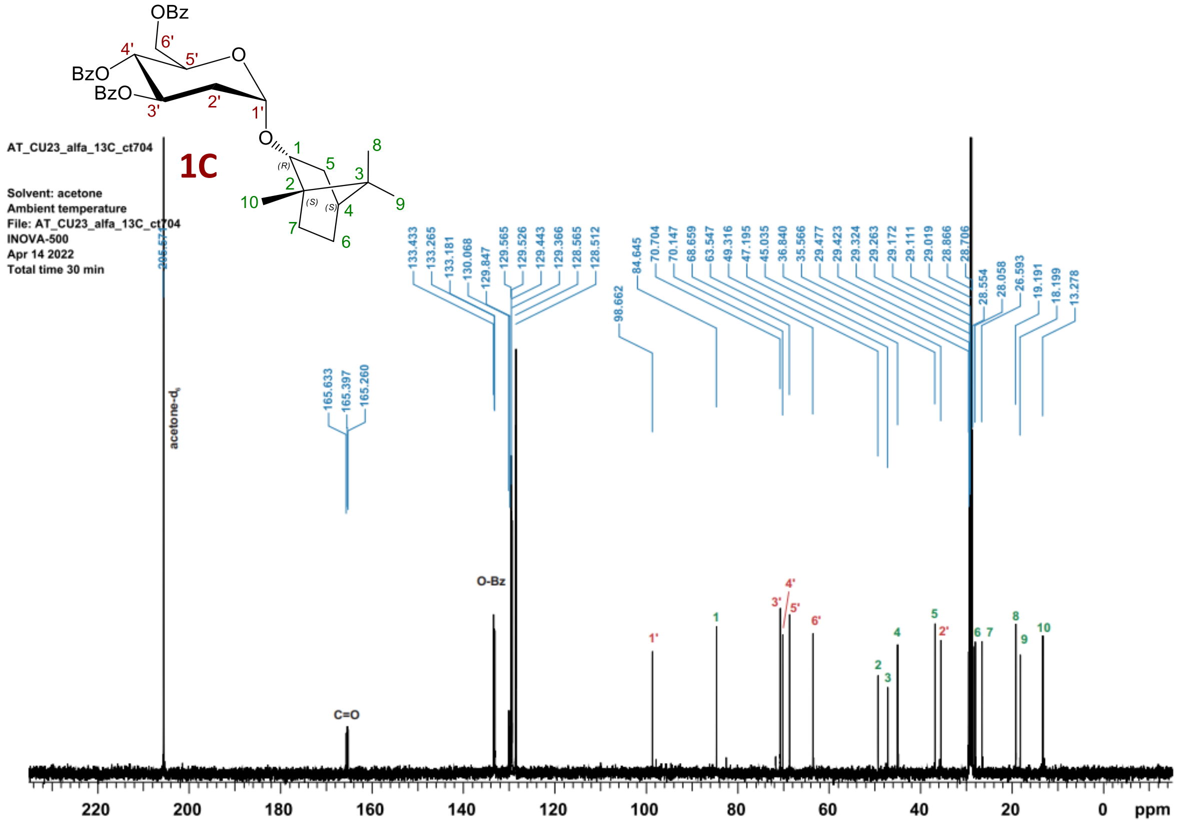

Figure.19. ^{13}C NMR spectrum for **1C**

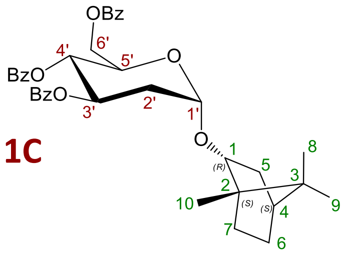

DQG-COSY

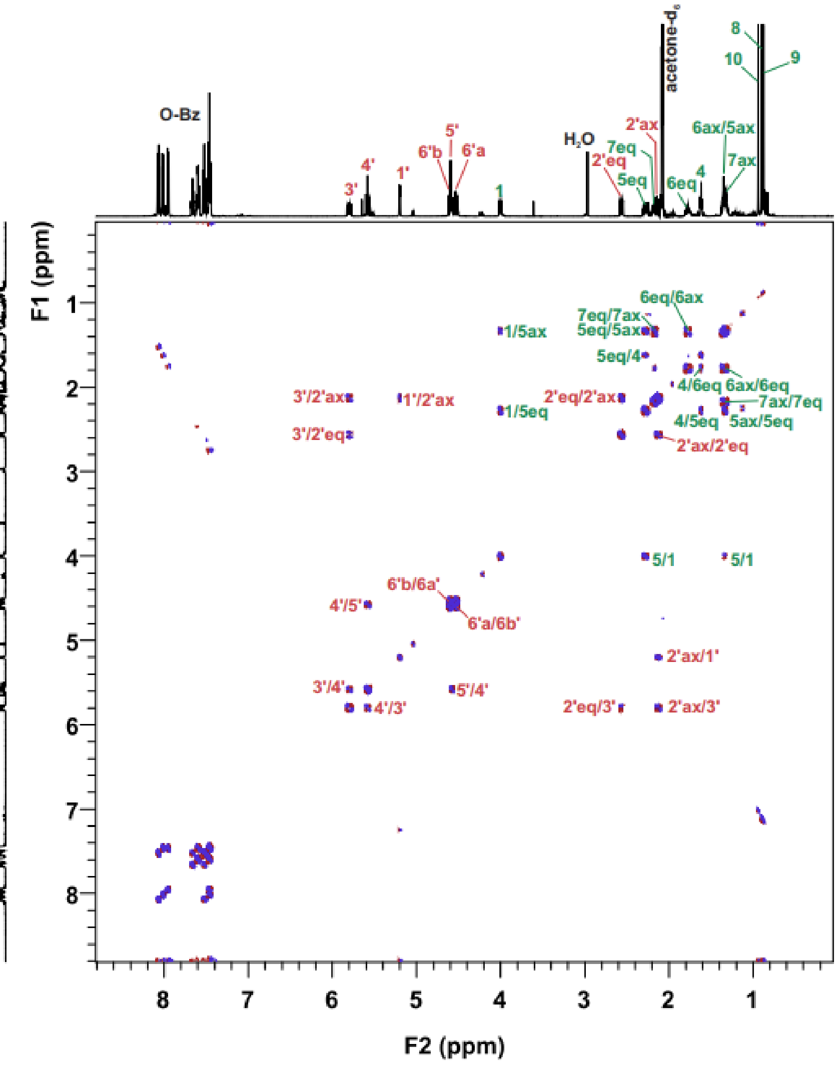

Figure.20. DQF-COSY spectrum for **1C**

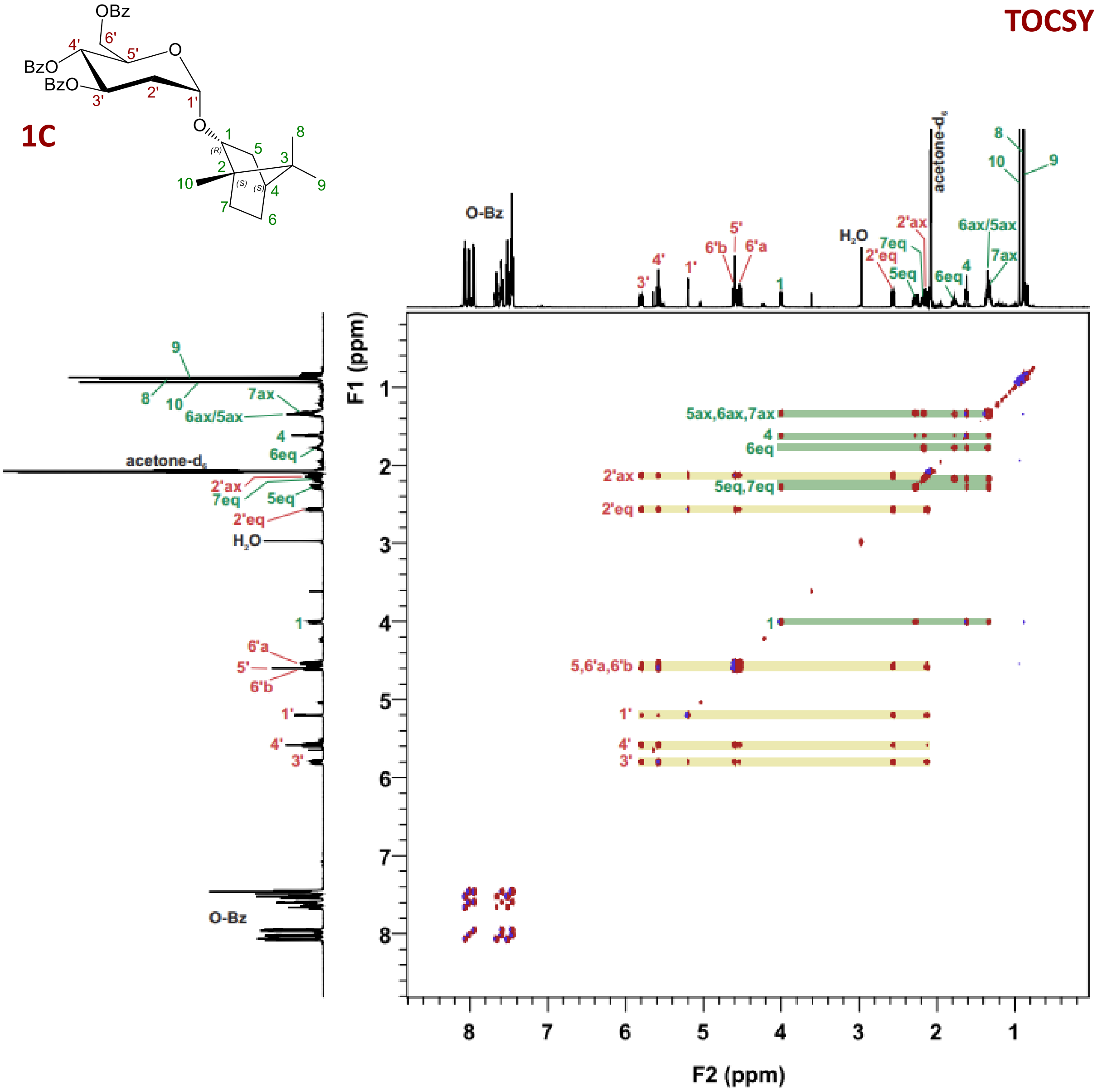

Figure.21. TOCSY spectrum for **1C**

HSQC

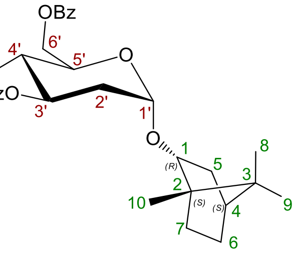

1C

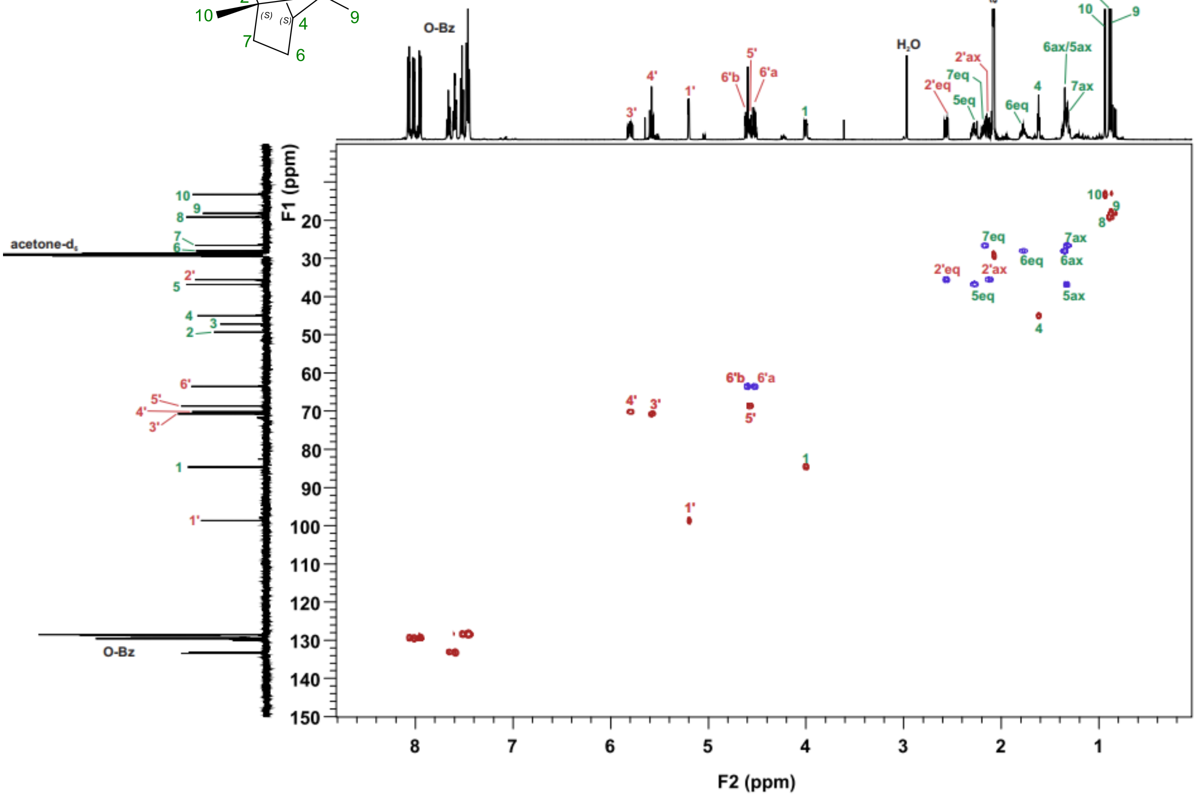

Figure.22. HSQC spectrum for **1C**

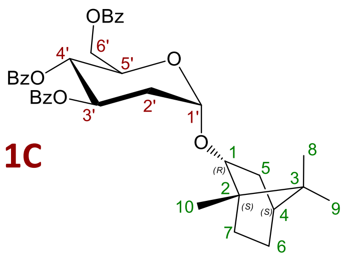

1C

nd-HSQC

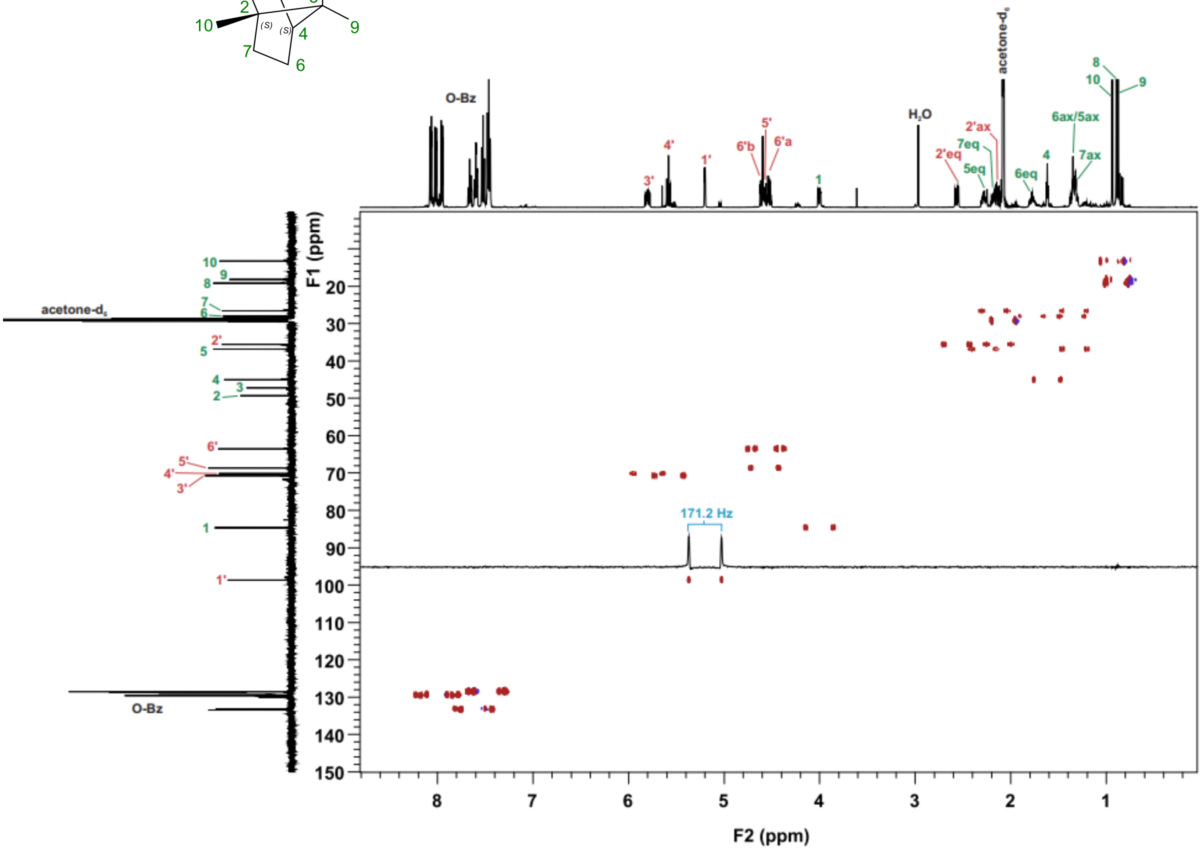

Figure.23. HSQC (without decoupling) spectrum for **1C**

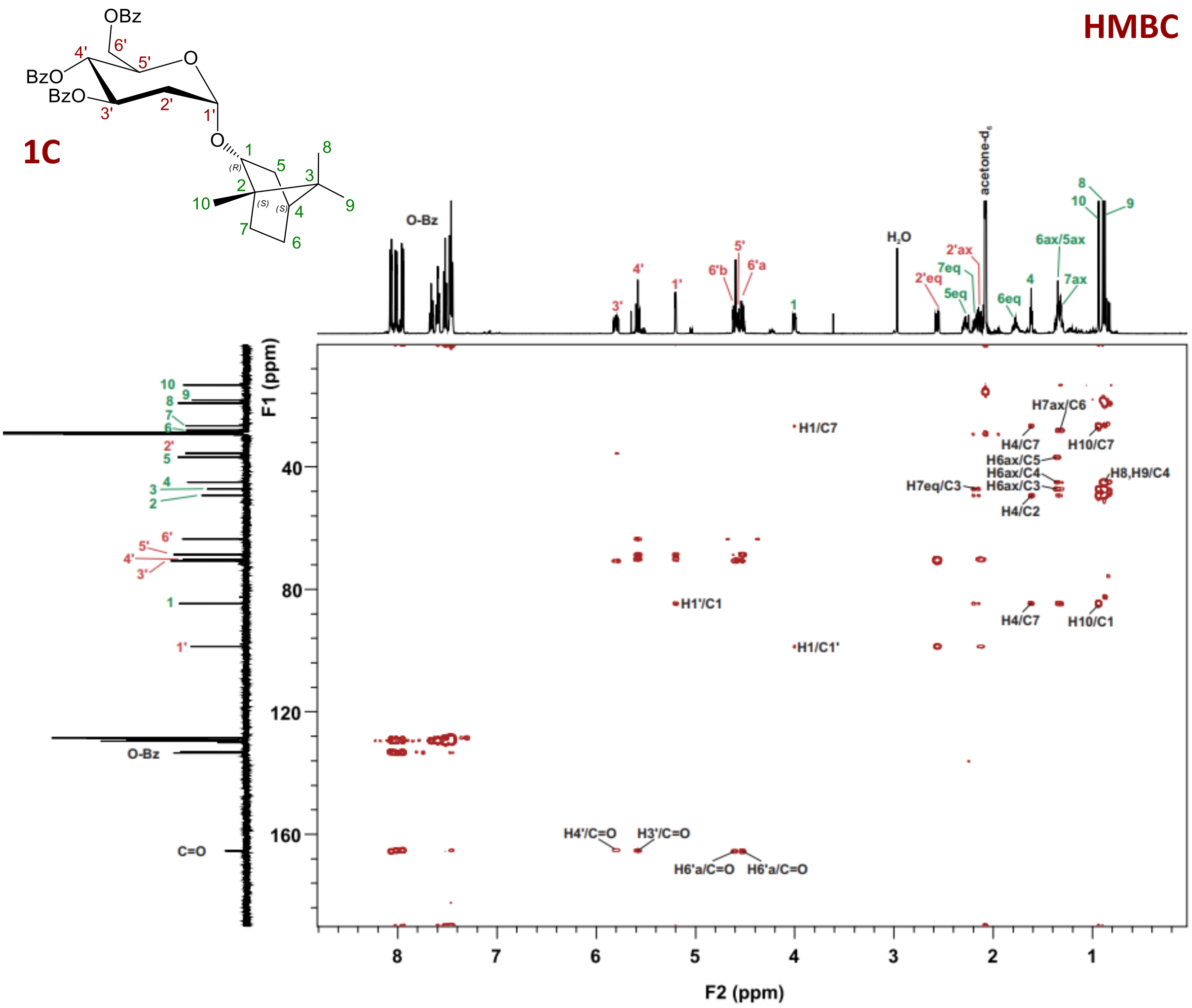

Figure.24. HMBC spectrum for **1C**

Table.9. Diagnostic ROE to proton for **1C**

2D ROESY NMR Data for **1C**	
position	diagnostic **ROE to proton**
**Aglycone Unit**	
**1**	5en, 9 ,10
**5en**	1, 9
**6en**	8
**9**	1
**10**	1
**Probe Moiety**	
**1'**	1, 10
**5'**	5ex

ROESY

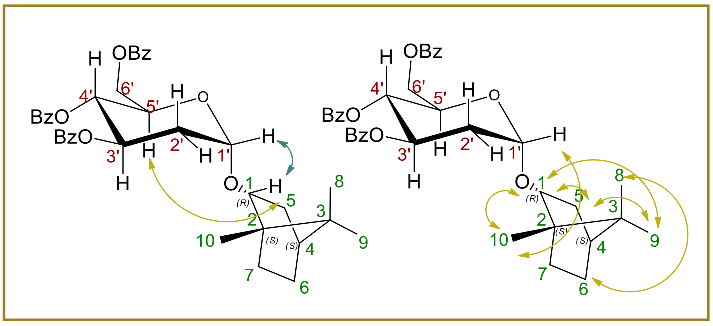

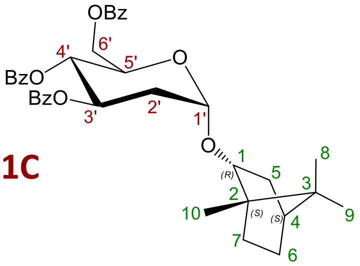

1C

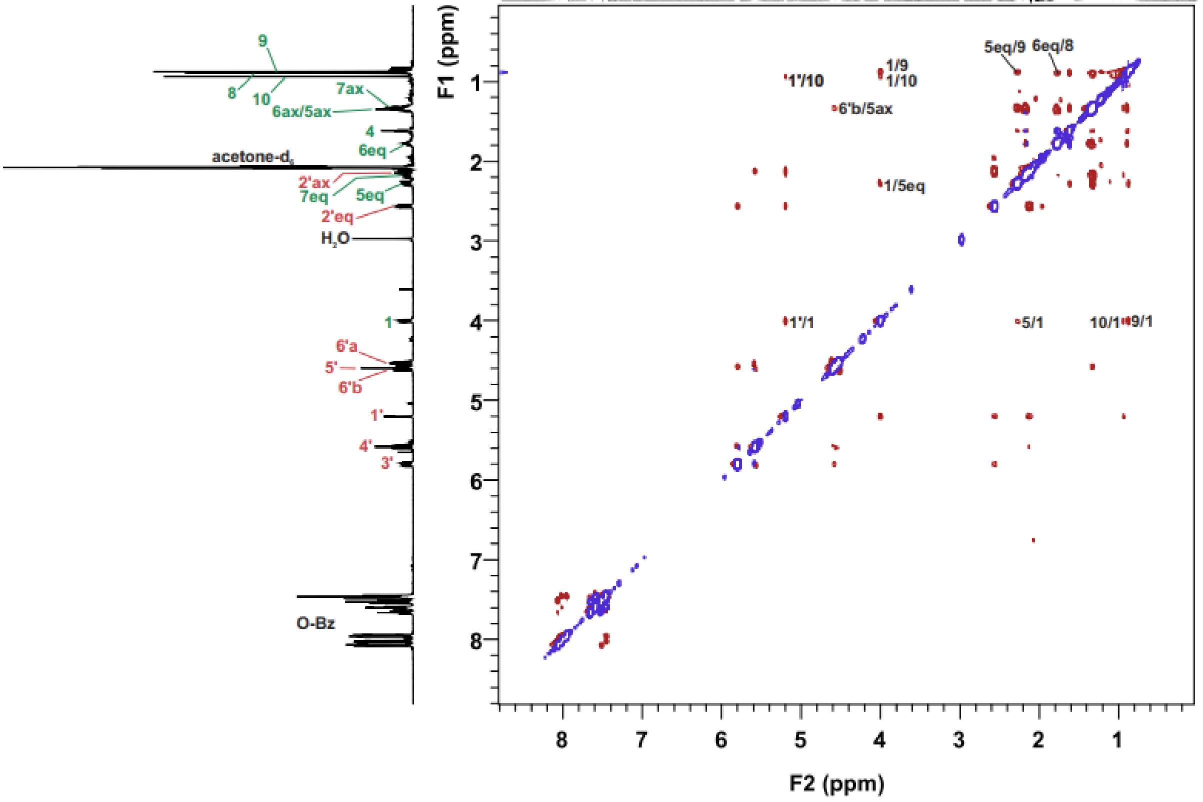

Figure.25. ROESY spectrum for **1C**

Table 10. Chemical shifts and coupling constants for molecule **2A** (^{1}H NMR)

^{1}H NMR Data for **2A**		
position	^{1}H δ (ppm)	$J_{H,H}$ (Hz)
**Aglycone Unit**		
**1**	3.47	10.7 (**2**), 10.7 (**6ax**), 4.2 (**6eq**)
**2**	1.25	10.7 (**1**), 11.4 (**3ax**), 2.9 (**3eq**), 2.9 (**7**)
**3ax**	1.02	11.4 (**2**), 12.7 (**3eq**), 12.7 (**4ax**), 3.1 (**4eq**)
**3eq**	1.65	2.9 (**2**), 12.7 (**3ax**), (**4ax**)*, (**4eq**)*
**4ax**	0.87	12.7 (**3ax**), (**3eq**)*, 12.5 (**4eq**), (**5**)*
**4eq**	1.68	3.1 (**3ax**), (**3eq**)* 12.5 (**4ax**), (**5**)*
**5**	1.39	(**4ax**)*, (**4eq**)*, 11.8 (**6ax**), 2.9 (**6eq**), 7.0 (**10**)
**6ax**	0.79	10.7 (**1**). 11.8 (**5**), (**6eq**)*
**6eq**	2.19	4.2 (**1**), 2.9 (**5**), (**6ax**)*
**7**	2.28	2.9 (**2**), 7.0 (**8**), 7.0 (**9**)
**8**	0.77	7.0 (**7**)
**9**	0.91	7.0 (**7**)
**10**	0.94	7.0 (**5**)
**Probe Moiety**		
**1'**	5.20	3.4 (**2’ax**), ~0.0 (**2’eq**),
**2’ax**	1.68	3.4 (**1’**), 12.3 (**2’eq**), 11.9 (**3’**)
**2’eq**	2.25	~0.0 (**1’**), 12.3 (**2’ax**), 5.0 (**3’**)
**3’**	3.94	11.9 (**2’ax**), 5.0 (**2’eq**), 9.3 (**4’**)
**4'**	3.57	9.3 (**3’**), 9.3 (**5’**)
**5'**	3.81	9.3 (**4’**), 2.8 (**6’a**), 4.2 (**6’b**)
**6'a**	3.70	2.8 (**5’**), 10.8 (**6’b**)
**6’b**	3.82	4.2 (**5’**), 10.8 (**6’a**)
**O-Bn-CH$_2$ (6H)**	4.54-4.76	
**O-Bn-ar (15H)**	7.26-7.43	
*These coupling constants could not be measured. Signal pattern remains partially unclear due to serve signal overlap and higher order effects		

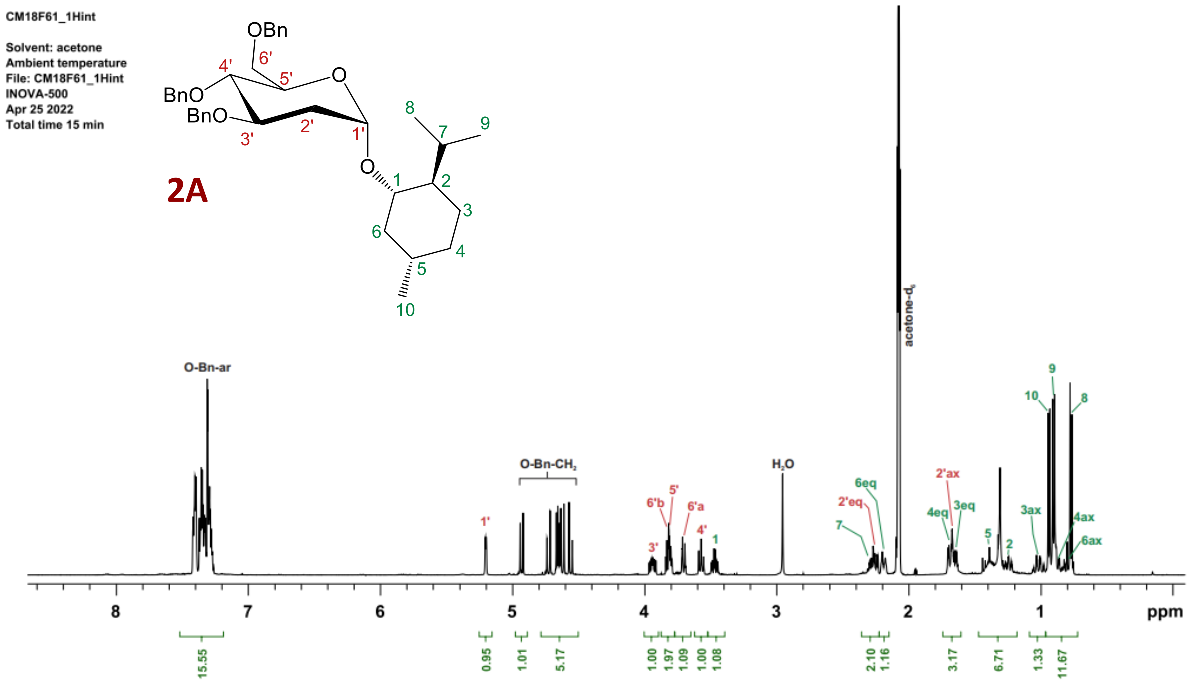

Figure.26. ^{1}H NMR spectrum for **2A**

Table 11. Chemical shifts for molecule **2A** (^{13}C NMR)

^{13}C NMR

^{13}C NMR Data for **2A**		
position		^{13}C δ (ppm)
**Aglycone Unit**		
**1**	CH	73.9
**2**	CH	48.2
**3**	CH_2	22.7
**4**	CH_2	34.4
**5**	CH	31.2
**6**	CH_2	39.5
**7**	CH	25.1
**8**	CH_3	15.2
**9**	CH_3	20.7
**10**	CH_3	21.8
**Probe Moiety**		
**1'**	anomeric carbon	92.8
**2'**		35.9
**3'**		77.5
**4'**		78.6
**5'**		71.8
**6'**		69.5
**O-Bn-CH_2**		70.9<br>73.0<br>74.6
**O-Bn-ar**	18 aromatic carbons	127.3 – 128.2

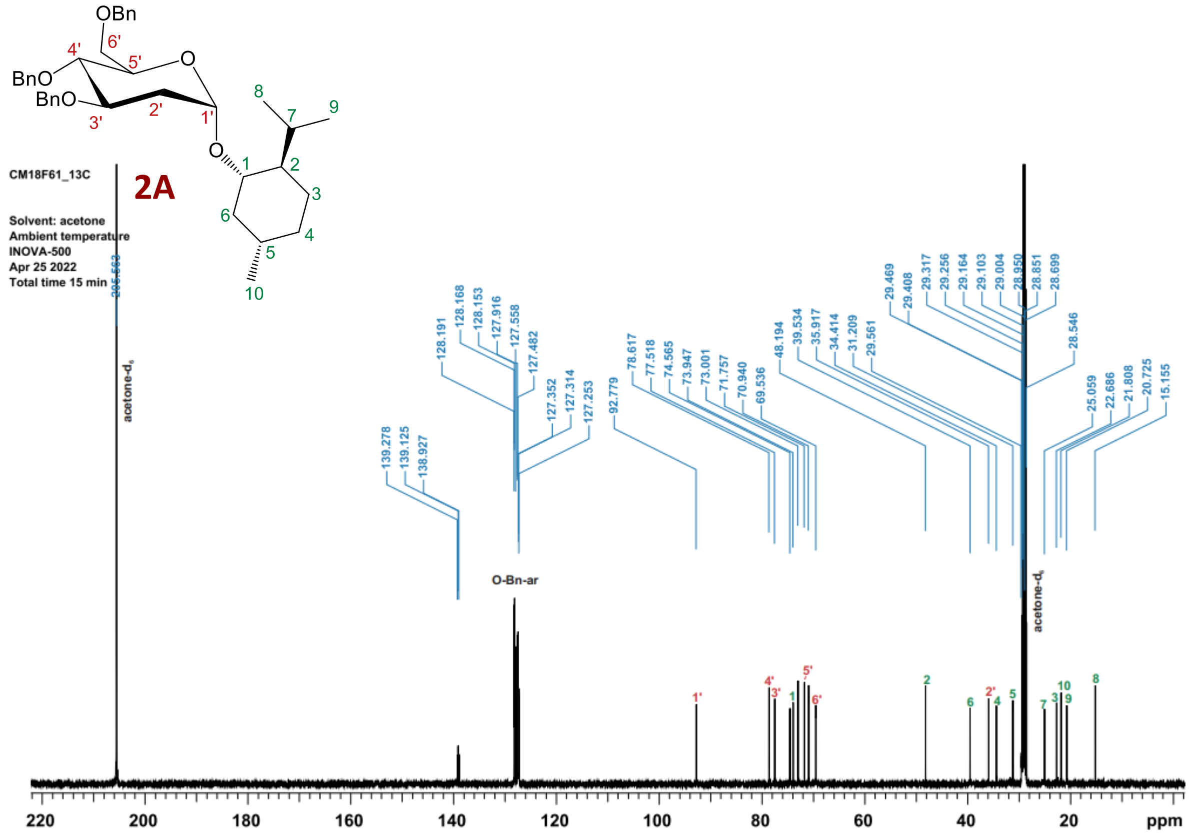

Figure.27.^{13}C NMR spectrum for **2A**

DQF-COSY

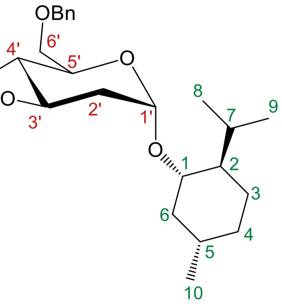

2A

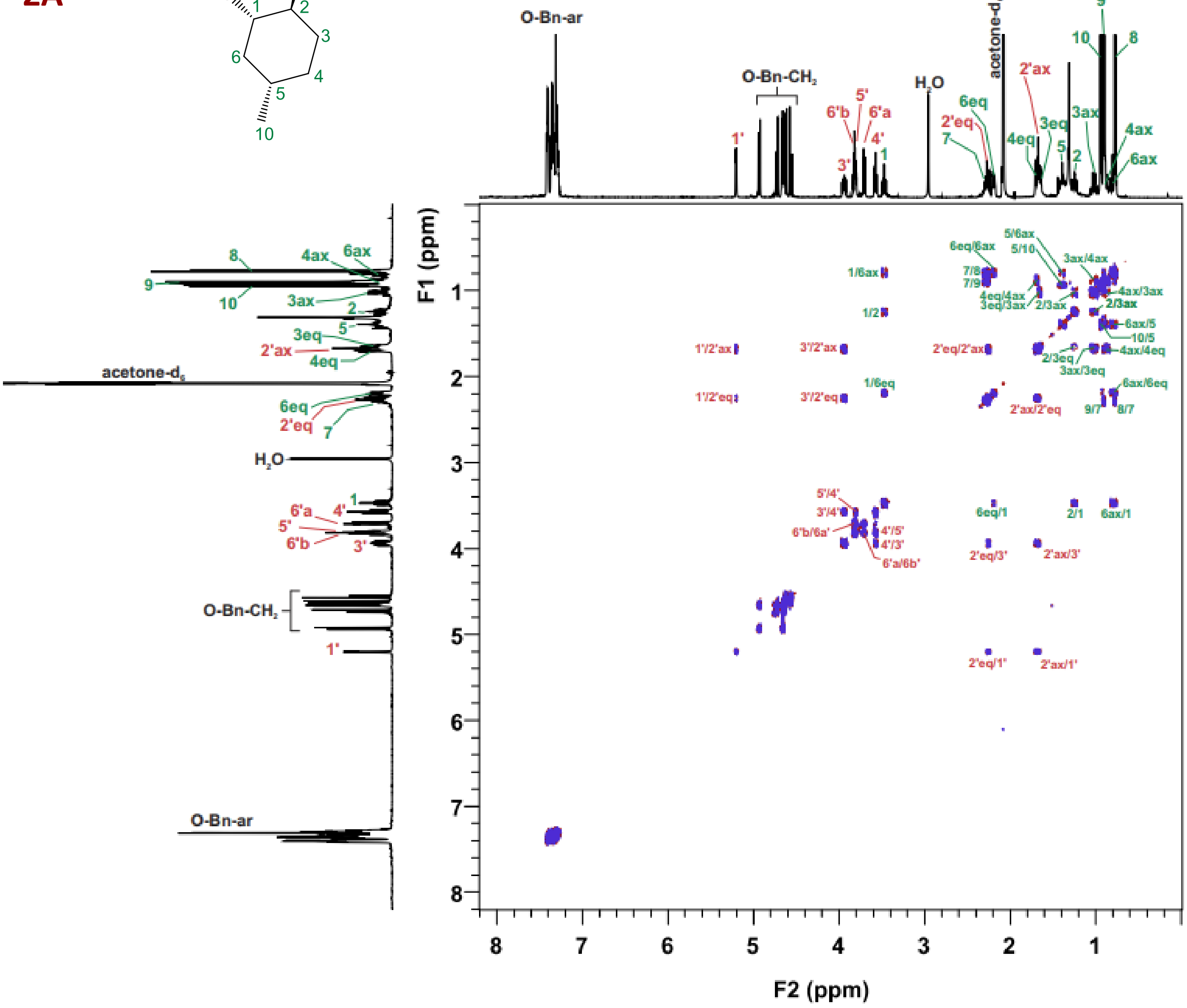

Figure.28.DQF-COSY spectrum for **2A**

TOCSY

2A

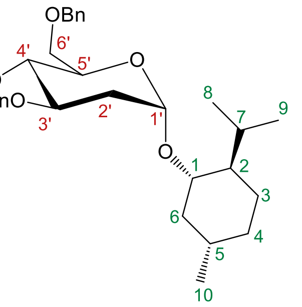

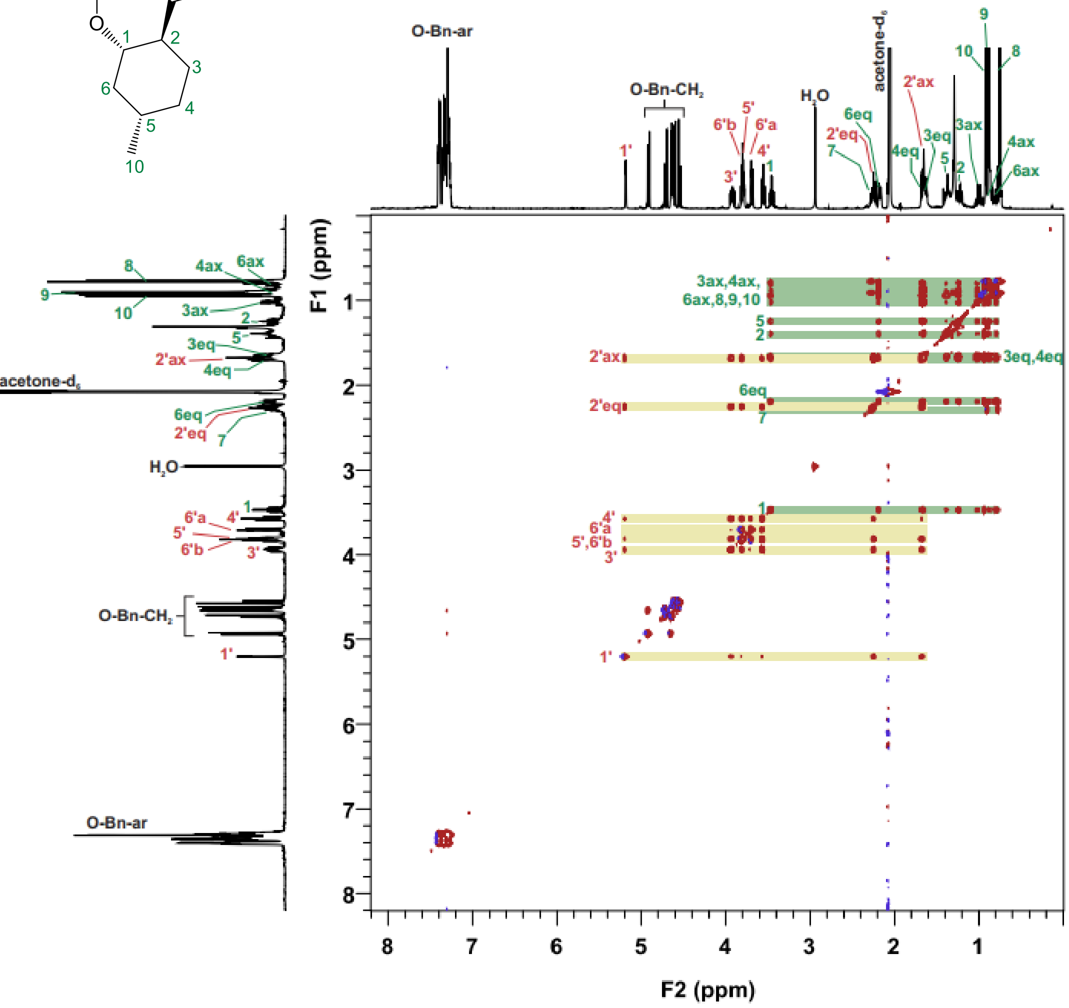

Figure.29. TOCSY spectrum for **2A**

HSQC

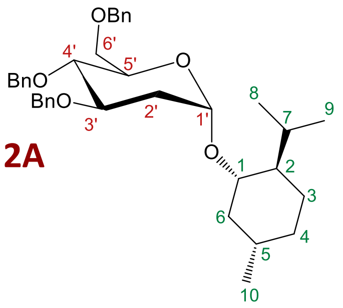

2A

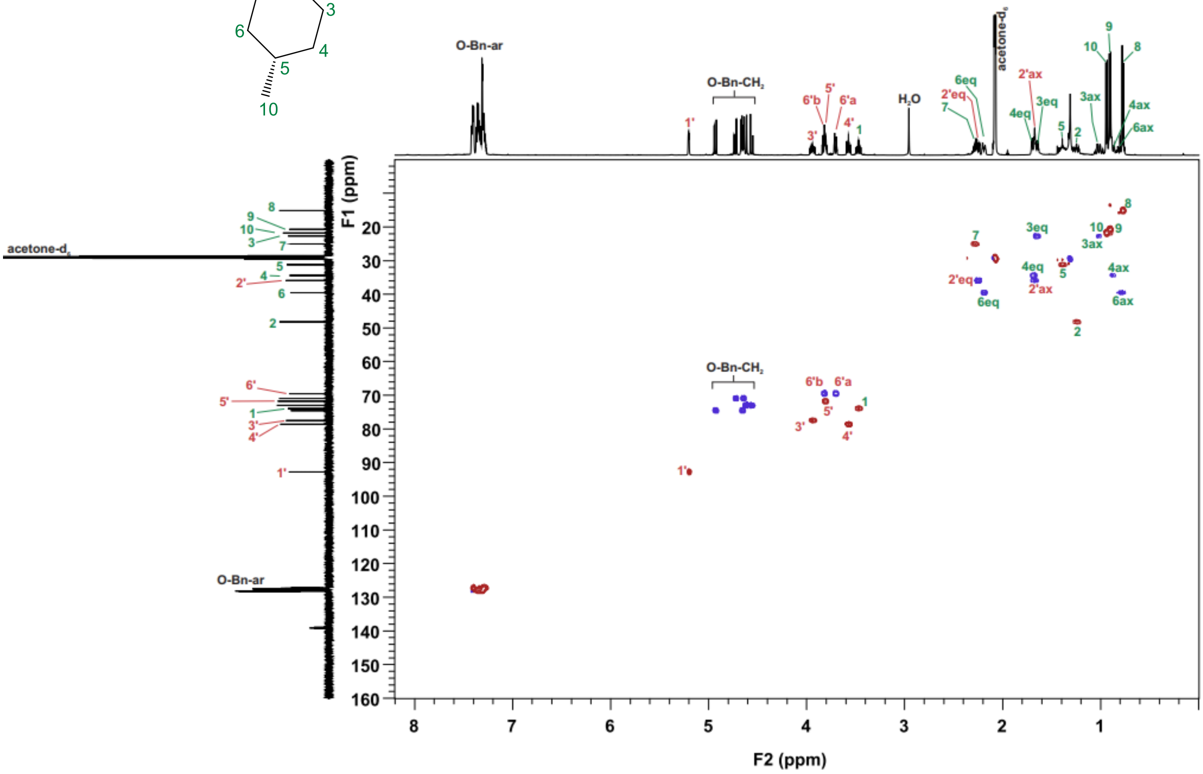

Figure.30. HSQC spectrum for **2A**

nd-HSQC

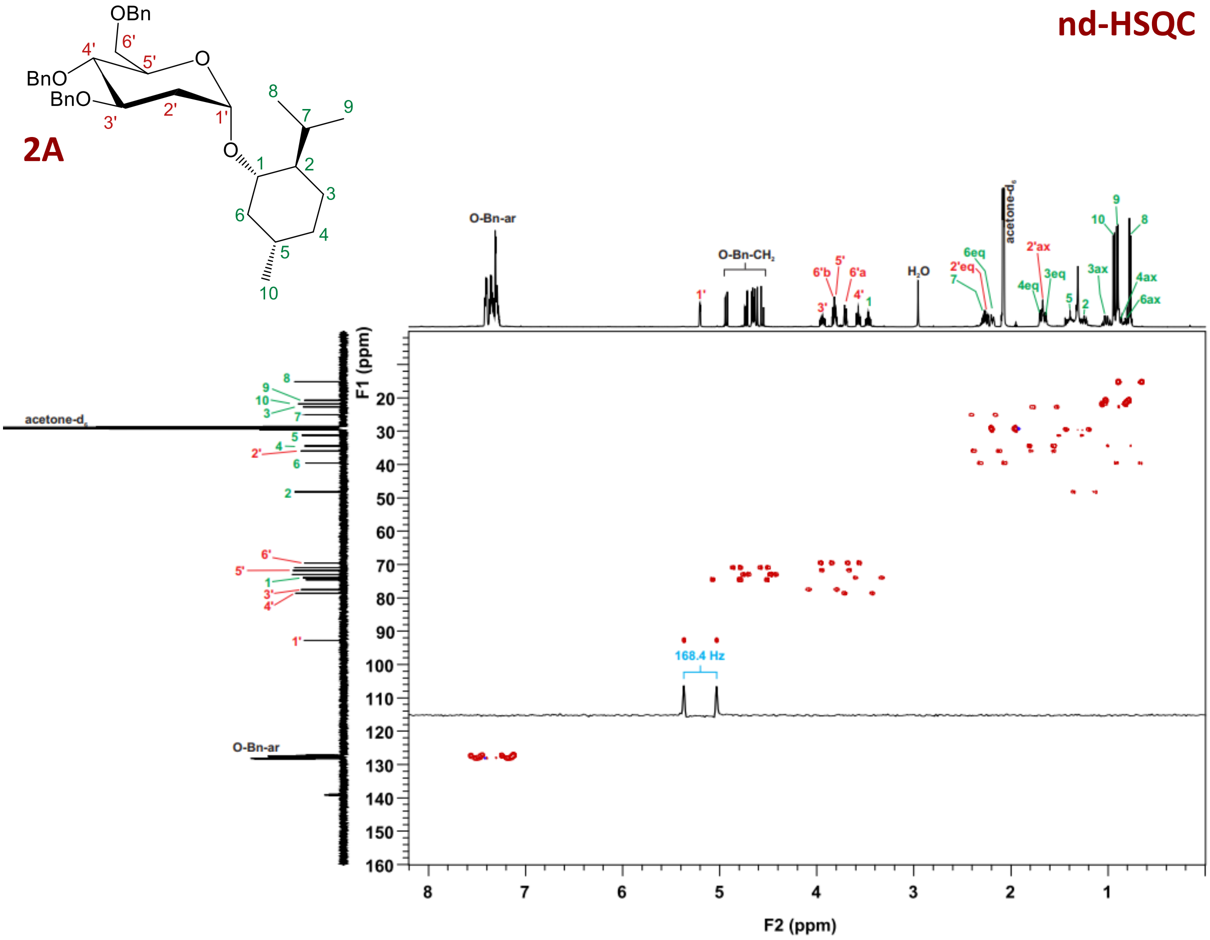

Figure.31. nd-HSQC spectrum for **2A**

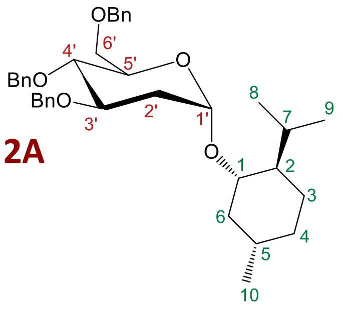

2A

HMBC

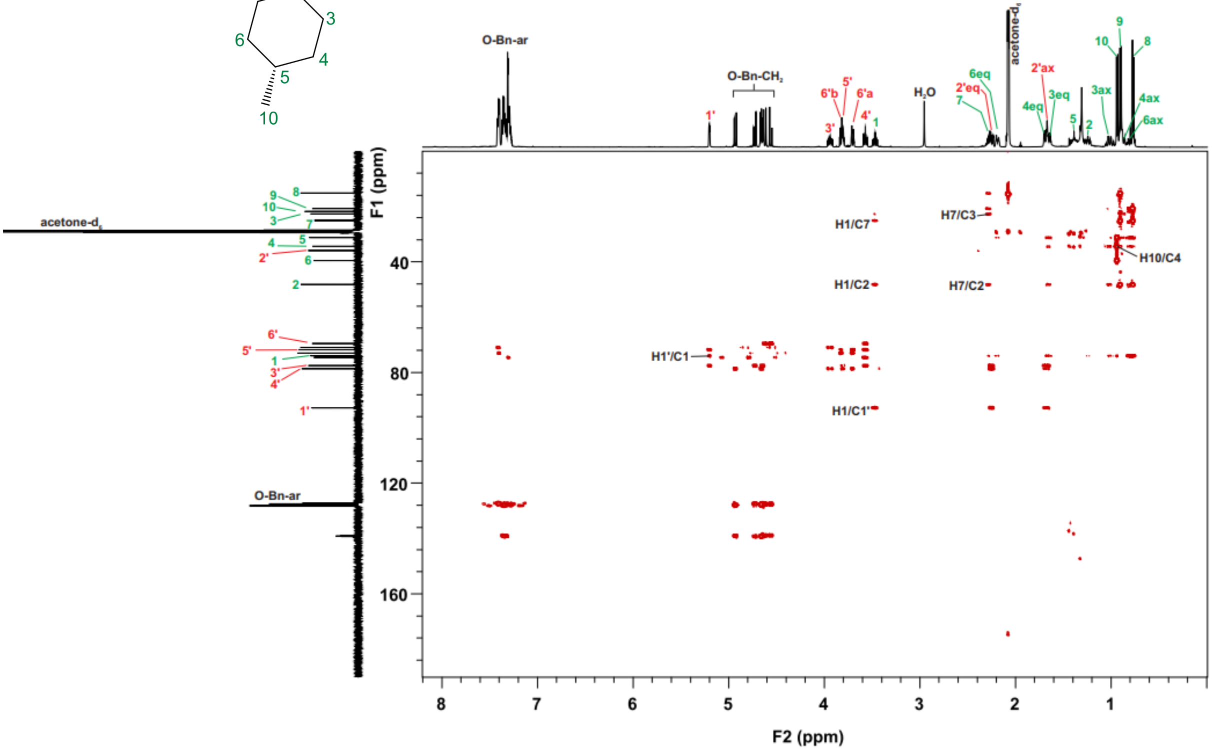

Figure.32. HMBC spectrum for **2A**

Table.12. Diagnostic ROE to proton for **2A**

2D ROESY NMR Data for **2A**	
position	diagnostic **ROE to proton**
**Aglycone Unit**	
**1**	3ax, 6eq, 8
**Probe Moiety**	
**1'**	1, 6eq
**4'**	2'ax
**5'**	7, 8
**6'a**	8

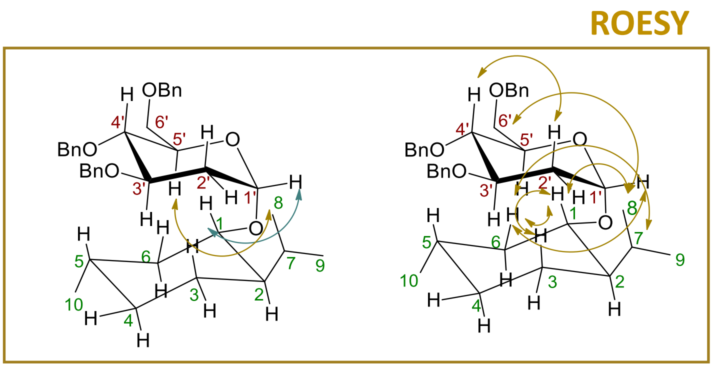

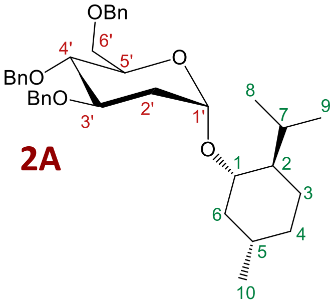

2A

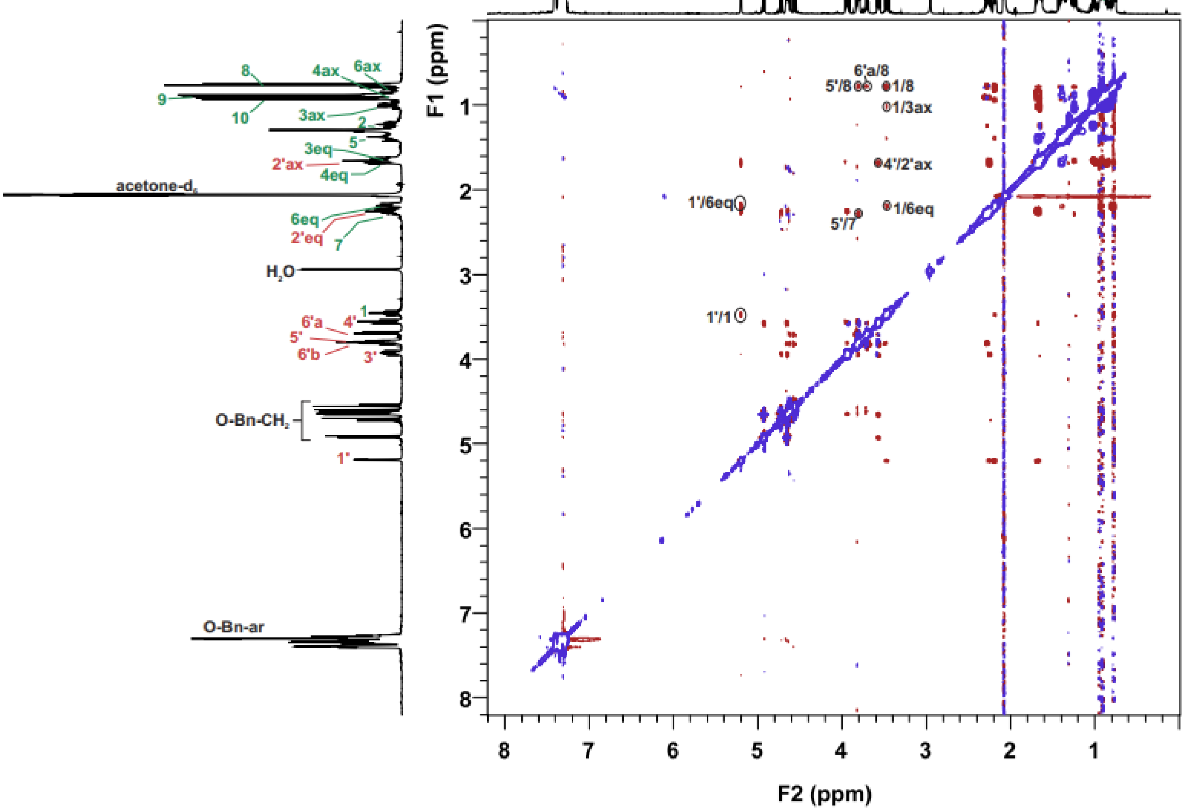

Figure.33. ROESY spectrum for **2A**

Table 13. Chemical shifts and coupling constants for molecule **2B** (^{1}H NMR)

^{1}H NMR Data for **2B**		
position	^{1}H δ (ppm)	$J_{H,H}$ (Hz)
**Aglycone Unit**		
**1**	3.64	10.7 (**2**), 10.7 (**6ax**), 4.2 (**6eq**)
**2**	1.32	10.7 (**1**), 11.8 (**3ax**), (**3eq**)*, 2.4 (**7**)
**3ax**	0.90	11.8 (**2**), 12.7 (**3eq**), 12.7 (**4ax**), (**4eq**)*
**3eq**	1.53	(**2**)*, 12.7 (**3ax**), (**4ax**)*, (**4eq**)*
**4ax**	0.72	12.7 (**3ax**), (**3eq**)*, 13.0 (**4eq**), 12.7 (**5**)
**4eq**	1.50	(**3ax**)*, (**3eq**)*, 13.0 (**4ax**), (**5**)*
**5**	1.10	12.7 (4ax), (**4eq**)*, (**6ax**)*, (**6eq**)*, 6.9 (**10**)
**6ax**	0.82	10.7 (**1**). (**5**)* 11.9 (**6eq**)
**6eq**	1.98	4.2 (**1**), (**5**)*, 11.9 (**6ax**)
**7**	2.51	2.4 (**2**), 6.9 (**8**), 6.9 (**9**)
**8**	0.89	6.9 (**7**)
**9**	0.88	6.9 (**7**)
**10**	0.81	6.9 (**5**)
**Probe Moiety**		
**1'**	5.26	~0.0 (**2'**)
**2'**	5.66	~0.0 (**1'**), 10.3 (**3'**), 2.3 (**4'**)
**3'**	5.94	10.3 (**2'**), ~0.0 (**4'**)
**4'**	4.32	2.3 (**2'**), ~0.0 (**3'**), 11.3 (**5'**)
**5'**	4.24	11.3 (**4'**), 1.8 (**6'a**), 4.1 (**6'b**)
**6'a**	3.80	1.8 (**5'**), 10.8 (**6'b**)
**6'b**	3.86	4.1 (**5'**), 10.8 (**6'a**)
**O-Bn-CH_2 (4H)**	4.30-4.53	
**O-Bn-ar (15H)**	7.05-7.32	
*These coupling constants could not be measured. Signal pattern remains partially unclear due to serve signal overlap and higher order effects		

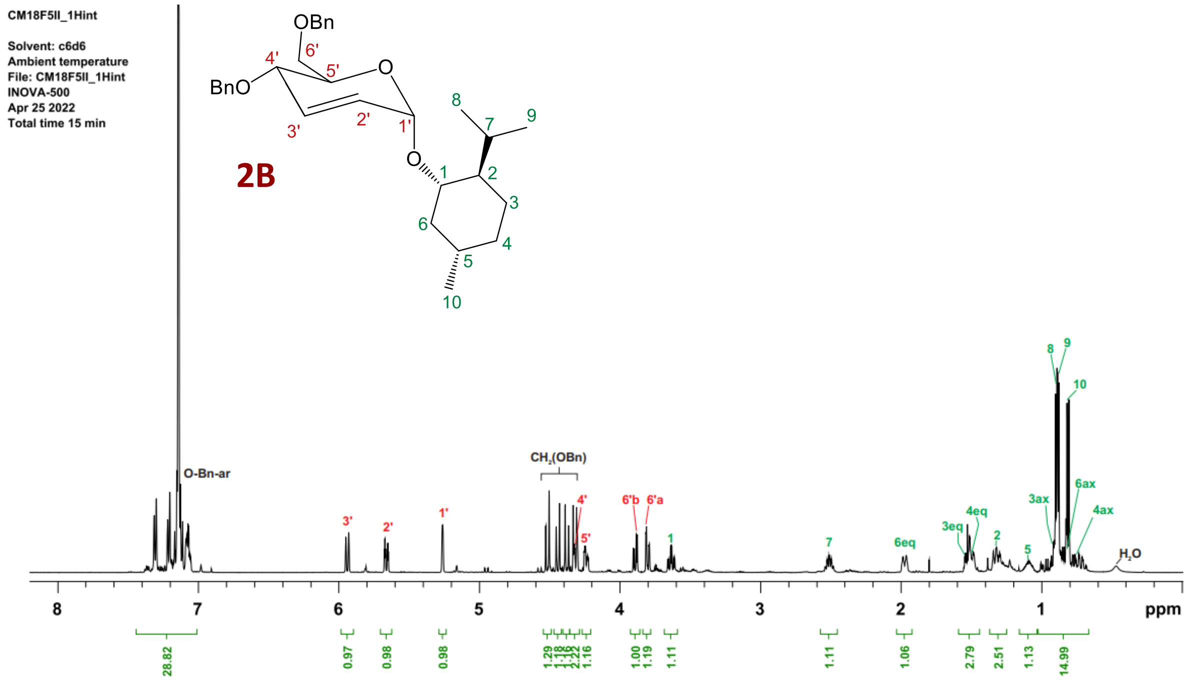

Figure.34. ^{1}H NMR spectrum for **2B**

Table 14. Chemical shifts for molecule **2B** (^{13}C NMR)

^{13}C NMR

^{13}C NMR<br>Data for **benzyl UNSATURATED BOND 2’ = 3’ (alpha) 2DG (+)-menthol**		
position		^{13}C δ (ppm)
**Aglycone Unit**		
**1**	CH	74.8
**2**	CH	48.2
**3**	CH_2	23.0
**4**	CH_2	34.5
**5**	CH	31.2
**6**	CH_2	40.0
**7**	CH	25.2
**8**	CH_3	15.8
**9**	CH_3	21.1
**10**	CH_3	22.2
**Probe Moiety**		
**1'**	anomeric carbon	90.3
**2'**		unidentified, hidden under the signal from the solvent (~127.7) HSQC
**3’**		130.3
**4'**		70.9
**5'**		70.6
**6'**		69.6
**O-Bn-CH₂**	4’-CH_2(OBn)<br>6’-CH2(OBn)	70.3<br>73.3
**O-Bn-ar**	18 aromatic carbons	127.3 – 128.2

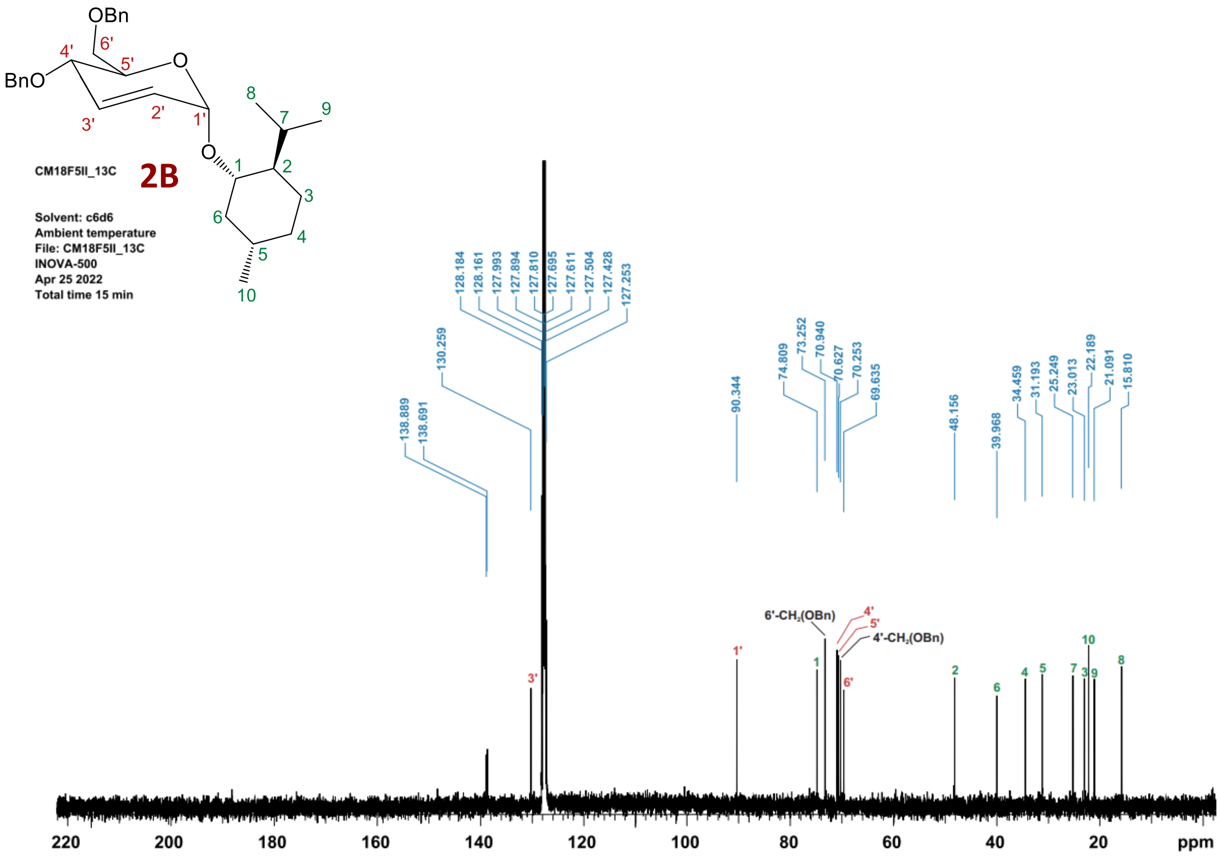

Figure.35. ^{13}C NMR spectrum for **2B**

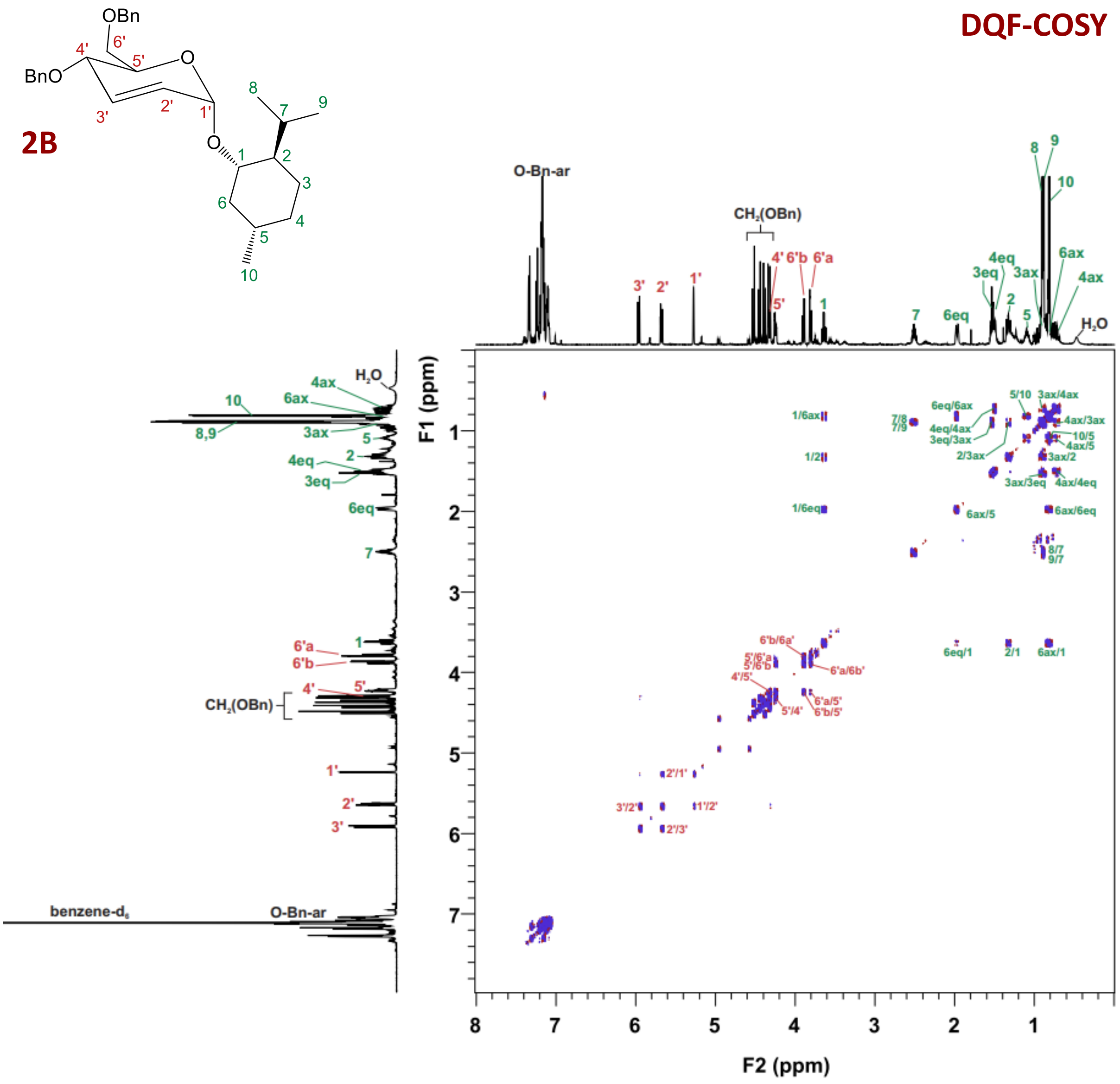

Figure.36. DQF-COSY spectrum for **2B**

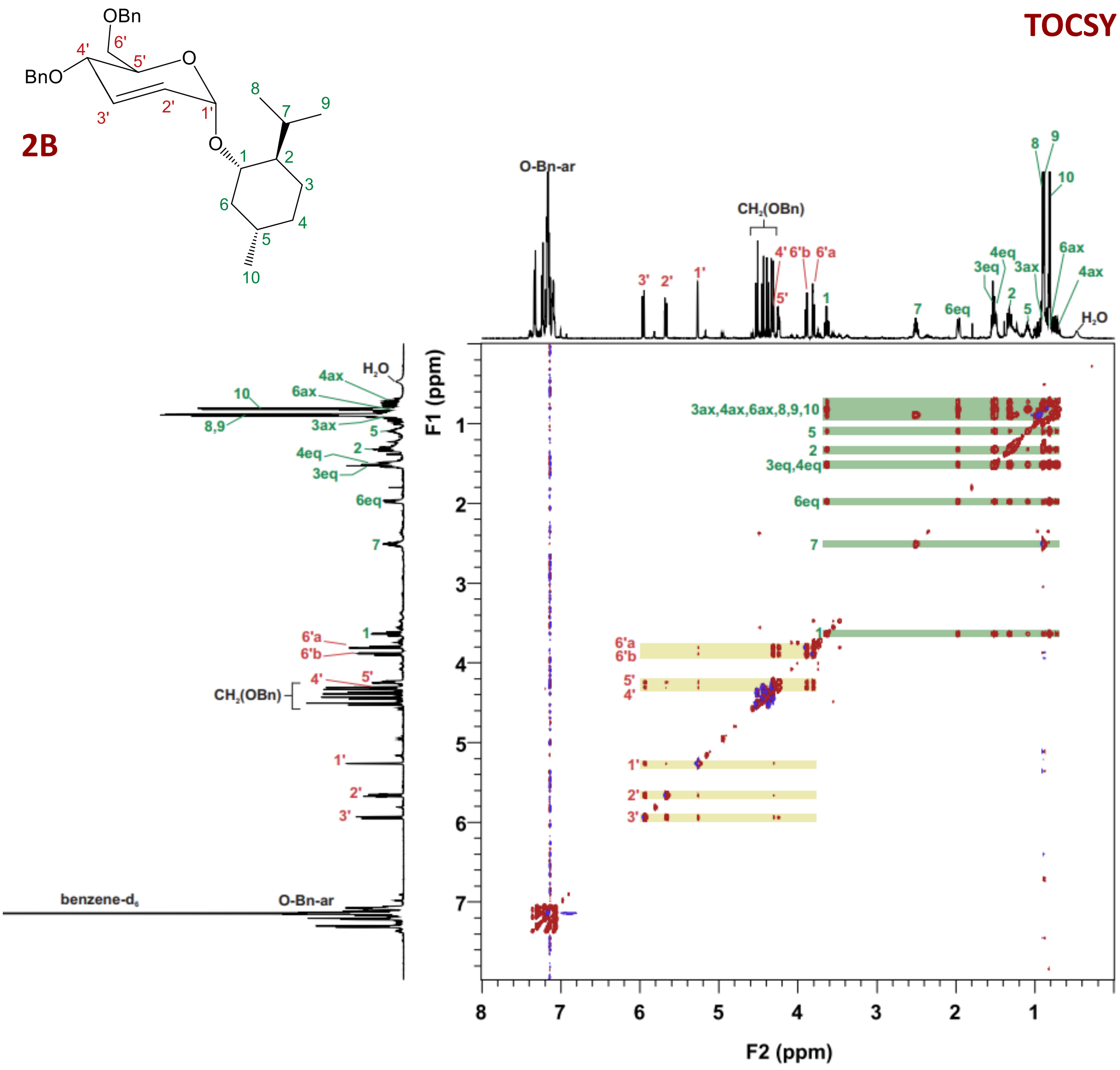

Figure.37. TOCSY spectrum for **2B**

HSQC

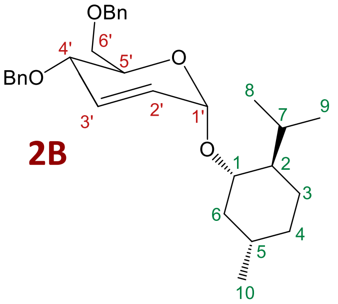

2B

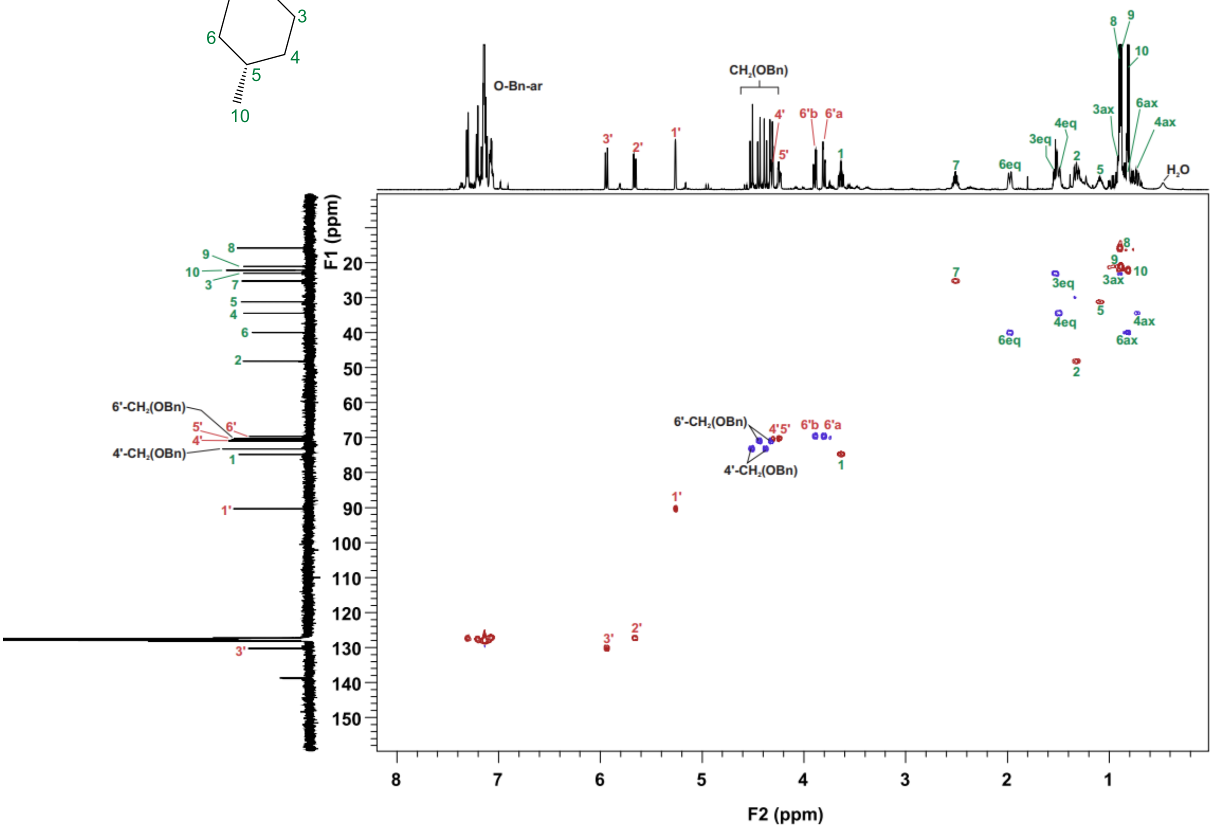

Figure.38. HSQC spectrum for **2B**

nd-HSQC

2B

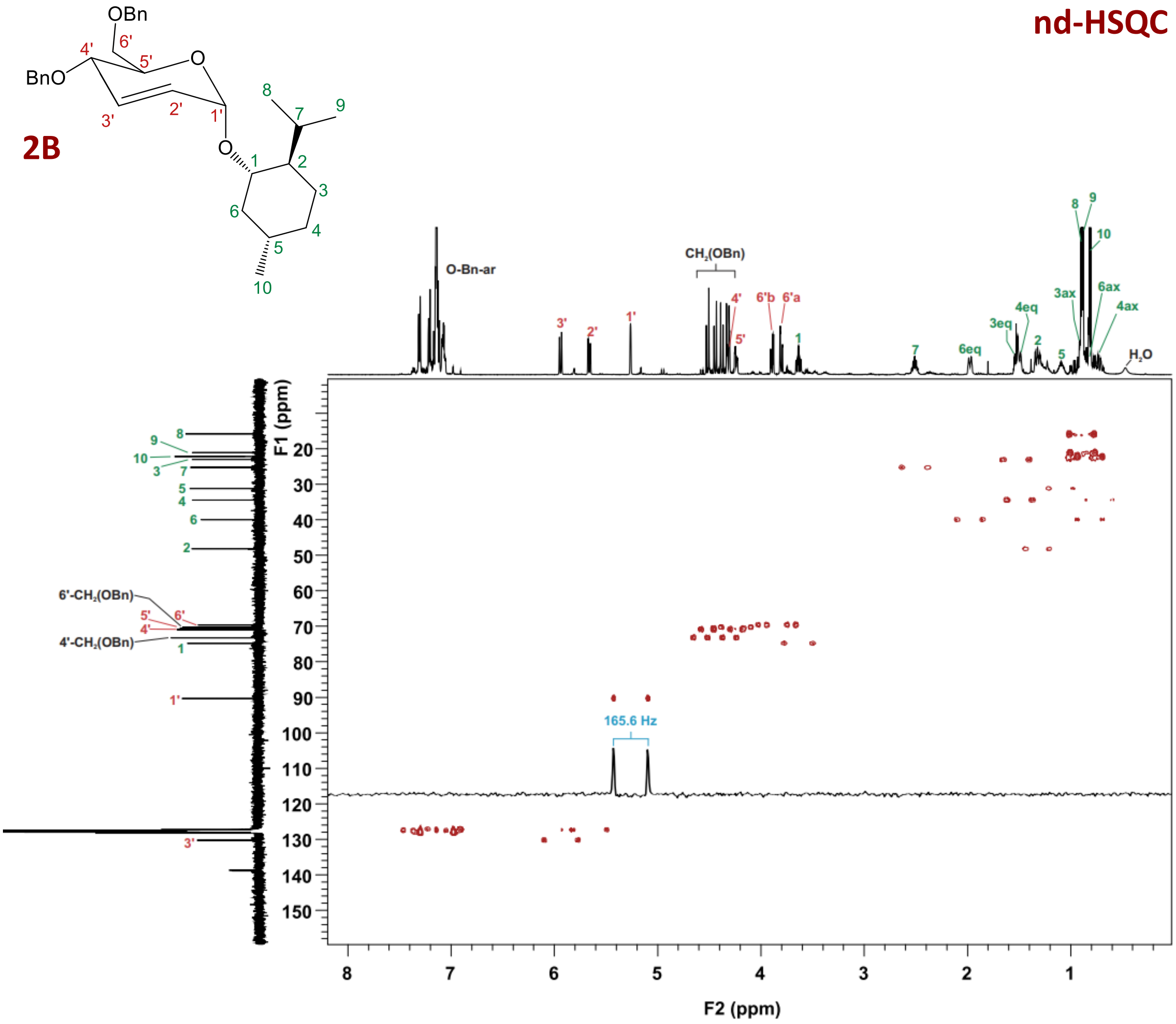

Figure.39. HSQC (without decoupling) spectrum for **2B**

HMBC

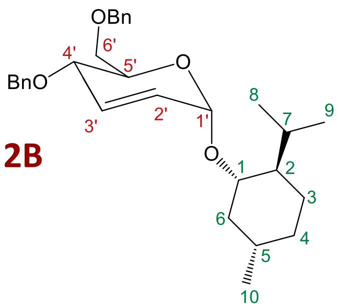

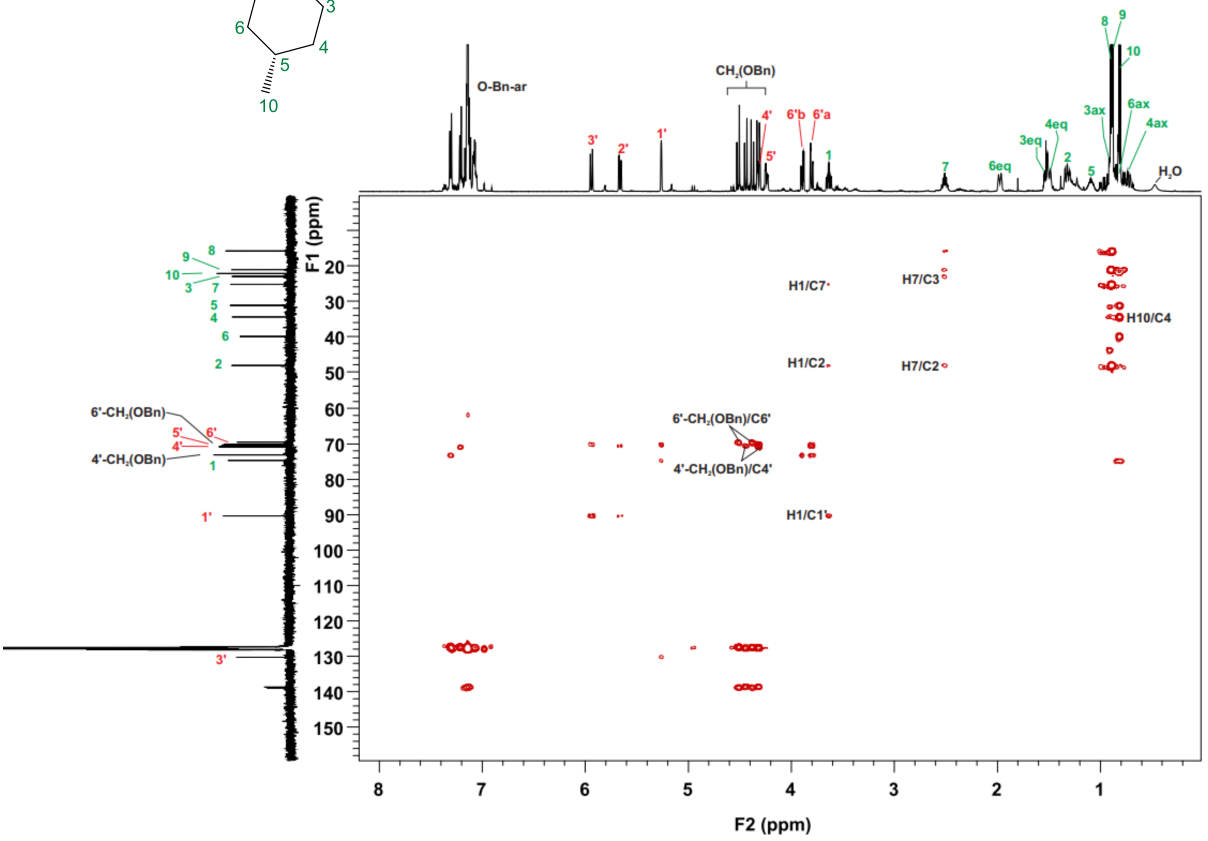

Figure.40. HMBC spectrum for **2B**

Table.15. Diagnostic ROE to proton for **2B**

2D ROESY NMR Data for **2B**	
position	diagnostic ROE to proton
**Aglycone Unit**	
**1**	5, 6eq
**Probe Moiety**	
**1'**	1, 6eq
**5'**	7

ROESY

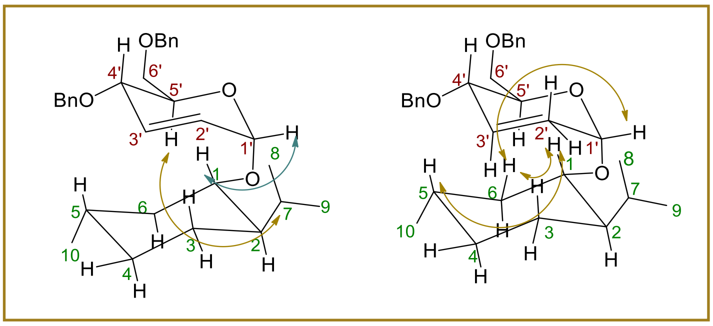

2B

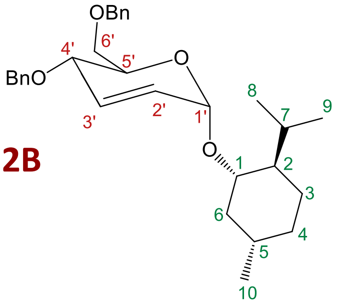

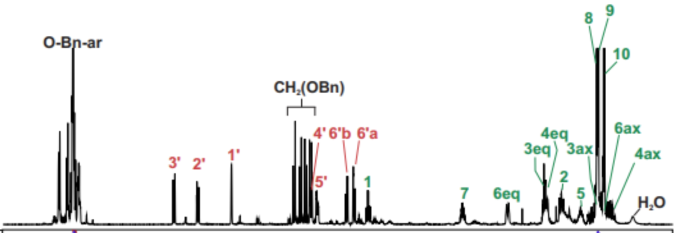

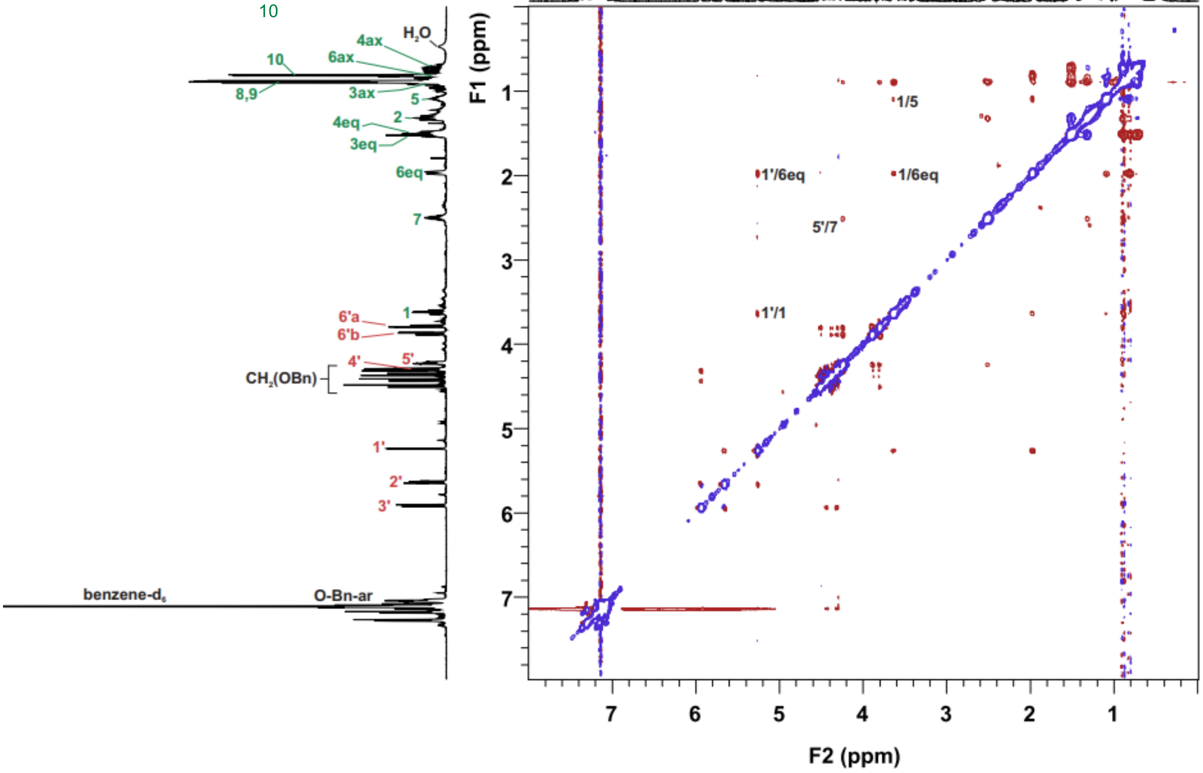

Figure.41. ROESY spectrum for **2B**

Table 16. Chemical shifts and coupling constants for molecule **2C** (^{1}H NMR)

^{1}H NMR Data for **2C**		
position	^{1}H δ (ppm)	$J_{H,H}$ (Hz)
**Aglycone Unit**		
**1**	1.11	6.1 (**2**)
**2**	3.70	6.1 (**1**), 6.1 (**3a**), 6.1 (**3b**)
**3a**	1.48	6.1 (**2**), 14.0 (**3b**), 7.5 (**4**)
**3b**	1.55	6.1 (**2**), 14.0 (**3a**), 7.5 (**4**)
**4**	0.91	7.5 (**3a**), 7.5 (**3b**)
**Probe Moiety**		
**1'**	5.10	3.6 (**2'ax**), ~0.8 (**2'eq**)
**2'ax**	1.64	3.6 (**1'**), 12.3 (**2'eq**), 12.3 (**3'**)
**2'eq**	2.27	~0.8 (**1'**), 12.3 (**2'ax**), 4.4 (**3'**)
**3'**	3.96	12.3 (**2'ax**), 4.4 (**2'eq**), 9.1 (**4'**)
**4'**	3.52	9.1 (**3'**), 9.1 (**5'**)
**5'**	3.84	9.1 (**4'**), (**6'a**)*, 4.5 (**6'b**)
**6'a**	3.37	(**5'**)*, 10.6 (**6'b**)
**6'b**	3.38	4.5 (**5'**), 10.6 (**6'a**)
**O-Bn-CH$_2$ (6H)**	4.54-4.96	
**O-Bn-ar (15H)**	7.26-7.42	
*These coupling constants could not be measured. Signal pattern remains partially unclear due to serve signal overlap and higher order effects		

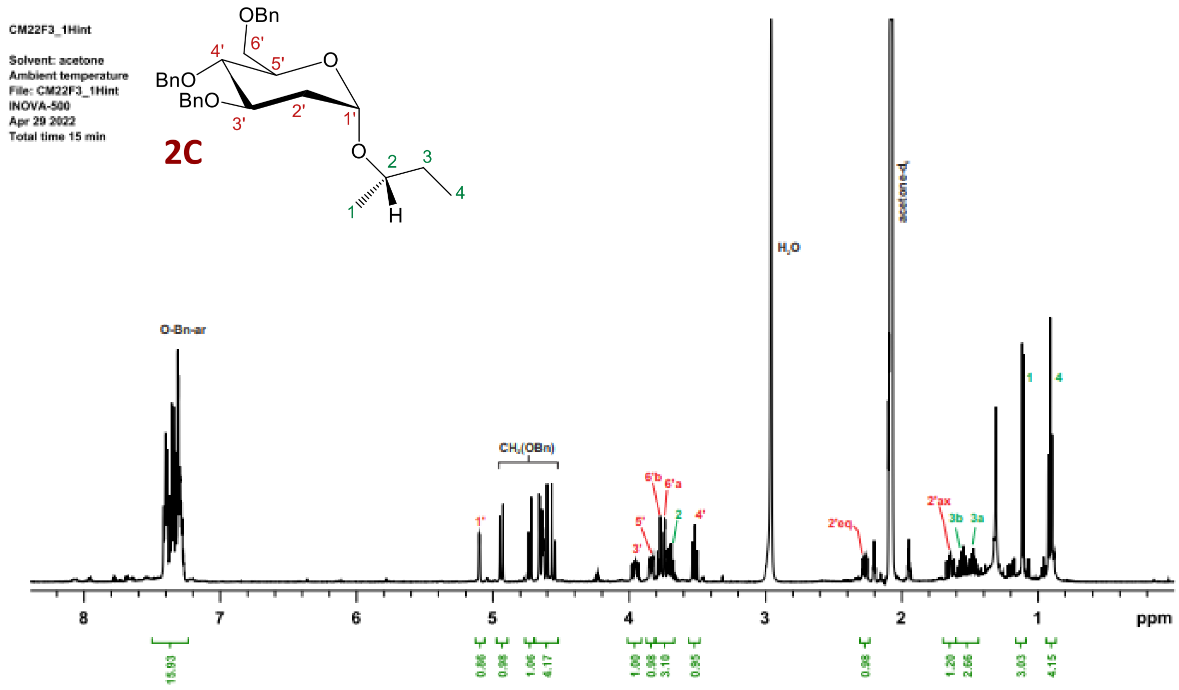

Figure.42. ^{1}H NMR spectrum for **2C**

Table 17. Chemical shifts for molecule **2C** (^{13}C NMR)

^{13}C NMR

^{13}C NMR Data for 2C		
position		^{13}C δ (ppm)
**Aglycone Unit**		
**1**	CH_3	17.7
**2**	CH	72.0
**3**	CH_2	29.9
**4**	CH_3	9.7
**Probe Moiety**		
**1'**	anomeric carbon	93.9
**2'**		35.7
**3'**		77.6
**4'**		78.6
**5'**		71.3
**6'**		69.6
**O-Bn-CH_2**		70.9<br>72.8<br>74.5
**O-Bn-ar**	18 aromatic carbons	127.2 – 128.2

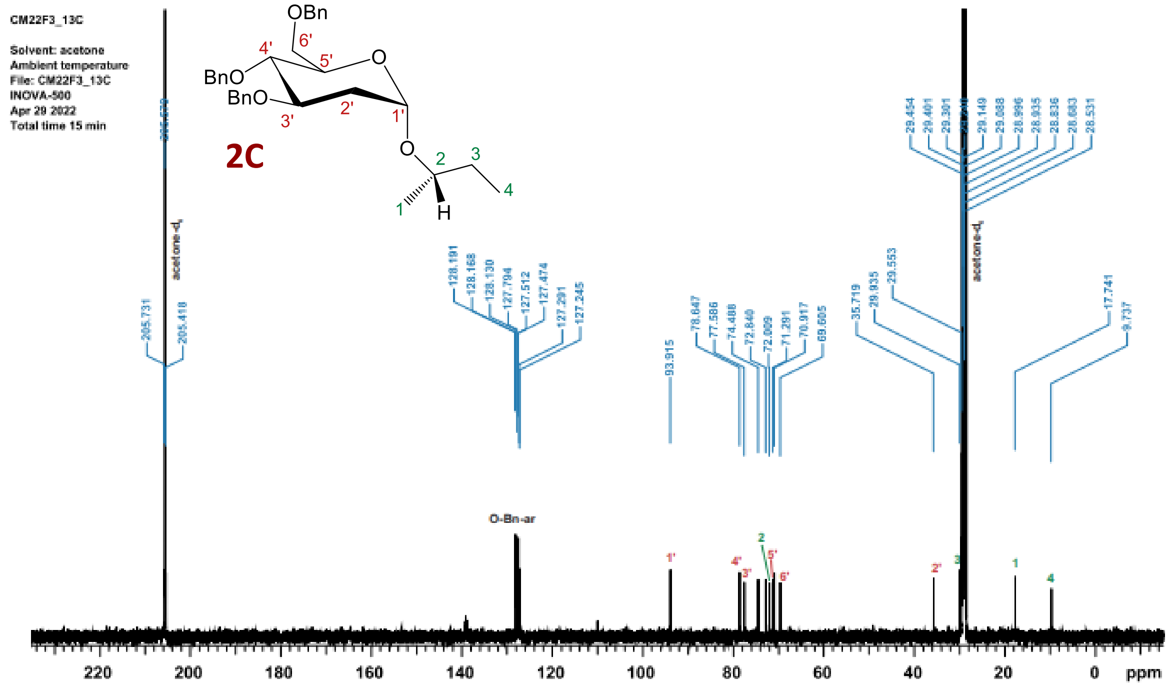

Figure.43. ^{13}C NMR spectrum for **2C**

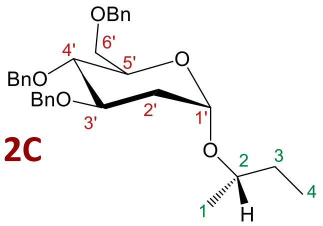

DQF-COSY

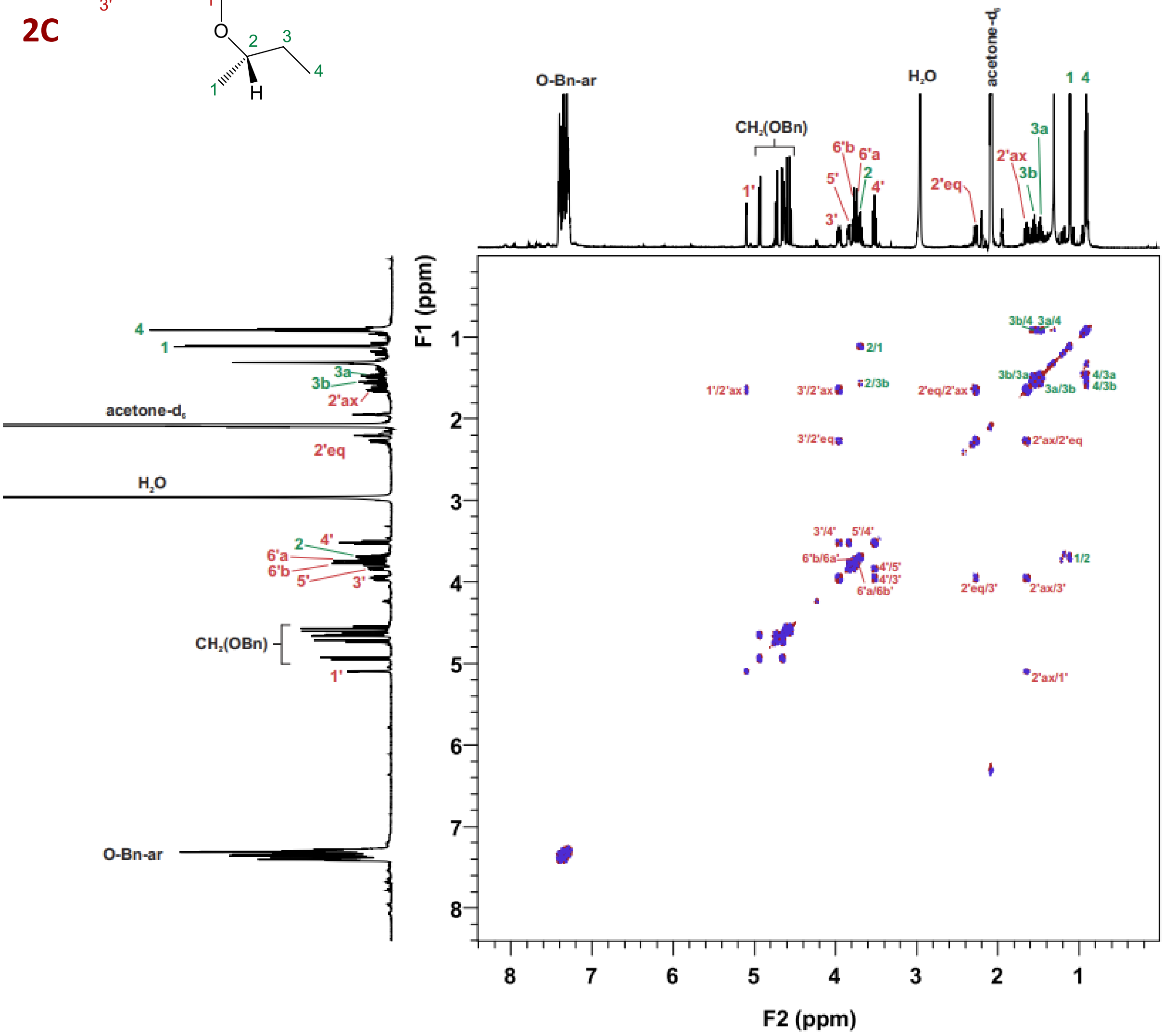

Figure.44. DQF-COSY spectrum for **2C**

TOCSY

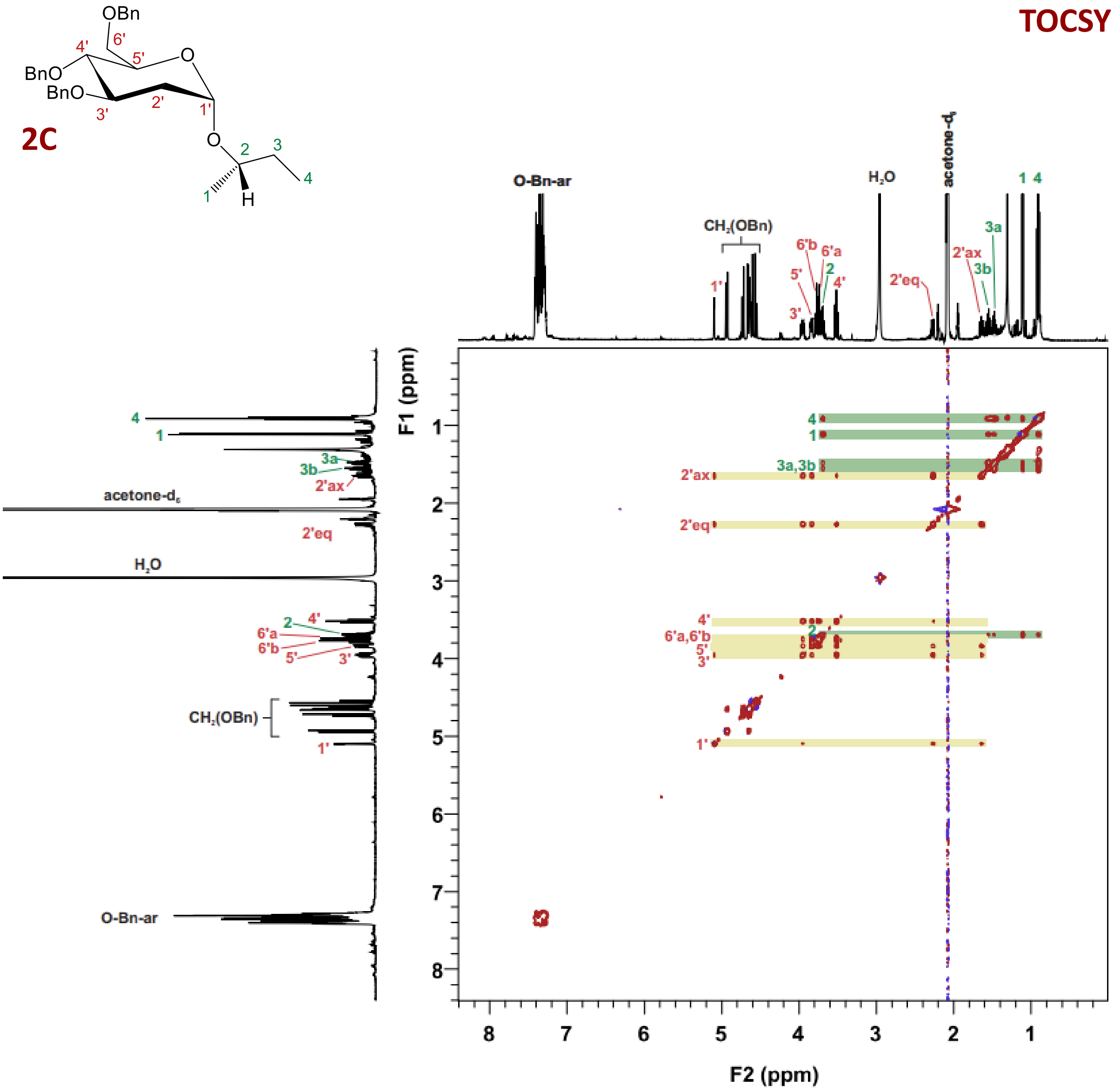

Figure.45. TOCSY spectrum for **2C**

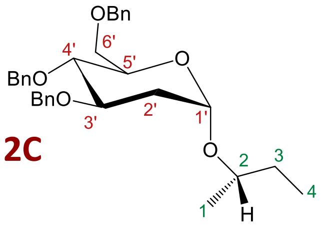

2C

HSQC

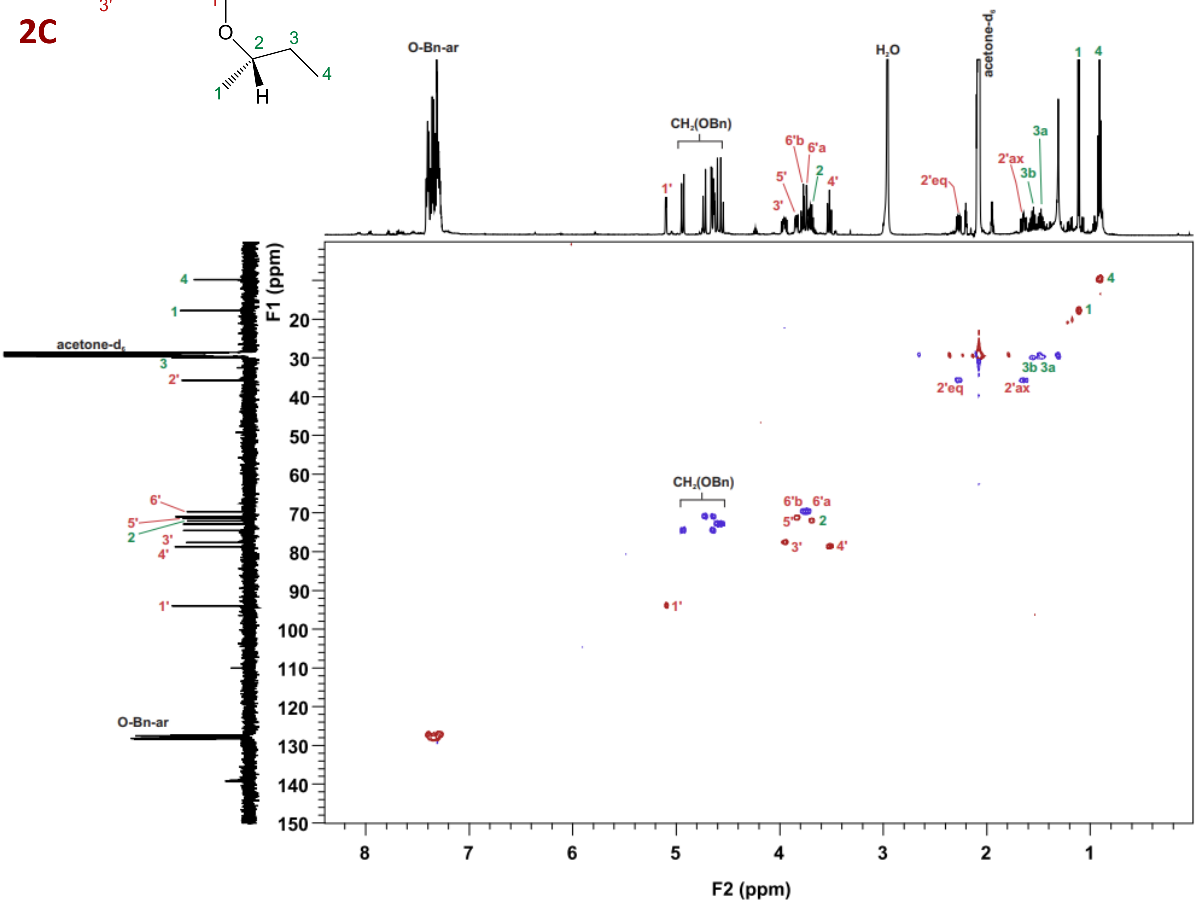

Figure.46. HSQC spectrum for **2C**

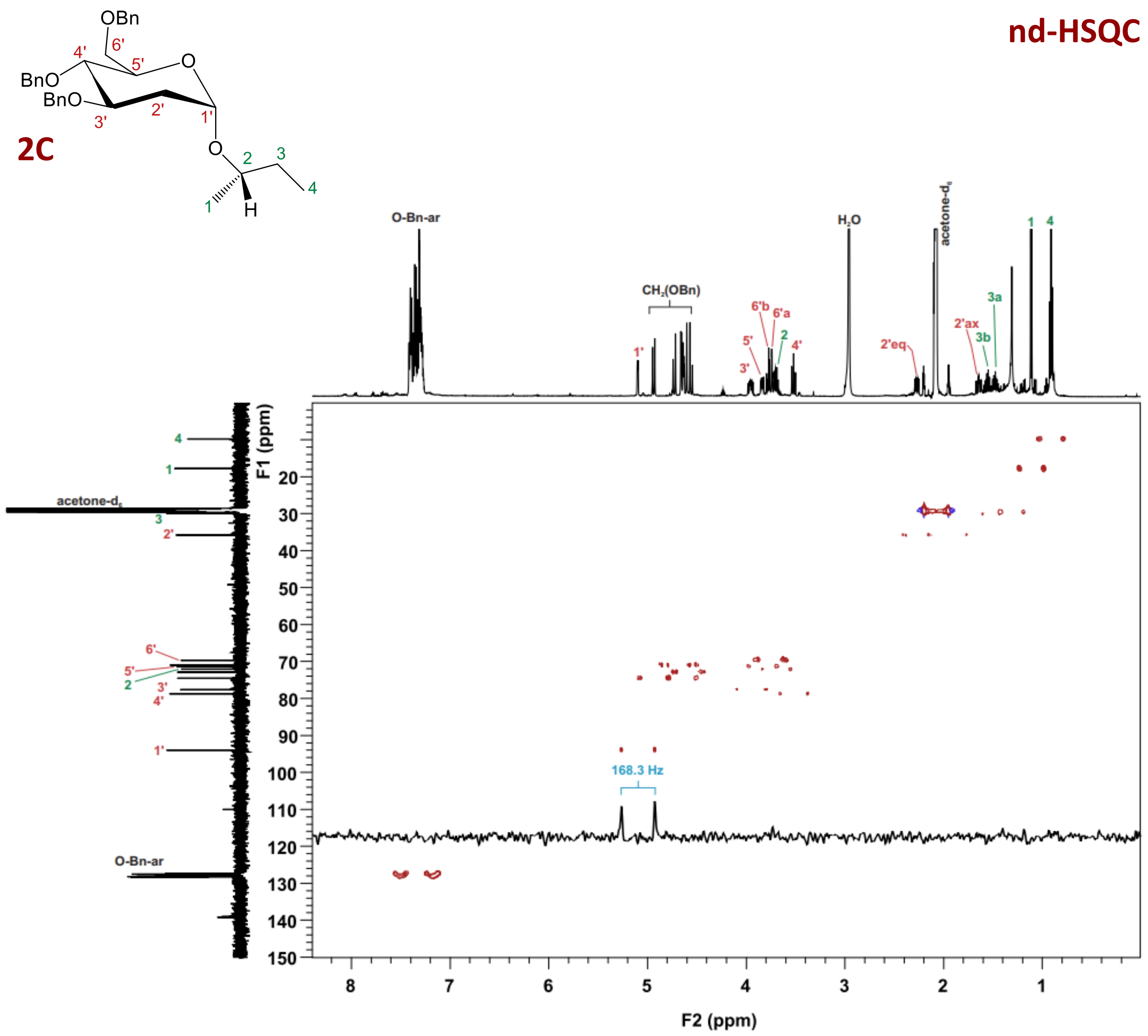

Figure.47. nd-HSQC spectrum for **2C**

Table.18. Diagnostic ROE to proton for **2C**

2D ROESY NMR Data for **2C**	
position	diagnostic **ROE to proton**
**Probe Moiety**	
**1'**	1, 2
**5'**	4

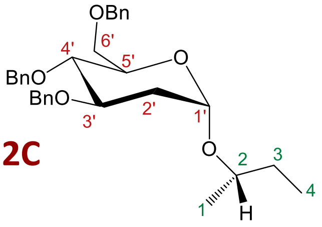

2C

ROESY

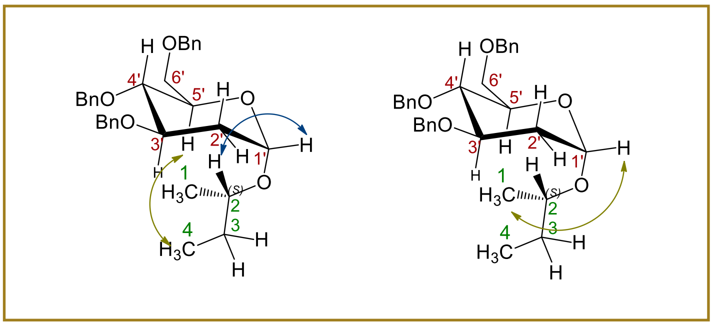

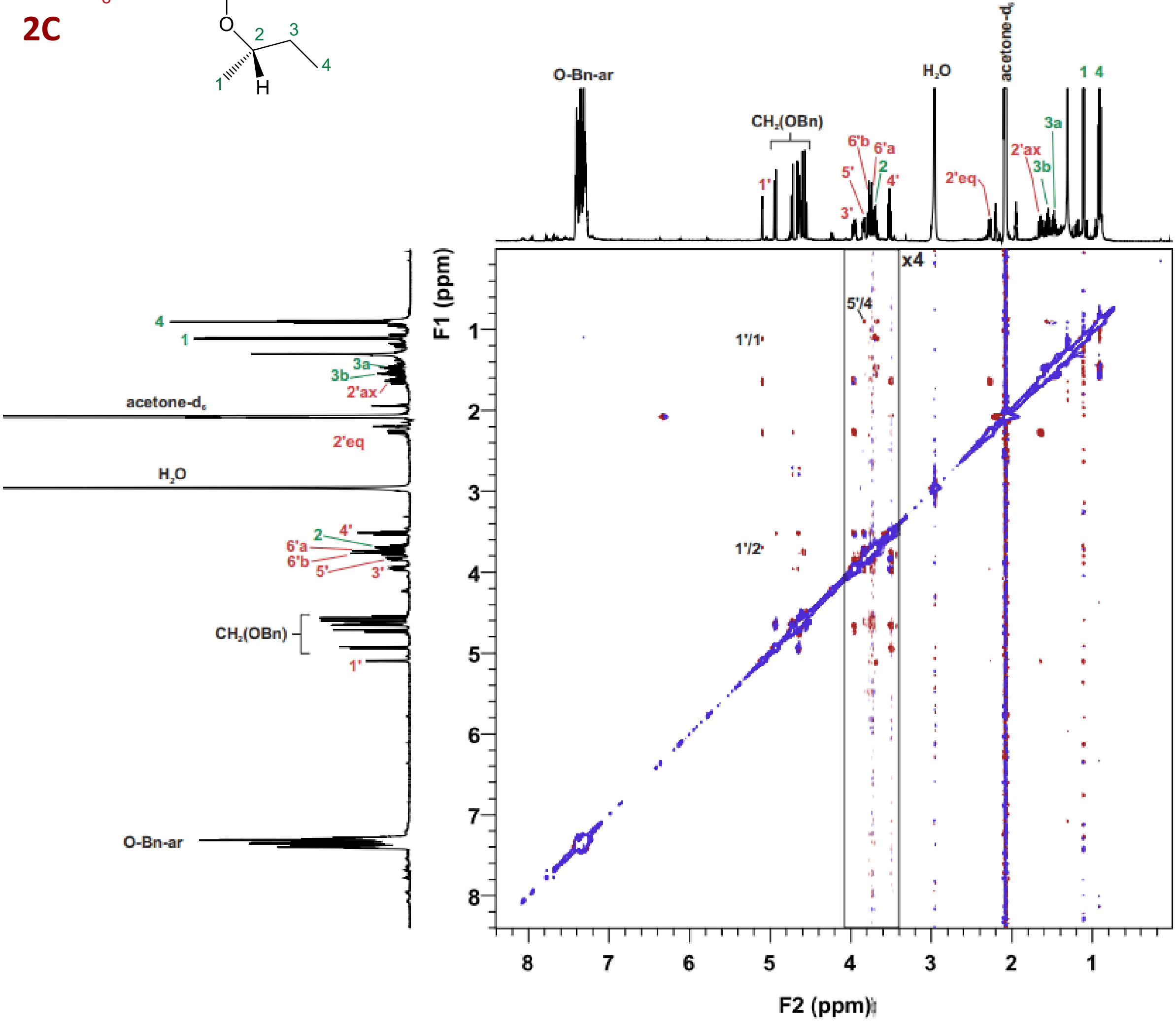

Figure.48. ROESY spectrum for **2C**

5.2. NMR experimental dietails

1A

Two-dimensional ^{1}H spectra were measured in the phase-sensitive mode with a spectral width of 4499 Hz.
gDQCOSY spectrum was acquired in a 4050 × 300 matrix with 2 accumulations per increment and was processed in a 4 K × 1 K matrix.
ROESY spectrum was collected in the phase-sensitive mode with a mix time of 200 ms in a 2700 × 330 matrix with 2 accumulations per increment, and was processed in a 4 K × 1 K complex points matrix.
zTOCSY spectrum was collected with a mix time of 80 ms in a 2700 × 300 matrix with 2 accumulations per increment, and was processed in a 4 K × 1 K complex points matrix.
gHSQCAD spectrum was acquired in the phase-sensitive mode with CRISIS-based multiplicity editing and 1J(CH) set to 146 Hz. The spectral windows for 1H and 13C of axes were 4499 Hz and 23873 Hz, respectively. The data were collected in a 1800 × 240 matrix with 2 accumulations per increment and were processed in a 2 K × 1 K complex points matrix.
The *non-decoupled* gHSQCAD data were collected in a 3600 × 240 matrix with 2 accumulations per increment and were processed in a 4 K × 1 K complex points matrix.
gHMBCAD spectrum was acquired in the phase-sensitive mode with nJ(CH) set to 8 Hz. The spectral windows for 1H and 13C axes were 4499 Hz and 25133 Hz, respectively. The data were collected in a 2970 × 250 matrix with 2 accumulations per increment and were processed in mixed (absolute value and phase sensitive) mode in a 4 K × 1 K complex points matrix.
The gH2BCAD data were collected in a 1800 × 195 matrix with 4 accumulations per increment and were processed in a 2 K × 1 K complex points matrix. The spectral windows for 1H and 13C axes were 4499 Hz and 23873 Hz, respectively.

1B

Two-dimensional ^{1}H spectra were measured in the phase-sensitive mode with a spectral width of 4612 Hz.
gDQCOSY spectrum was acquired in a 4152 × 320 matrix with 4 accumulations per increment and was processed in a 4 K × 1 K matrix.
ROESY spectrum was collected in the phase-sensitive mode with a mix time of 300 ms in a 3044 × 240 matrix with 12 accumulations per increment, and was processed in a 4 K × 1 K complex points matrix.
zTOCSY spectrum was collected with a mix time of 80 ms in a 3044 × 230 matrix with 4 accumulations per increment, and was processed in a 4 K × 1 K complex points matrix.
gHSQCAD spectrum was acquired in the phase-sensitive mode with CRISIS-based multiplicity editing and 1J(CH) set to 146 Hz. The spectral windows for 1H and 13C of axes were 4612 Hz and 18223 Hz, respectively. The data were collected in a 1660 × 170 matrix with 16 accumulations per increment and were processed in a 2 K × 1 K complex points matrix.
The *non-decoupled* gHSQCAD data were collected in a 4152 × 60 matrix with 16 accumulations per increment and were processed in a 4 K × 1 K complex points matrix. The spectral windows for 1H and 13C axes were 4612 Hz and 6912 Hz, respectively.
gHMBCAD spectrum was acquired in the phase-sensitive mode with nJ(CH) set to 8 Hz. The spectral windows for 1H and 13C axes were 4612 Hz and 22247 Hz, respectively. The data were collected in a 2306 × 200 matrix with 40 accumulations per increment and were processed in mixed (absolute value and phase sensitive) mode in a 4 K × 1 K complex points matrix.

1C

Two-dimensional ^1H spectra were measured in the phase-sensitive mode with a spectral width of 4381 Hz.

gDQCOSY spectrum was acquired in a 4382 × 400 matrix with 2 accumulations per increment and was processed in a 4 K × 1 K matrix.

ROESY spectrum was collected in the phase-sensitive mode with a mix time of 300 ms in a 2892 × 230 matrix with 16 accumulations per increment, and was processed in a 4 K × 1 K complex points matrix.

zTOCSY spectrum was collected with a mix time of 80 ms in a 3066 × 230 matrix with 8 accumulations per increment, and was processed in a 4 K × 1 K complex points matrix.

gHSQCAD spectrum was acquired in the phase-sensitive mode with CRISIS-based multiplicity editing and 1J(CH) set to 146 Hz. The spectral windows for 1H and 13C of axes were 4381 Hz and 18850 Hz, respectively. The data were collected in a 1752 × 180 matrix with 2 accumulations per increment and were processed in a 2 K × 1 K complex points matrix.

The *non-decoupled* gHSQCAD data were collected in a 3504 × 180 matrix with 2 accumulations per increment and were processed in a 4 K × 1 K complex points matrix.

gHMBCAD spectrum was acquired in the phase-sensitive mode with nJ(CH) set to 8 Hz. The spectral windows for 1H and 13C axes were 4381 Hz and 238801 Hz, respectively. The data were collected in a 2628 × 230 matrix with 2 accumulations per increment and were processed in mixed (absolute value and phase sensitive) mode in a 4 K × 1 K complex points matrix.

2A

Two-dimensional ^1H spectra were measured in the phase-sensitive mode with a spectral width of 4104 Hz.

gDQCOSY spectrum was acquired in a 3694 × 350 matrix with 8 accumulations per increment and was processed in a 4 K × 1 K matrix.

ROESY spectrum was collected in the phase-sensitive mode with a mix time of 300 ms in a 2708 × 300 matrix with 8 accumulations per increment, and was processed in a 4 K × 1 K complex points matrix.

zTOCSY spectrum was collected with a mix time of 80 ms in a 2708 × 300 matrix with 2 accumulations per increment, and was processed in a 4 K × 1 K complex points matrix.

gHSQCAD spectrum was acquired in the phase-sensitive mode with CRISIS-based multiplicity editing and 1J(CH) set to 146 Hz. The spectral windows for 1H and 13C of axes were 4104 Hz and 20106 Hz, respectively. The data were collected in a 1642 × 200 matrix with 2 accumulations per increment and were processed in a 2 K × 1 K complex points matrix.

The *non-decoupled* gHSQCAD data were collected in a 3284 × 200 matrix with 2 accumulations per increment and were processed in a 4 K × 1 K complex points matrix.

gHMBCAD spectrum was acquired in the phase-sensitive mode with nJ(CH) set to 8 Hz. The spectral windows for 1H and 13C axes were 4104 Hz and 238801 Hz, respectively. The data were collected in a 2708 × 230 matrix with 4 accumulations per increment and were processed in mixed (absolute value and phase sensitive) mode in a 4 K × 1 K complex points matrix.

2B

Two-dimensional ^1H spectra were measured in the phase-sensitive mode with a spectral width of 4350 Hz.

gDQCOSY spectrum was acquired in a 3914 × 350 matrix with 4 accumulations per increment and was processed in a 4 K × 1 K matrix.
ROESY spectrum was collected in the phase-sensitive mode with a mix time of 300 ms in a 2870 × 300 matrix with 2 accumulations per increment, and was processed in a 4 K × 1 K complex points matrix.
zTOCSY spectrum was collected with a mix time of 80 ms in a 2870 × 300 matrix with 2 accumulations per increment, and was processed in a 4 K × 1 K complex points matrix.
gHSQCAD spectrum was acquired in the phase-sensitive mode with CRISIS-based multiplicity editing and 1J(CH) set to 146 Hz. The spectral windows for 1H and 13C of axes were 4350 Hz and 20106 Hz, respectively. The data were collected in a 1740 × 180 matrix with 2 accumulations per increment and were processed in a 2 K × 1 K complex points matrix.
The *non-decoupled* gHSQCAD data were collected in a 3480 × 180 matrix with 2 accumulations per increment and were processed in a 4 K × 1 K complex points matrix.
gHMBCAD spectrum was acquired in the phase-sensitive mode with nJ(CH) set to 8 Hz. The spectral windows for 1H and 13C axes were 4381 Hz and 238801 Hz, respectively. The data were collected in a 2870 × 240 matrix with 2 accumulations per increment and were processed in mixed (absolute value and phase sensitive) mode in a 4 K × 1 K complex points matrix.

2C

Two-dimensional ^{1}H spectra were measured in the phase-sensitive mode with a spectral width of 4198 Hz.
gDQCOSY spectrum was acquired in a 3778 × 350 matrix with 8 accumulations per increment and was processed in a 4 K × 1 K matrix.
ROESY spectrum was collected in the phase-sensitive mode with a mix time of 300 ms in a 2770 × 300 matrix with 8 accumulations per increment, and was processed in a 4 K × 1 K complex points matrix.
zTOCSY spectrum was collected with a mix time of 80 ms in a 2708 × 300 matrix with 2 accumulations per increment, and was processed in a 4 K × 1 K complex points matrix.
gHSQCAD spectrum was acquired in the phase-sensitive mode with CRISIS-based multiplicity editing and 1J(CH) set to 146 Hz. The spectral windows for 1H and 13C of axes were 4198 Hz and 18850 Hz, respectively. The data were collected in a 1680 × 180 matrix with 2 accumulations per increment and were processed in a 2 K × 1 K complex points matrix.
The *non-decoupled* gHSQCAD data were collected in a 3358 × 180 matrix with 2 accumulations per increment and were processed in a 4 K × 1 K complex points matrix.

6. Molecular modeling

1A

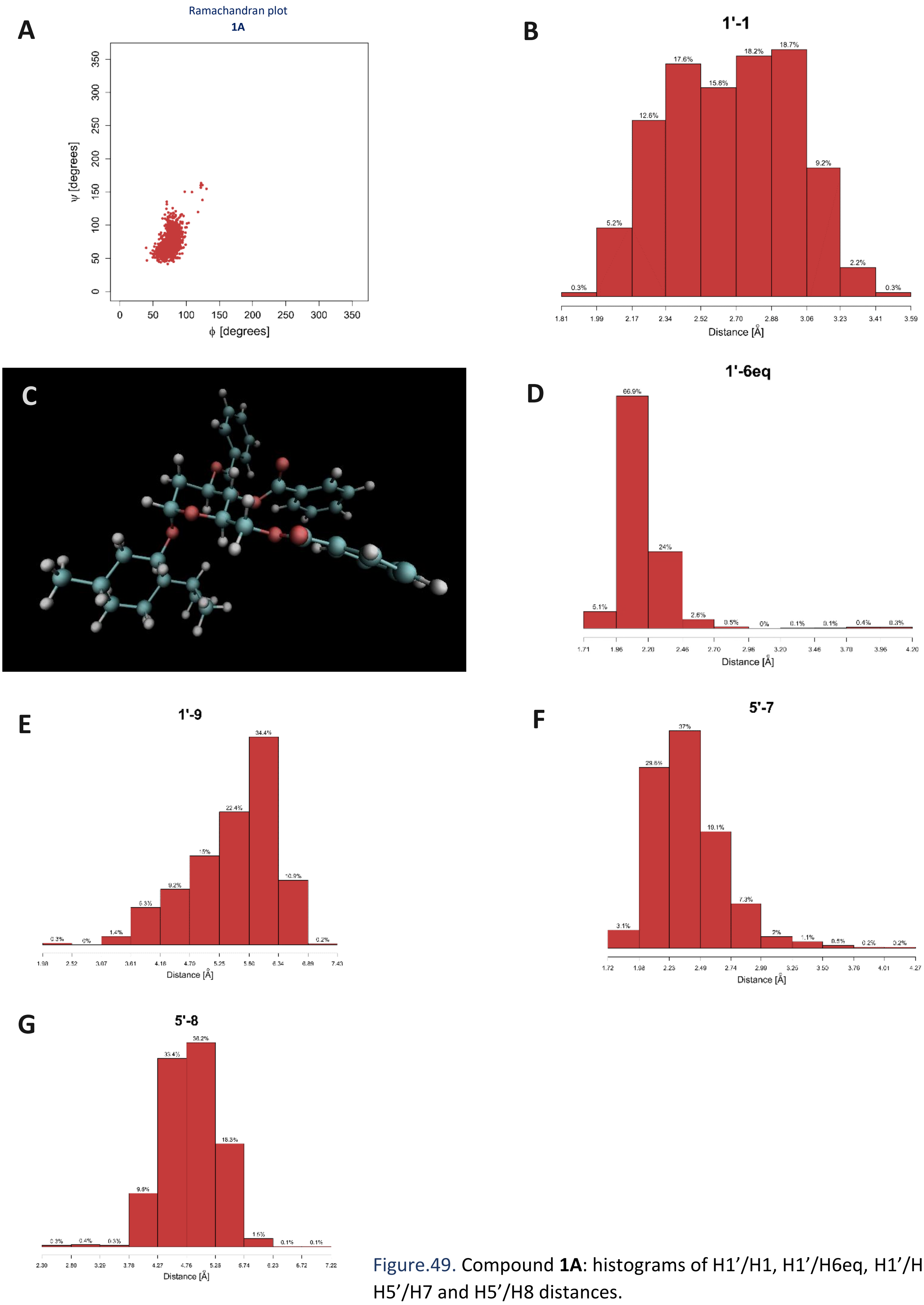

Figure.49. Compound **1A**: histograms of H1'/H1, H1'/H6eq, H1'/H9, H5'/H7 and H5'/H8 distances.

1A’ (opposite aglycone)

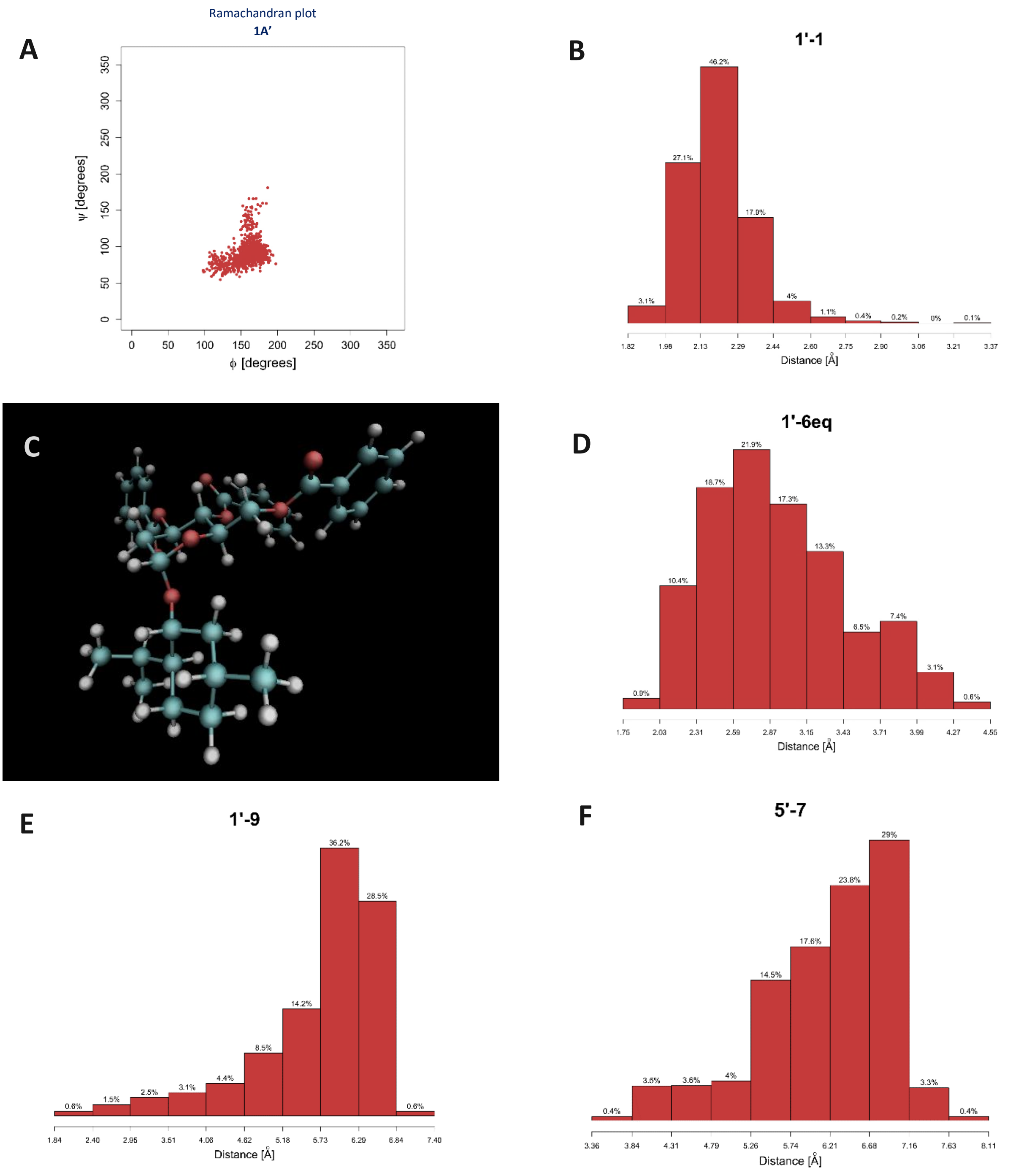

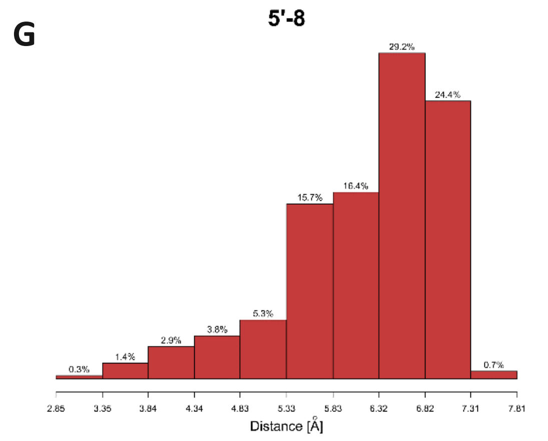

Figure.50. Compound **1A’**: histograms of H1’/H1, H1’/H6eq, H1’/H9, H5’/H7 and H5’/H8 distances

1B

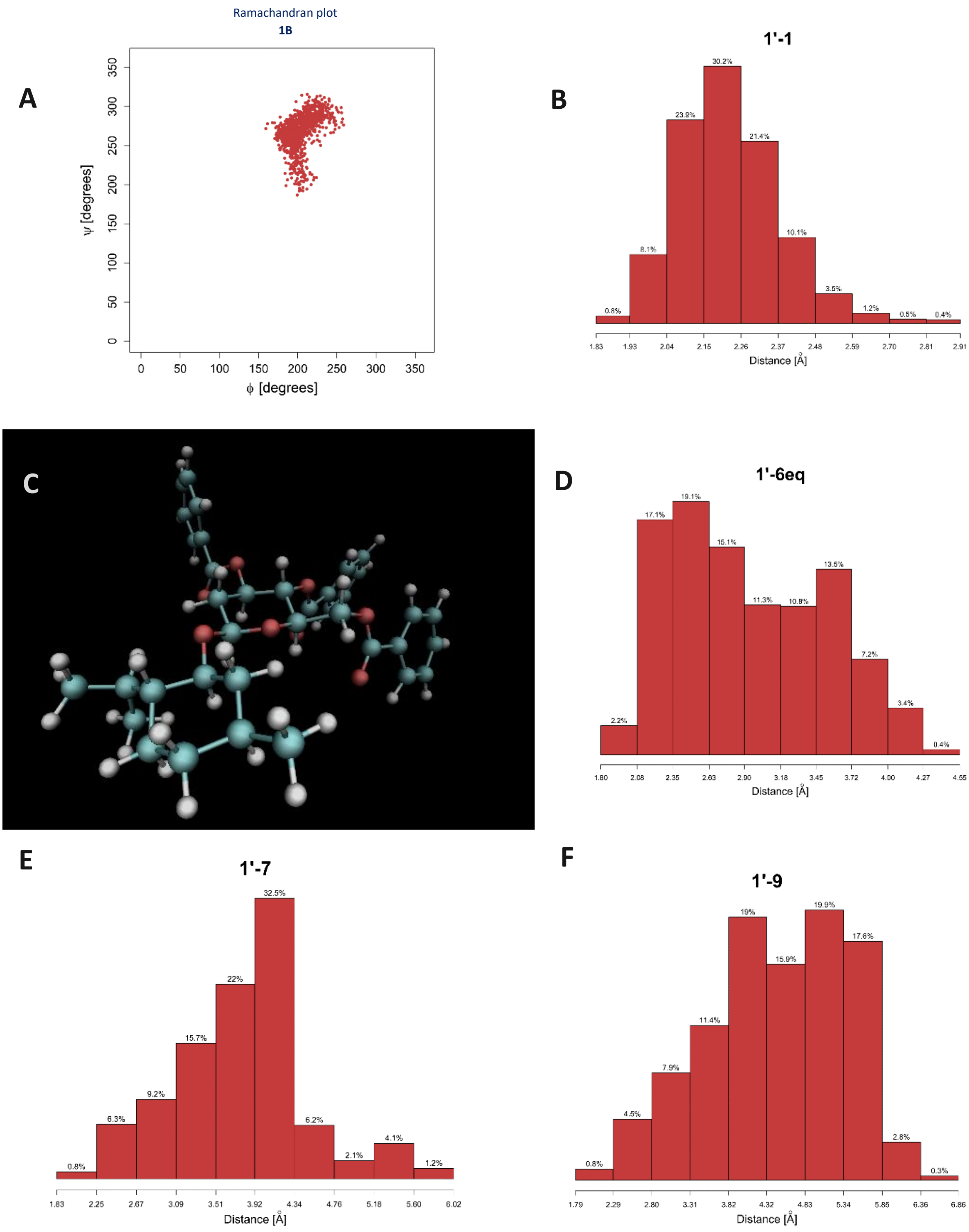

Figure.51. Compound **1B**: histograms of H1’/H1, H1’/H6eq, H1’/H7 and H1’/H9 distances.

1B’ (opposite aglycone)

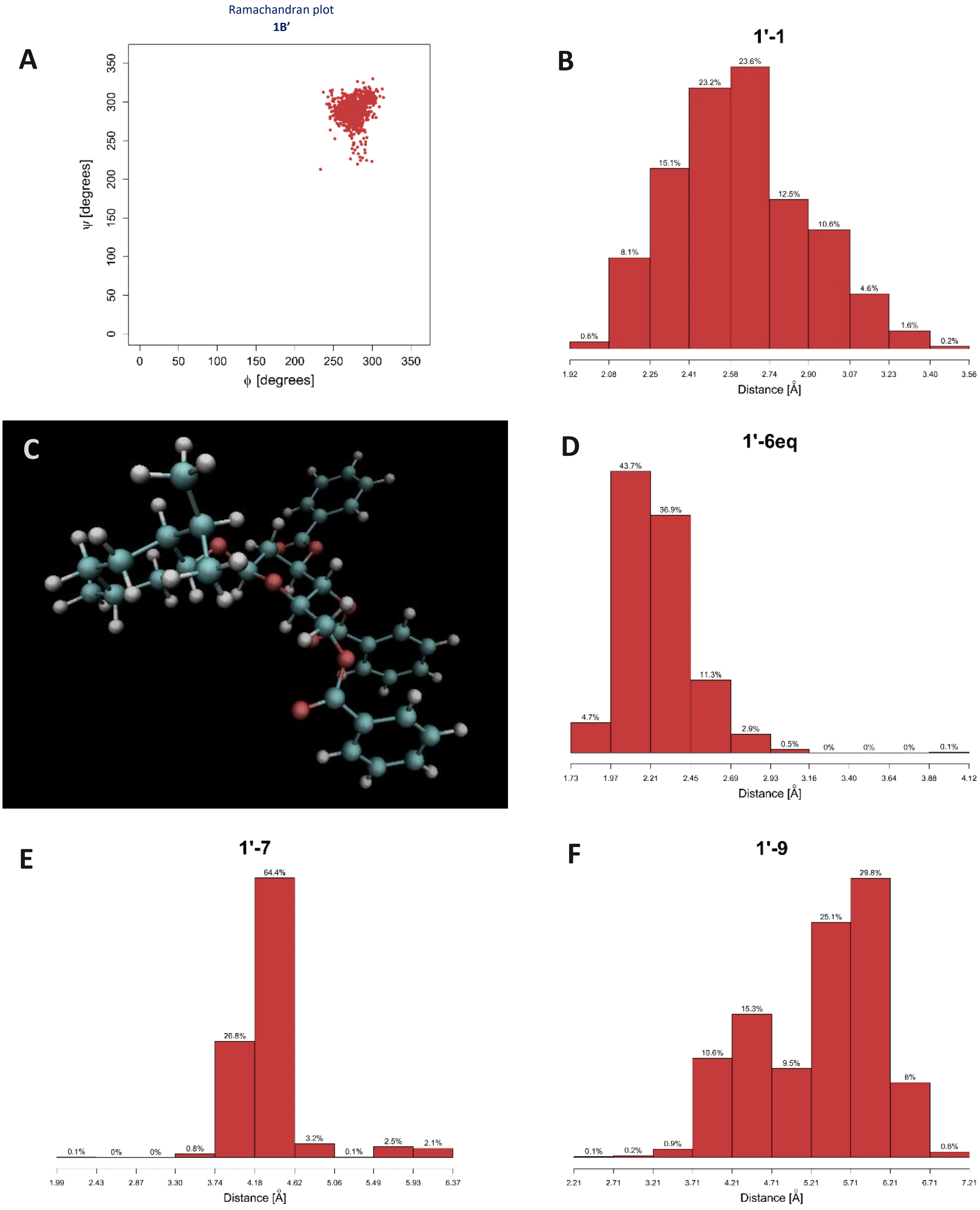

Figure.52. Compound **1B’**: histograms of H1’/H1, H1’/H6eq, H1’/H7 and H1’/H9 distances.

1C

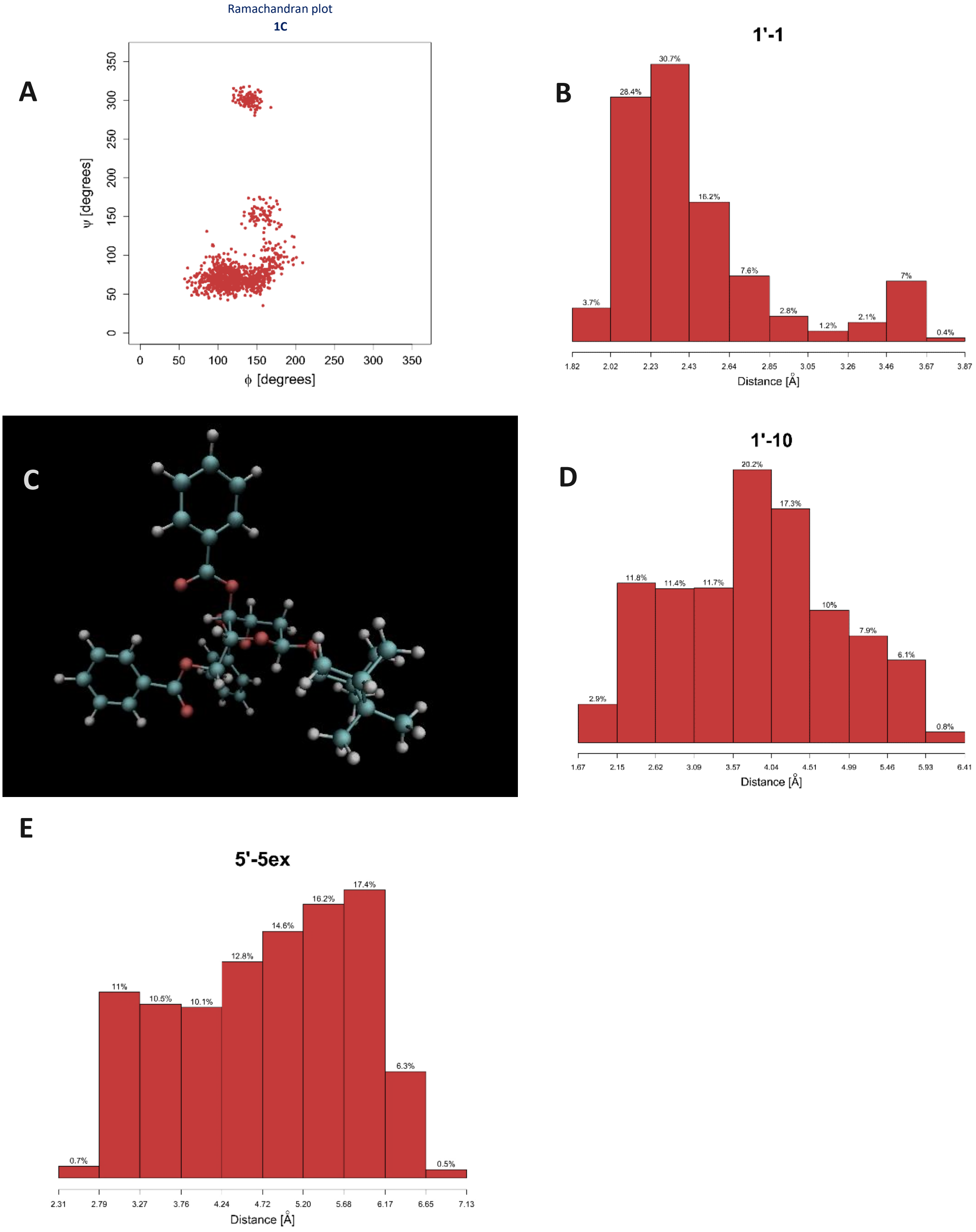

Figure.53. Compound **1C**: histograms of H1’/H1, H1’/H10 and H5’/H5ex distances.

1C’ (opposite aglycone)

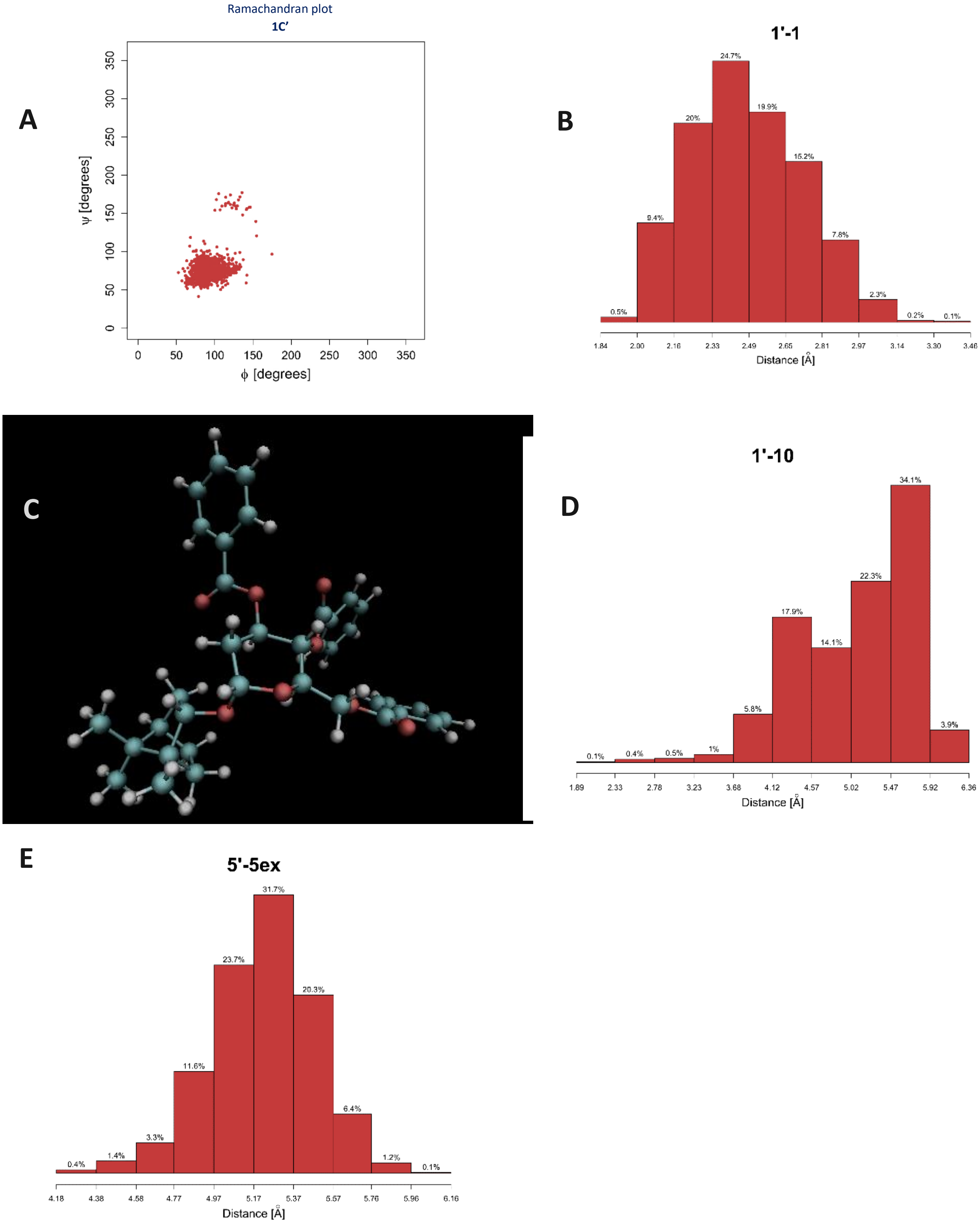

Figure.54. Compound **1C’**: histograms of H1’/H1, H1’/H10 and H5’/H5ex distances.

2A

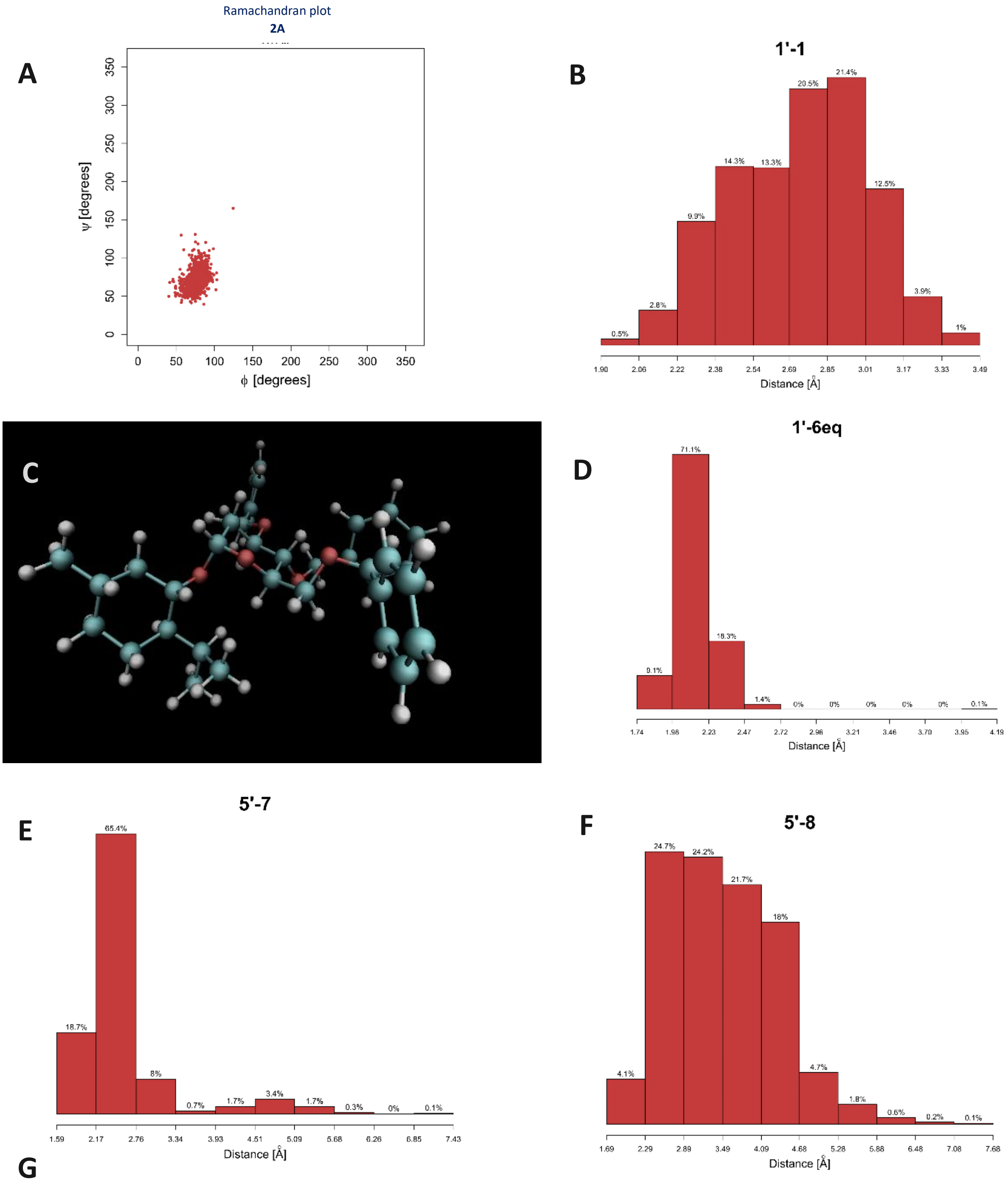

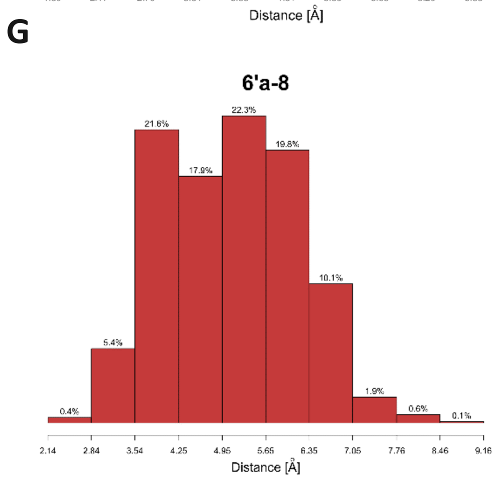

Figure.55. Compound **2A**: histograms of H1’/H1, H1’/H6eq, H5’/H7, H5’/H8 and H6’a/H8 distances

2A’ (opposite aglycone)

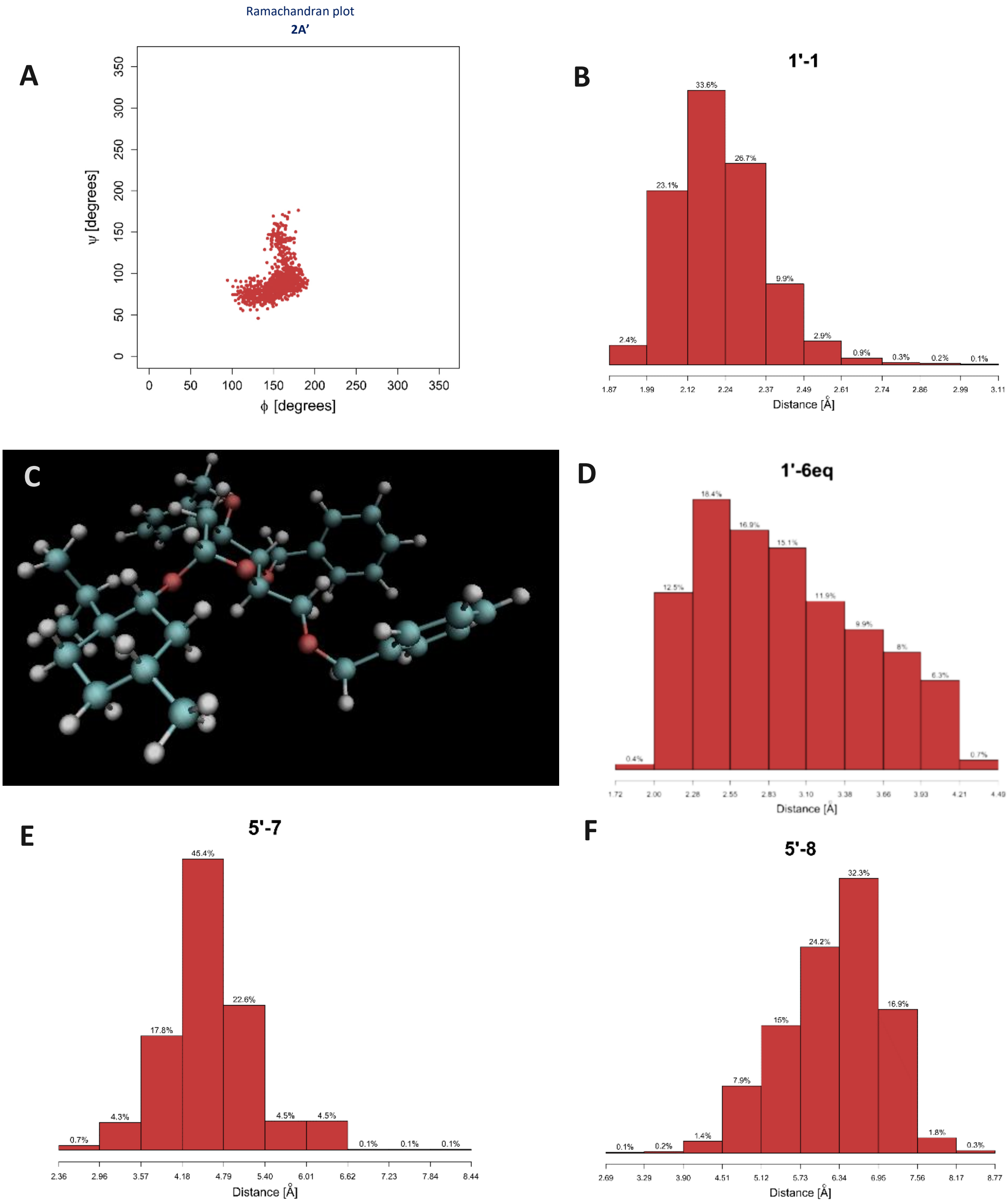

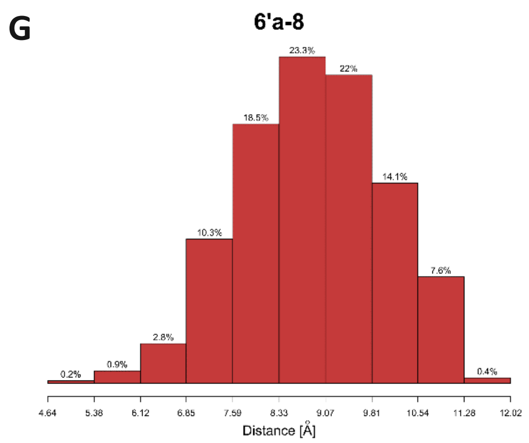

Figure.56. Compound **2A’**: histograms of H1’/H1, H1’/H6eq, H5’/H7, H5’/H8 and H6’a/H8 distances

2B

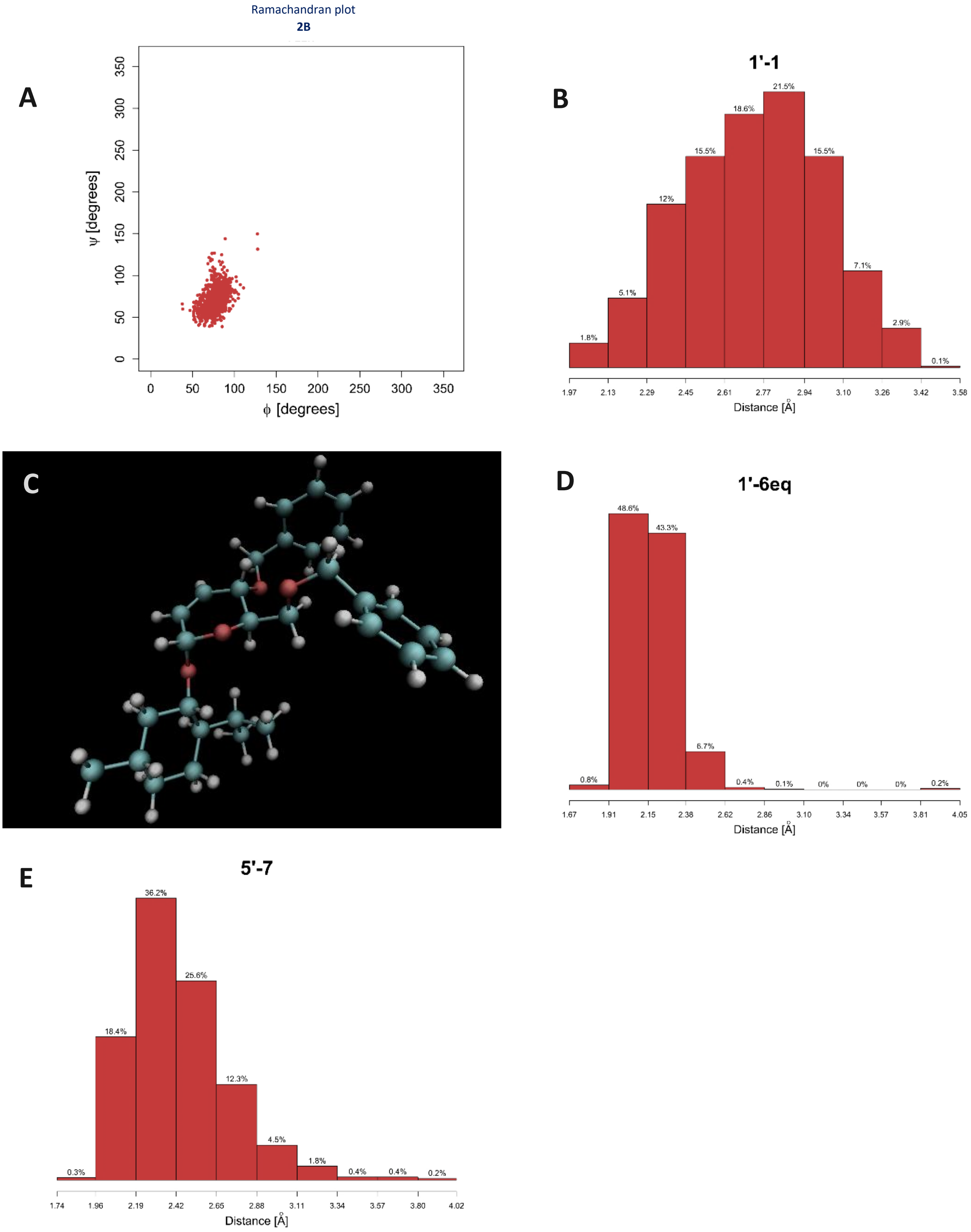

Figure.57. Compound **2B**: histograms of H1’/H1, H1’/H6eq and H5’/H7

2B’ (opposite aglycone)

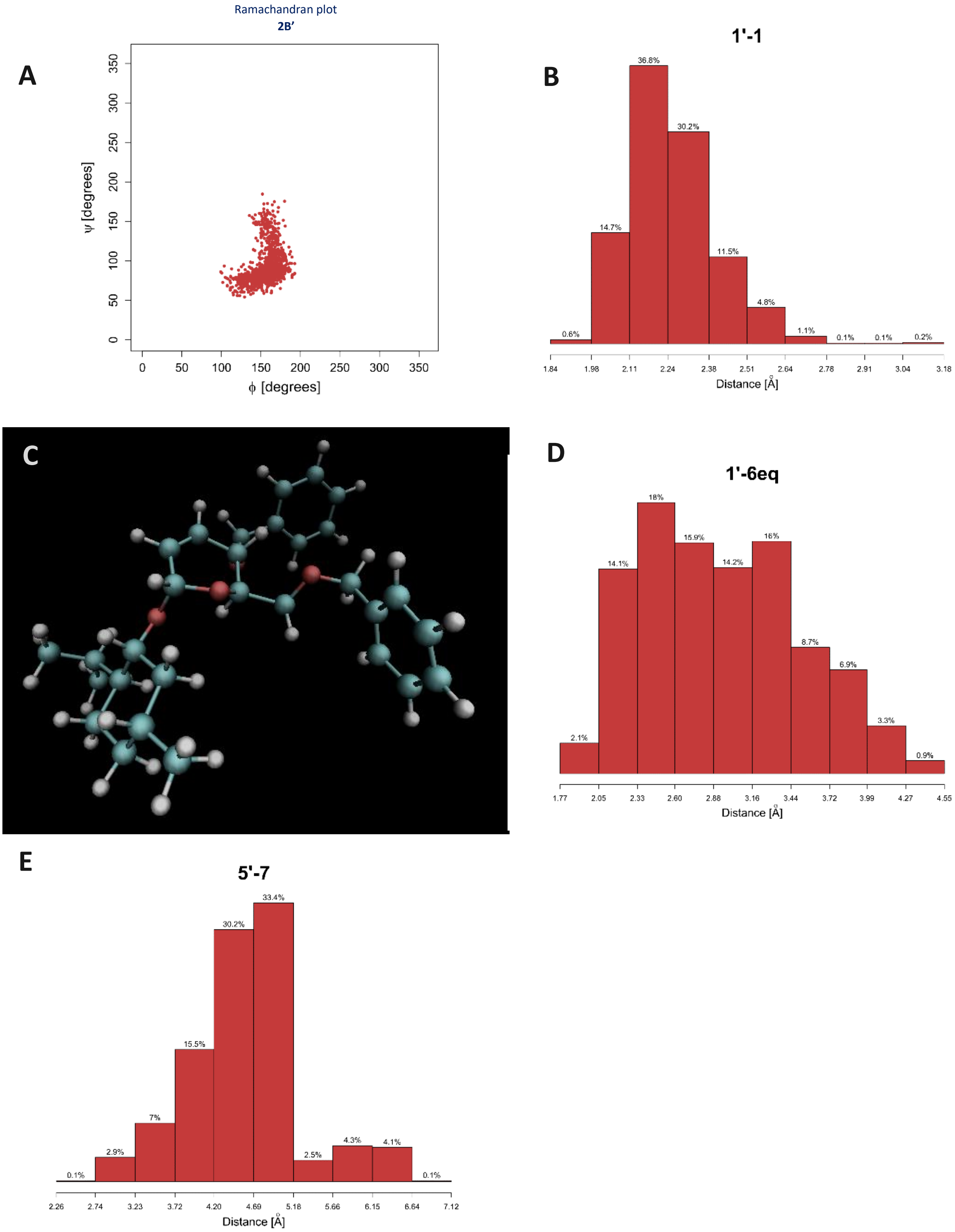

Figure.58. Compound **2B**: histograms of H1’/H1, H1’/H6eq and H5’/H7

2C

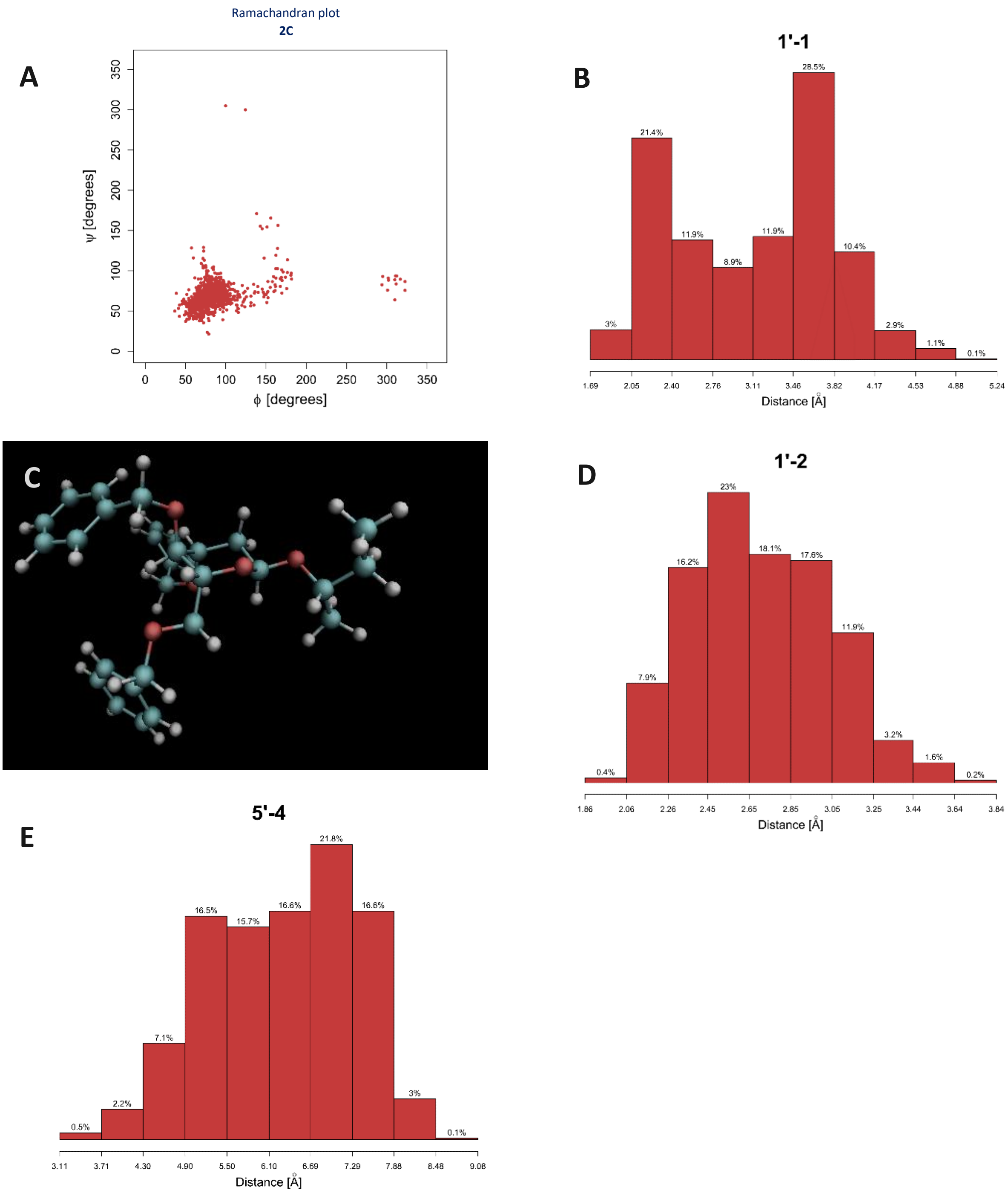

Figure.59. Compound **2C**: histograms of H1'/H1, H1'/H2 and H5'/H4

2C' (opposite aglycone)

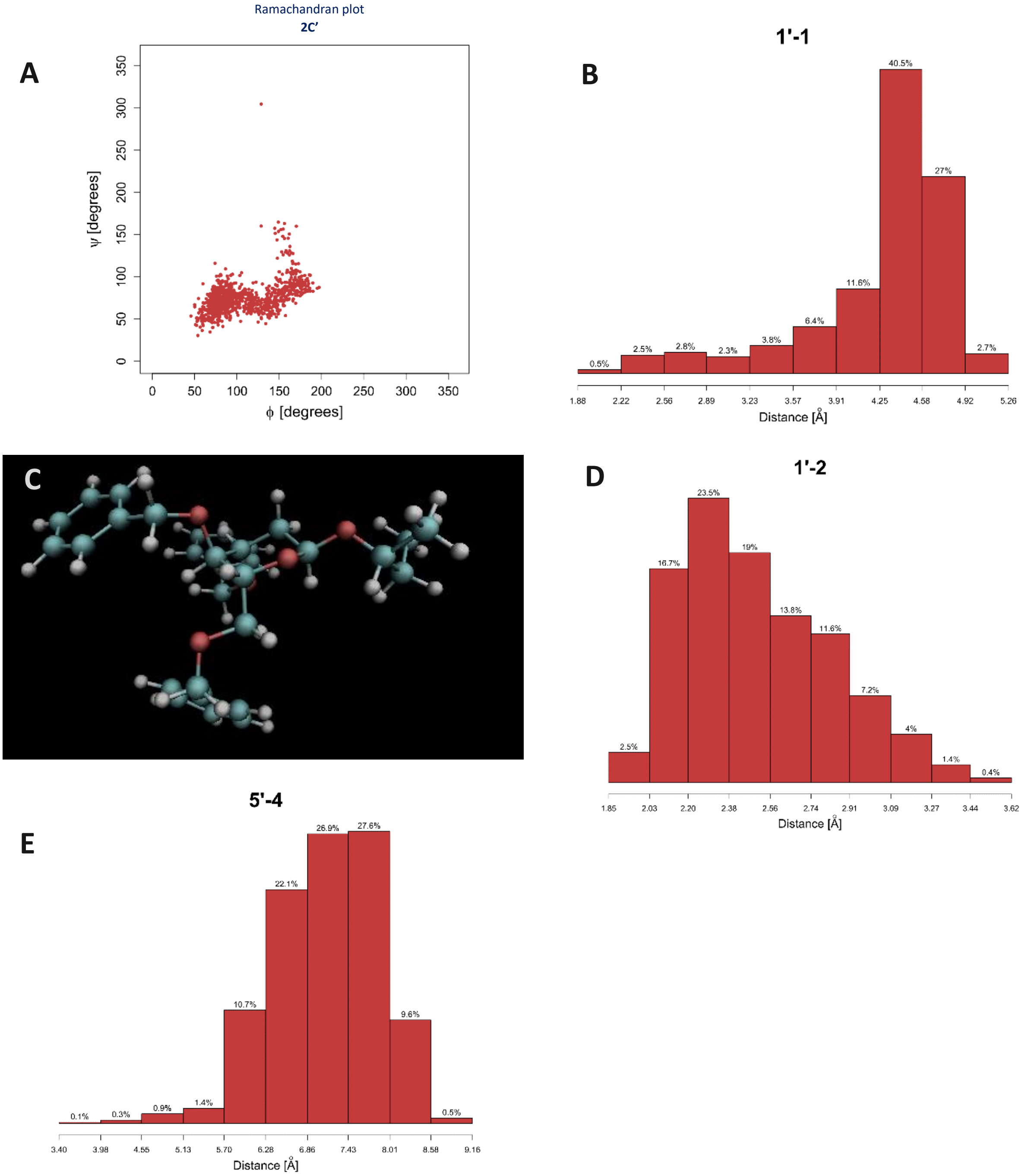

Figure.60. Compound **2C'**: histograms of H1'/H1, H1'/H2 and H5'/H4

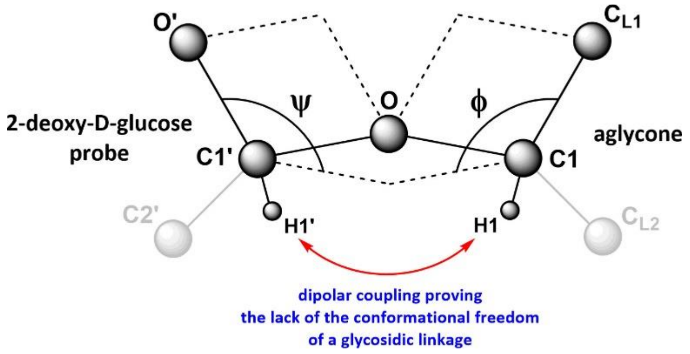

Figure.61. Definition of double-walled angles Φ and Ψ chiral probe - aglycone

7. References

[1] H. M. Corkran, S. Munneke, E. M. Dangerfield, B. L. Stocker, and M. S. M. Timmer; Journal of Organic Chemistry, 78(19), 9791-9802; **2013**
